# Human adaptation to invasive species: A conceptual framework based on a case study metasynthesis

**DOI:** 10.1007/s13280-019-01297-5

**Published:** 2019-11-24

**Authors:** Patricia L. Howard

**Affiliations:** 1 Department of Social Sciences, Wageningen University and Research Center, Hollandseweg 1, 6706 KN Wageningen, The Netherlands; 2 Centre for Biocultural Diversity, School of Anthropology and Conservation, University of Kent, Canterbury, UK

**Keywords:** Adaptation, Conceptual framework, Invasive species, Metasynthesis, Social-ecological systems

## Abstract

Species invasions are a major driver of ecological change, are very difficult to control or reverse, and will increase with climate change and global trade. Invasion sciences consider how species in invaded environments adapt, but neither scientists nor policy makers consider human adaptation to invasive species and how this affects ecosystems and well-being. To address this, a framework conceptualising autochthonous human adaptation to invasions was developed based on the Human Adaptation to Biodiversity Change framework and a case study metasynthesis. Results show that adaptation occurs within different spheres of human activity and organisation at different social-ecological scales; responses have feedbacks within and across these spheres. Adaptation to invasives and other drivers is a set of highly contextual, complex, non-linear responses that make up pathways pursued over time. Most invasive species management and adaptation occurs ‘from below,’ and policies and planned control efforts should support autochthonous adaptation, rather than undermining it.

**Electronic supplementary material:**

The online version of this article (10.1007/s13280-019-01297-5) contains supplementary material, which is available to authorized users.

## Introduction

Invasive alien species (IAS) are one of the five most important drivers affecting nature, and the fourth most important direct driver of species extinctions (Butchart et al. 2019; Ichii et al. 2019). Frequently cited estimates of economic damage provoked by IAS are in the realm of US$1.4 trillion annually (5% of global GDP) (Pimentel et al. 2001). Global progress has been insufficient to reduce their spread. Numerous international conventions and global, regional, and national policies and programmes are directed toward preventing, controlling or eradicating IAS, but efforts to mitigate their impacts are also insufficient (Butchart et al. 2019). Species invasions can be exceedingly difficult to reverse (Kettenring and Adams 2011; Bhagwat et al. 2012; Pearson et al. 2016; Kitunda 2018), and large-scale invasive control programmes carry substantial risks (Kopf et al. 2017). Species invasions are likely to increase on a par with climate change and international trade (Shin et al. 2019). All of this indicates that, as is the case with climate change, invasive species inevitably provoke change in biodiversity and in the ecosystem services that it provides, and people must adapt.

The need for adaptation is a central tenant of climate change science and policy, as adaptation ‘seeks to moderate or avoid harm or exploit beneficial opportunities…[where] human intervention may facilitate adjustment’ (IPCC 2014). However, while invasion biologists and ecologists consider how invasive species adapt to new environments and how other species adapt to invasions (Mooney and Cleland 2001), neither scientists nor policy makers consider human adaptation to invasive species (HAIS), or how such adaptation might affect ecosystems and human well-being. A major reason for this omission is that the invasion sciences are generally focused on natural and semi-natural ecosystems and largely exclude human-dominated ecosystems, or anthromes.^1^ Another is that invasion sciences have notoriously discounted the significance of invasive species for human livelihoods and well-being, particularly among populations that directly depend on biological resources and in developing regions, where impacts may be most severe (Shackleton et al. 2019b). In these regions, invasives ‘may also cause social instability and economic hardship placing curbs on substantial development, economic growth and food security,’ that is, their effects may be much more complex (Khadka 2017, p. 2). Other plausible explanations for the lack of attention to HAIS are beyond the scope of this current work (but see Howard 2009; Vaz et al. 2017). Rather, here the aim is to begin to address this major knowledge gap.

An interdisciplinary project sought to develop conceptual frameworks, methods and tools to begin to explore human adaptation to biodiversity change (HABC)^2^—the theme of this Special Issue (Howard and Pecl 2019). The project strategically opted to focus on IAS as a driver of biological change. Biologists and ecologists view invaded areas as ‘experimental stations’ where processes of co-evolution and ecological change are speeded up and can be observed in real time (Sax et al. 2007); thus, it can be inferred that people’s responses to such change can also be observed in real time. The project selected to focus on one of the ‘100 world’s worst’ invasive species, *Lantana camara* (Bhagwat et al. 2012), testing concepts, methods and tools through fieldwork in the Western Ghats of India (Kent and Dorward 2014; Puri 2015; Thornton et al. 2019). The current research enlarged the empirical basis of this work through a metasynthesis of global literature and further elaborated the conceptual framework on human adaptation to invasive species (HAIS). Here, the HAIS framework is first presented conceptually. Subsequently, each component is discussed, illustrated and analysed empirically (the full quantitative and qualitative analysis and findings of the metasynthesis will be published separately). To ensure reproducibility and the utility of the conceptual framework for future research, all components and definitions are presented in supplementary tables.

The focus of the HAIS framework is on ‘autochthonous adaptation’. It can be readily deduced that the majority of terrestrial species invasions across the globe that directly impact people are managed at local scales. This includes management on indigenous lands, which constitute over a quarter of the world’s terrestrial area (Garnett et al. 2018), and of most terrestrial areas in low and lower middle-income countries. ‘Top-down’ or ‘planned’ invasive species prevention and management is highly concentrated in high- and middle-income countries (e.g. Cock et al. 2016; Cock and Kuhlmann 2017; Willis 2017) and on uninhabited islands (Glen et al. 2013), where IAS research is also concentrated (Pyšek et al. 2008; McGeoch et al. 2010; Bellard and Jeschke 2016; Yu et al. 2016). The efficacy and cost effectiveness of many control programmes has generally been found to be limited (e.g. Hoffmann and Broadhurst 2016), and some have generated more harms than benefits (e.g. Middleton 1999; Kopf et al. 2017; Bean and Dudley 2018; Kitunda 2018). Most planned control efforts are implemented at least in part by local people (e.g. Simberloff 2003; Bennett and van Sittert 2019) which, as shown below, can itself provoke adaptation. Given all of the above, HAIS is not only occurring across most of the invaded terrestrial areas across the globe—it also ‘increasingly represents the only available option’ (Barnes et al. 2014, p. 327).

The HAIS framework deals with ‘autochthonous adaptation,’ a concept that was developed for this framework. It is rooted in concepts of cultural adaptation (Ellen 2018), and is defined here as “deliberate adaptation actions undertaken by individuals or small social groups that are specific to and occur within a local system, where human populations are ultimately affected.” As Howard and Pecl (2019) note, it contrasts with mainstream concepts of ‘autonomous’ adaptation, and has four distinct dimensions: “(1) it is deliberate, (2) it refers to individuals and small groups of individuals; (3) it is specific to the locality—specific environmental, social and cultural conditions that prevail in specific places where people live and act and (4) it occurs within a local system, which is affected by multi-scalar drivers and feedbacks.” It also contrasts with ‘planned adaptation,’ which is the result of the policy decisions of supra-collectivities, such as states (IPCC 2007).

### The state-of-the-art: Adaptation and social-ecological systems theory in invasion science

HAIS *is* a research topic within invasive sciences but it is *not* a ‘scientific problem’—a field of theorising or methods development.^3^ Attention is increasingly focusing on social dimensions of biological invasions and on developing conceptual frameworks in order to, for example, better capture the diversity of values and perceptions that underlie conflicts in invasive species management (Estévez et al. 2015; Shackleton et al. 2019a), prioritise prevention and control efforts considering both socio-economic and ecological impacts (Bacher et al. 2018) and enhance invasive species management by accounting for positive as well as negative effects for humans and ecosystems (Pienkowski et al. 2015). Some researchers are concerned with the effects of invasives and invasive management for human well-being and livelihoods, particularly of highly biodiversity reliant rural people who have largely been omitted in research and policy (Shackleton et al. 2007; Monterroso et al. 2011; Shackleton et al. 2019b).

Such efforts have to date largely failed to incorporate insights from the adaptation to environmental change literature, or from the case study research presented here (see footnote 3). The invasion sciences have also neglected social-ecological systems theory, which some argue is required to advance both invasion science and its practical application (Chaffin et al. 2016; Vaz et al. 2017). A recent exception is Shackleton et al.’s (2018) review of social-ecological systems concepts, including drivers and feedbacks, applied to four case studies of invasive-driven regime shifts and their consequences for livelihoods and well-being. Their work shows how an understanding of the complex dynamics of regime shifts can be used to enhance adaptive governance, highlight consequences for different environments and stakeholders, and develop methods to enhance resilience and reduce vulnerability. While their work presents a major advance in framing complex human-ecological dynamics in invasion science, human adaptation to invasives is not considered either as a conceptual construct or a process (as in e.g. Bassett and Fogelman 2013; Wise et al. 2014).

The HAIS framework is also loosely based on social-ecological systems theory, and the axiom that humans and ecosystems are intertwined. Fundamental concepts in both frameworks include social-ecological system drivers and feedbacks, resilience and regime shifts (Holling and Gunderson 2002; Folke et al. 2010). People respond to different, often interacting, social-ecological drivers and feedbacks. Below, it is shown that responses to IAS occur within different spheres of human activity and organisation (e.g. productive activities, households, resource management systems) and at different social-ecological scales (e.g. micro- and meso-level institutions); responses have feedbacks within and across these spheres. Further, adaptation is best understood as a set of highly contextual, complex, non-linear responses that make up *pathways* pursued over time. Different social groups have different adaptation options and pathways, depending upon their spheres of activity and forms of organisation, assets (including knowledge and power), constraints and opportunities. Pathways involve different adaptation *types* (e.g. mobility, diversification) that have different feedbacks and outcomes in terms of social-ecological system resilience and regime shifts. Invasive species impact ecosystem health, which can also impact human well-being—when well-being is affected, humans respond to change these impacts. Human responses affect both invasive feedbacks and drivers of invasion with both intentional and unintentional consequences for ecosystems and human well-being. These changed dynamics and outcomes feed back into adaptation pathways and may change these. Both invasions and human adaptation can lead to regime shifts and purposive transformation.

## Materials and methods

The HAIS framework and results are based on a qualitative case study metasynthesis, which permits comprehensive analysis of a topic as well as the development of theoretical models (or conceptual frameworks) based on primary qualitative study findings (Major and Savin-Baden 2011). The information source is qualitative case study research, which investigates and analyses single or bounded multiple cases to capture complexity in real-life situations. This review applied qualitative content analysis, which systematically classifies and codes textual data, allowing identification of themes or patterns (Hsieh and Shannon 2005). Case studies were selected that met the criteria presented in Table 1.

**Table 1:** Case study selection criteria

Criteria	Included	Excluded
Well-being impact	Invasive species present and impacting well-being	Human well-being not considered as a driver or outcome
Local-scale	Detailed examination of processes as they unfold at fine scales of resolution, where most adaptation occurs	Large-scale reviews, e.g. of variable management or use across countries and continents
Autochthonous	People taking direct, largely self-determined actions to respond to invasives and impacts	Externally-driven, or ‘collaborative’ management. Too few case studies (9) with great variability in forms of management and focus, with few common variables; co-management also often ‘externally led’
Contemporary	1940s onward, largely post-colonial to ensure relevance for contemporary research and policy, and greater case comparability	Historical case studies (pre-1940s)
Adaptive	Adaptation as a process, including drivers, impacts, responses and outcomes, or most of these elements	Cases focused on a single or a few dimensions, e.g. perceptions and control, or use and livelihoods, omitting processes
Study quality^a^	Methodological rigour/coherence	Methods limitations and deficits that can bias results and their interpretation; failed to adequately document methods

^a^See e.g. Petticrew and Roberts (2006)

### Developing the conceptual framework categories

The HABC conceptual framework^4^ provided the initial set of categories (or concepts) and definitions. Studies were reviewed for relevant text corresponding to these. In this process, a hierarchy of new sub-categories related to change and adaptation began to emerge, and some of the initial categories were redefined (in grounded theory, termed ‘Axial coding’) (Böhm 2004). Cross-case analysis was then performed in two steps. The first textually synthesised cases on the same invasive species, resulting in further Axial coding. Then, to analyse data across all species and cases, a second variable-oriented analysis was performed using ‘selective coding’—placing all relevant textual information into the pre-existing categories, while expanding and adapting these categories as required to incorporate any new variables that repeated across two or more cases. All coded qualitative data were entered into Excel. To ensure that categories were empirically relevant (e.g. for all categories other than drivers, these did not represent ‘one-off’ instances), and to analyse trends in the data and possible reasons for these, data were coded into frequencies (1 = mentioned, 0 = not mentioned), and descriptive statistics were generated. Because frequencies are simple counts of variable presence or absence, data such as the direction or magnitude of change are lost. Therefore, qualitative data are used to supplement and illustrate. Single textual examples are provided throughout the results to illustrate the framework categories and sub-categories and their interrelations. Frequencies are presented for a few of the central categories (e.g. invasive impacts, adaptation pathways), whereas multiple examples of adaptation pathways, feedbacks and outcomes are presented in the tables, figures and text.

### Literature search and results

An iterative search methodology was applied to isolate case studies on species invasions with relevant social content using the search terms presented in Table 2. ‘Well-being’ terms captured human impacts of invasives and ‘population groups’ captured potential adaptors (‘local population groups’). Searches were delimited to subject areas that could refer to human beings engaged in ‘adaptable’ (livelihood-related) activities.

**Table 2:** Scopus search terms (title, abstract, keywords), subject area and document delimiters (English language, no temporal restrictions)

Invasion/invasive	Well-being terms	Population groups
Ecological invasion*, biological invasion*, invasion biology, invasion ecology, invasive species, invasive alien, alien species, alien invasive*, introduced species, non-native species, nonnative species, nonindigenous species, allochthonous species, exotic species	Livelihood*, subsist*, income, poverty, wealth, standard of living, economic conditions, quality of life, well-being, wellbeing, well being	Local commun*, urban commun*, coastal commun*, agricultural commun*, subsistence commun*, rural commun*, fishing commun*, rural population*, rural people*, rural producer*, agricultural workers, farmer*, peasant*, subsistence producer*, fishermen, fishers, fisherfolk, hunter*, gatherer*, fishing household*, fisheries household*, farming household*, agricultural household*, rural household*, indigenous people*, indigenous commun*, indigenous population*, trib*, ethn*, aborigin*, native people*, minorit*

Subject area limits: limit to	Document type limits: limit to
Agricultural and biological sciences, Environmental science, Social sciences, Multidisciplinary, Veterinary, Arts and Humanities, Economics, Econometrics and Finance, Energy, Decision Sciences, Business, Management and accounting; Undefined	Article; Article in Press; Review; Conference Paper; Book Chapter; Note; Short Survey; Letter; Book; Editorial; Report

*Searches were performed on the search terms grouped as a set (using ‘OR’); subject areas and document types were also delimited as a set

The first set of searches was performed on ‘invasive species’ in general; a second set was performed on specific invasive species. As indicated in Table 2, search terms captured any ‘invasive species’ regardless of native or non-native origin. Scopus yielded a higher number of relevant citations compared with Web of Science. The first set of Scopus searches (January 2018) were screened, yielding 25 case studies. Then, searches were performed for each of the Global Invasive Species Database (GISD)^5^ ‘100 worst invasives’ (February 2018) (for GISD search results, see Table S2) and for *Prosopis juliflora* and *Pteridium aquilinum*, which were the subjects of previously-identified case studies. A few additional citations were obtained through case study references. Figure S1 presents the PRISMA flow diagram (Moher et al. 2009) for the search results. The invasive species that were the topics of the case studies represented 11% of all Scopus citations on the GISD ‘100 Worst Invasive Species’ list, including 21% of all cites with ‘well-being’ or ‘population group’ content, and 39% of the cites with confirmed (after screening) socio-economic content (Table S2). Therefore, the selected case studies represent 100% of the literature on adaptation to invasives that could be located using the search criteria. The studies identified are listed in Table S3 (citations and case clustering). Five articles provided sufficient data on multiple cases to merit analysis as separate cases, while several articles reporting on the same invasive in the same geographical area allowed these to be analysed as single cases. In a few cases, additional articles focused on the same study area provided important supplemental data.

As is the case with most other invasive literature reviews and metasyntheses, this review is also likely to be geographically and taxonomically biased, as Scopus does not cover many publications from developing region journals, conferences and grey literature. HAIS case studies are mainly related to agricultural and pastoral systems in developing countries, a bias that is mirrored in the local climate change adaptation literature (Wise et al. 2014). ‘Invasive species’ search terms capture only a small fraction of the literature on invasive species,^6^ and the GISD focuses exclusively on ‘invasive alien species that threaten native biodiversity and natural areas.’^7^ In distinction, CABI’s Invasive Species Compendium covers a much larger number of invasives ‘threatening livelihoods and the environment worldwide,’^8^ but contains too many species to investigate individually. Cases of co-management were omitted (but see Graham et al. 2019 for a review) as well as some excellent historical case studies (e.g. Middleton 1999; Frawley 2014) that may still have some contemporary relevance. Finally, none of the selected case studies provided data on all conceptual framework elements, and spatial and temporal scope and population numbers varied considerably, which restricted some of the analysis presented here to a sub-set of cases.

Most of the cases focused on Asia (40%) and Africa (30%), with a minority located in North America (14%) and the Pacific (10.5%). No urban case studies were located. Most cases dealt with species found on the GISD ‘100 worst’ list (Table S2), including *Imperata cylindrica* (cogon grass) (21% of cases), *Chromolaena odorata* (16%) and *Lantana camara* and *Pomacea canaliculata* (golden apple snail) (7% each). Two case study species are not on the GISD list: *Prosopis juliflora* (mesquite) (9% of cases, 22% of articles) and *Pteridium aquilinum* (bracken fern) (7% of cases, 6% of articles). While 68% of the species were exotic and introduced, nearly a third were native invasives, which also reflects invasive management as it actually occurs across the globe (Diaz-Soltero and Scott 2014; Buckley and Catford 2016; Nackley et al. 2017).

## Results

### HAIS conceptual framework overview

**Fig. 1:**
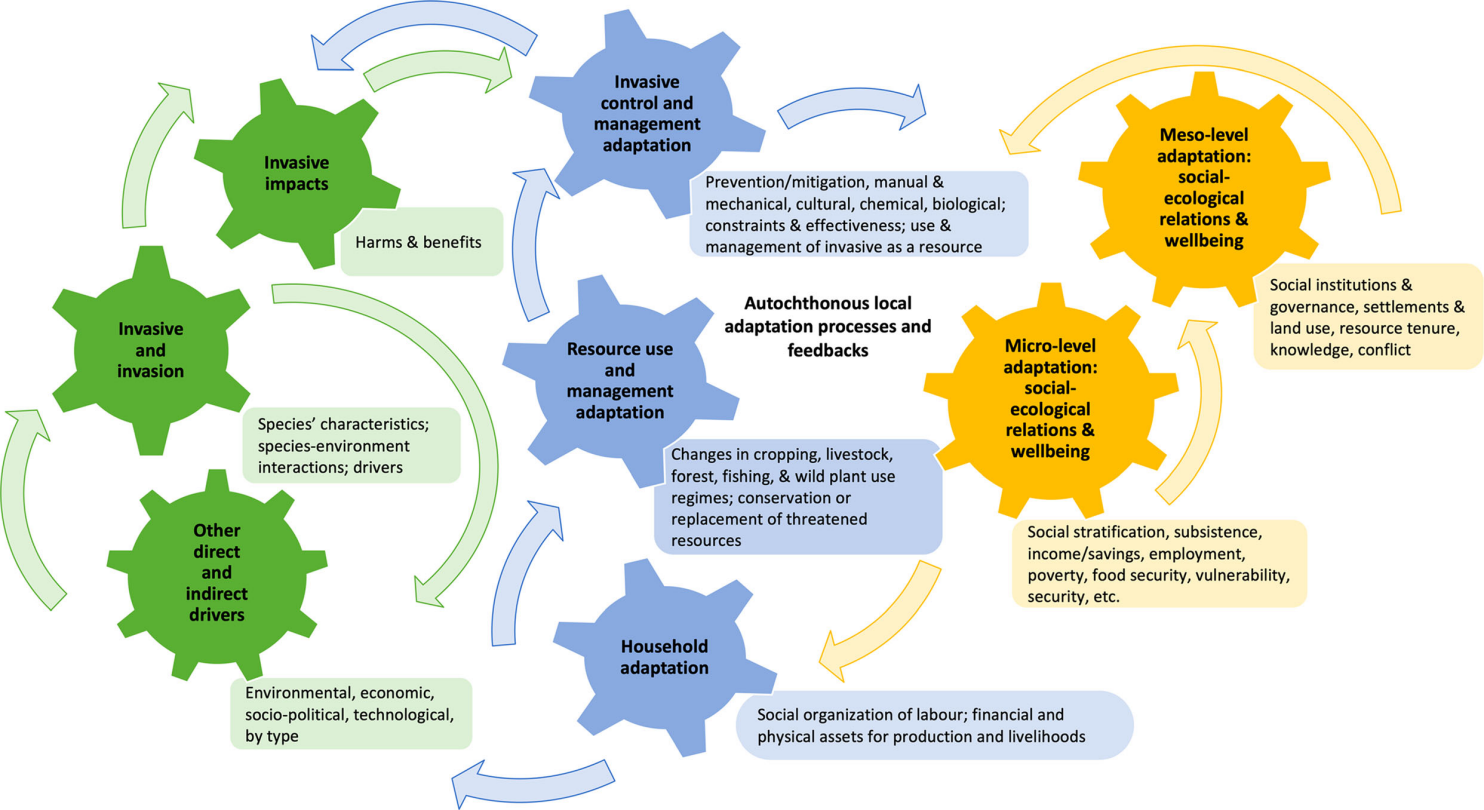
Component 1. Human adaptation to invasive species conceptual framework. Drivers and spheres. Concepts emerged from the metasynthesis process. Green represents invasion and adaptation drivers, blue represents autochthonous adaptations to invasions, orange represents change in social relations and well-being that result from the invasion, other drivers, adaptation and feedbacks for human well-being. For categories and definitions, see Tables S4–S10

**Fig. 2:**
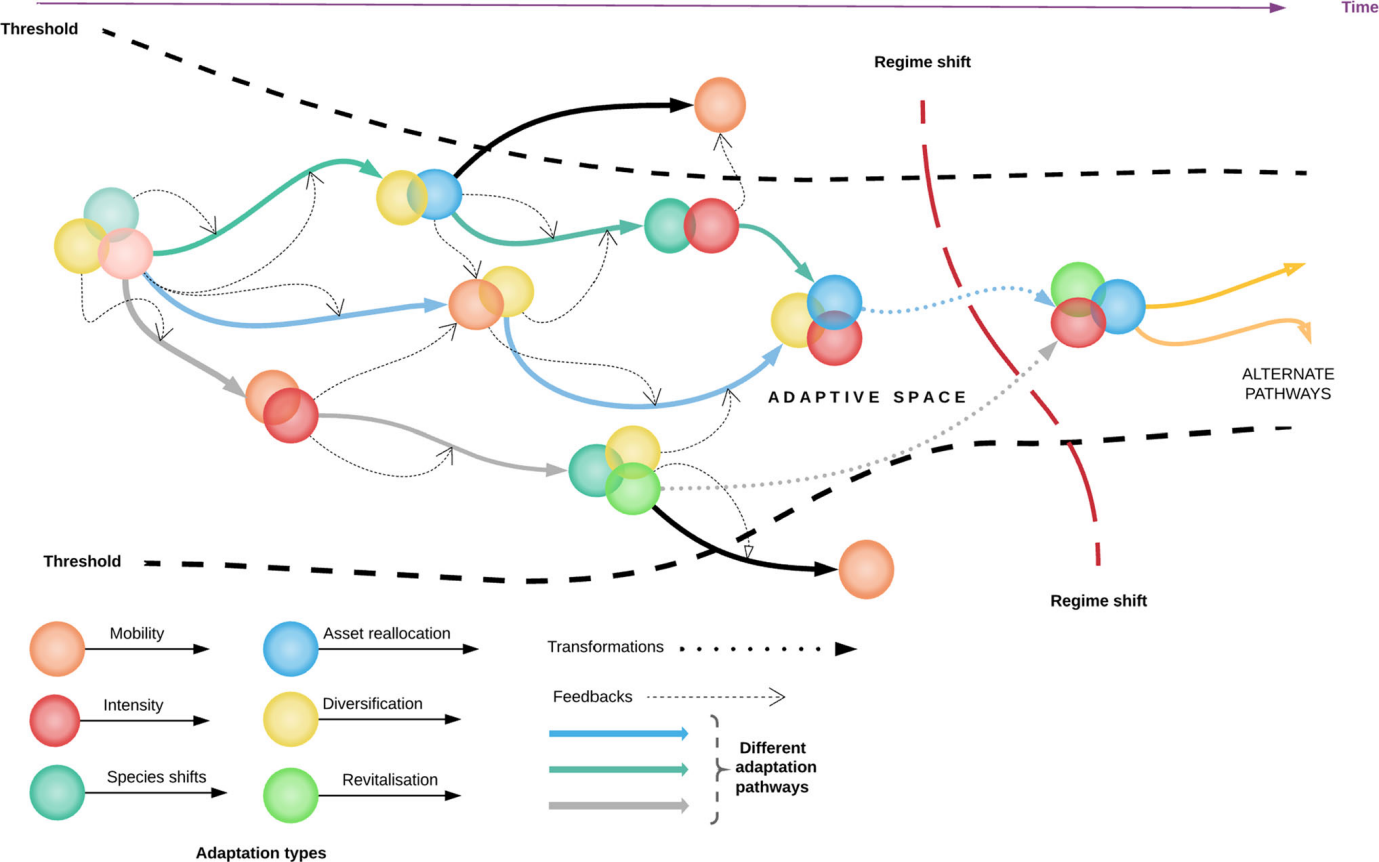
Component 2. Human adaptation to invasive species conceptual framework. Adaptation types, pathways, feedbacks and outcomes. Concepts emerged from the metasynthesis process and literature references in the text. Pathways consist of combinations of adaptation types with distinct socio-ecological feedbacks that affect pathway choices over time. Crossing thresholds shifts social-ecological systems to alternate states (regime shift). Mobility can shift people to different systems. For categories and definitions, see Table S11

A single conceptual framework was developed but, to achieve greater clarity, it is presented as two components. The first (Fig. 1) captures invasion and adaptation drivers and interlocking adaptation spheres. These include invasive control and management, household-level adaptation, resource system-level adaptation and micro and meso-level adaptation. The second (Fig. 2) captures interactions and feedbacks across these spheres in space and time, including types of adaptation, adaptation pathways and social-ecological system outcomes. A fuller understanding of autochthonous adaptation to invasive species must inter-relate these two components. All categories and sub-categories and their definitions are presented in Tables S4–S11. Definitions were derived from those of UN agencies, other relevant global documents and specific scientific disciplines, but many were developed or altered by the author considering case study content. With a few exceptions, sources of definitions were too numerous to cite. A case study example is provided to illustrate each category.

#### Component 1: Drivers and spheres of adaptation

Invasions are provoked by a combination of social and ecological drivers, and in turn drive rapid biodiversity change that can have impacts for human well-being. These impacts drive human adaptation within various spheres of activity that involve different forms of social organisation and scales.

The green ‘wheels’ represent drivers of invasion *and* human adaptation. ‘Invasive and invasion’ captures invasions as ‘direct’ drivers, including characteristics that can make a species invasive, and that can make local environments susceptible to invasion (‘invasibility’). ‘Other direct and indirect drivers’ refers to environmental, economic, socio-political and technological forces and pressures that contribute to an invasive’s spread, determine to an extent its impacts, and shape human adaptation pathways and feedbacks. ‘Invasive impacts’ captures invasion harms and benefits for local populations, which can drive human adaptation as people seek to alter these impacts.

The blue wheels refer to adaptations to invasions separated into three spheres of individual, household or collective activity related with production systems and the enactment of daily life. ‘Invasion control, use and management’ refers to actions directly related to invasions within production systems or at landscape scale. ‘Resource use and management’ refers to adaptation beyond invasive management. Invasions combined with other drivers often affect the environments in which humans enact their daily lives (sensu Ingold 2000)—their living spaces, production systems and ‘wild resource’ environments—to which people respond by altering management of such resource systems, or their demand for and use of such resources (their dependence, or vulnerability). ‘Household adaptation’ refers to the mobilisation and re-organisation of household relations and assets related to invasive management, or to changing resource use and management.

The orange wheels represent adaptation and change at micro- and meso-levels that originate in adaptations in other (blue) spheres, and that in turn feed back into these (blue) spheres as multi-scale interactions that affect human well-being and ecosystem services at higher scales (social-ecological outcomes). ‘Micro’ refers to individuals, households and other small social groups, while ‘meso‘ refers to communities and higher-level social groups (e.g. ethnic, religious, caste or class-based) and institutions. In the framework, as in the case studies, macro-level change phenomena are categorised mainly as exogeneous drivers which, taken together, begin to illustrate the multi-scalar interactions that can strongly influence autochthonous adaptation and social-ecological system change.

#### Component 2: Adaptation types, pathways and outcomes

The analytical categories in this framework include adaptation types, pathways and feedbacks, which then are related, as in Shackleton et al. (2018), to social-ecological system outcomes, including resilience and regime shifts. Figure 2 provides a highly abstract representation of these concepts and interactions. Definitions and case study examples are presented in Table S11.

Concerned that adaptation classifications (as in IPCC 2007, above) are poorly defined and unrelated to the types of risks associated with environmental change and how these affect livelihoods, Agrawal (2010) theorised five different adaptation ‘types’ based on ‘four forms of climate risks.’ These include mobility (distributing risk in space), storage (in time), diversification (across asset classes), communal pooling (across households) and market exchange (purchase and sale of risks). Moving beyond an economic risk perspective, Thornton and Manasfi (2010) altered these types and definitions. Their categories (mobility, exchange, rationing, pooling, diversification, intensification, innovation and revitalisation) were then altered using the methods described above. The resulting typology reflects specifics of adaptation to change in biodiversity and related ecosystem services. Some sub-types were added: ‘mobility’ was disaggregated into resource tracking, migration, resettlement and sedentarisation; ‘diversification’ was sub-divided into ecological, subsistence, wage and enterprises; ‘revitalisation’ was sub-categorised as governance/cultural, conservation and restoration, which distinguishes between adaptations related with institutions and those related with ecological niches and biological resources. Other adaptation types were redefined. ‘Pooling’ became ‘asset reallocation’ to accommodate not only pooling, but as well individualisation (e.g. privatisation). ‘Intensification’ became ‘resource use intensity’ to accommodate both intensification and disintensification and the fact that ‘both’ may be pursued simultaneously. A new adaptation type was added—‘species shifts’—a very important category for examining adaptation to biodiversity change, which essentially refers to changes humans deliberately make in species and species’ communities. Species shifts are nevertheless usually related with other adaptation types (e.g. diversification, intensification, resource tracking, etc.). Results are summarised in Table 5.

‘Adaptation pathways’ considers two fundamental phenomena simultaneously. The first is that adaptation is a set of dynamic, non-linear processes (e.g. a combination of adaptation types) occurring over space and time. Responses that may initiate as ‘coping strategies’ to address very specific stressors can create feedbacks that generate further adaptation (Ellen 2011; Burnham and Ma 2018). For example, some invasive management methods cause invasive spread that is only perceptible after a substantial lag time (Suding et al. 2004); as well, invasive removal can lead to secondary invasions (Pearson et al. 2016), thus generating greater long-term change. To be interpreted, adaptation must be contextualised within longer ‘social-ecological timelines’ that capture adaptation pathways and feedbacks, especially given that current pathways are influenced by historical pathways (Brattland et al. 2019; Thornton et al. 2019).

Second, pathways emphasise the social dimensions of adaptation, which ‘is not separable from the cultural, political, economic, environmental and developmental contexts in which it occurs,’ and where environmental change is only one type of change to which people must adapt (Wise et al. 2014, p. 332). Pathways are ‘governed not only by the nature of an adaptation strategy itself, but also by the diversity of household capacities, the complex and shifting contexts, and the interactions and feedbacks that occur as a strategy is pursued over time’ (Volpato and King 2019, pp. 849–850). Adaptation pathways and determinants (or, at minimum, the main differences between social groups in terms of impacts and responses) that were specified in the case studies are presented in Table 3.

**Table 3:** Examples of adaptation pathways and main determinants

References	Invasive	Differences in adaptation pathways	Main factors affecting pathways			
			Access	Assets	Severity^a^	Other
Hall (2009)	Cattail	Users who either harvest the invasive or the native species manage them to encourage the preferred species; may be in competition. Access to lakes and equipment determine who can harvest what; harvesting declining due to low status and competition with other activities	X	X		X
Aslan et al. (2009) and Eagle et al. (2007)	Yellow starthistle	Lack of coordination between ranchers; ranchers differ in response times; many responses are short-term, leading to spread, increasing control cost and damage. 38% lack time, 46% lack money; landscape heterogeneity affects methods, efficacy; many work off-farm to compensate; those with higher yield losses buy forage and sell livestock; some lease grazing land	X	X	X	X
Johnson et al. (2011)	Medusahead	Ranchers with greater invasion attempt control, are more likely to report marginal effectiveness and return on investment with control and more likely to say they will control in future			X	
Shackleton et al. (2017a)	Erect prickly pear	Most onerous for women who must travel further to collect water, can’t sell livestock products, do most control work				X
Siges et al. (2005)	*Piper aduncum*	Older people lament loss of culturally important species; most have put *Piper* to multiple uses; women benefit as can now engage in agriculture and commercial activities				X
Pandey (2017)	Many invasives	Impacts on resource use systems depends mainly on households’ ability to use assets to control negative impacts; households lack most resources to respond. Poor unable to cope; 10% emigrate, 9% work nationally for wages, leading to further land abandonment and invasion	X	X		
Sullivan et al. (2017)	*Mikania micrantha*	Participation in invasive removal related to differential dependence on community forest resources, perceptions of invasion, neighbourhood size, being a Community Forest member, distance to forest, farming as an occupation and off-farm work	X	X		X
Shackleton and Gambiza (2008)	*Euryops floribundus*	Male livestock owners want to control invasive and have greater power; number of households relying on invasive for fuel is much greater than those with livestock, especially women and the poor, who are harmed by controls, but some benefit from wage labour for control	X	X		X
Burkard (2005)	*Imperata*	Some recover *Imperata* plots. Some abandon dryland plots, as 43% lack capital/labour; 29% lack time to cultivate; 27% prefer to manage wet rice plots. Many don’t plant perennials as this reduces subsistence security and investment costs are high	X	X		X
Keoboualapha et al. (2013)	*Imperata*	Land use intensification through diversification of swiddens (planting perennials) strongly correlates with degree of invasion. Farmers with very low rice yields (due to invasion) start rearing livestock, new enterprises	X	X	X	
Bagnall-Oakeley et al. (1996)	*Imperata*, smallholder rubber	High labour demands are a major constraint so many clear forests. Projects with intensive systems provide better returns on investment but returns are delayed; many become indebted due to high input cost. Jungle rubber producers prevent invasion through long-fallow management	X	X		X
Chikoye et al. (2000)	*Imperata*	Most can’t develop sustainable management strategies due to lack of capital (63%), management options (14%), labour (8%), equipment (7%), herbicides (2%), health (7%)		X		X
Dove (1986)	*Imperata, Chromolaeana,* Bajanarese	Those with more capital cultivate close to the village using short *Imperata* fallows; land use is intensive, requiring wage labour and *C. odorata* is seen as a weed. Those without capital and family labour cultivate further away, using *C. odorata* to improve fallows; *Imperata* is seen as a weed	X	X		
Schneider and Geoghegan (2006)	Bracken fern	Those with more land and off-farm income have less incentive to control; those with less land invest more in control and use income to access better plots; land abandonment higher with greater invasion, and more land or income. Severity of invasion also affects ability/willingness to control	X	X	X	
Shackleton et al. (2015)	*Prosopis* spp.	Conflict around benefits and harms led to end of control programme. Famers most affected in high invasion areas; some sell *Prosopis* wood to offset control costs. Other groups benefit from invasive uses	X	X	X	X
Kent and Dorward (2014)	*Lantana camara*	Soliga have fewer cattle and less savings than Lingayat. Many take on wage labour and abandon agriculture/reduce cattle herds. Lingayat reduce herds, but combine wage labour with agriculture. Soliga depend more on basket making. Lingayat – people earn in part to pay debts; women pay through NTFP collection. Decline in NTFPs affects women more as don’t migrate for wage labour	X	X		X
Mwangi and Swallow (2008) and Becker et al. (2016)	*Prosopis juliflora*	Il Chamus herders more challenged by invasive as lost most herds and grazing land, access to water; displaced from homes & farms, sought pasture in other areas where enter into conflict. Competition with Pokot herders. Some sedentarised taking up agriculture, but subject to invasion; those who cannot pay for labour to control must abandon land. Some took up wage labour, honey and charcoal production from *Prosopis* but doesn’t compensate herd loss. Many switched from cattle to goats	X	X		X
Österle (2008) and Vehrs (2016)	*Acacia* bush encroachment	Major conflicts over grazing land; bush encroachment forced pastoralists to track resources. Pokot forced to abandon plains and migrate to ‘core’ Pokot area. Most sedentarised, taking up agriculture and switching from browsers to grazers. For majority, herding still viable; but cattle pastoralists must travel further to graze and are still subject to conflict	X	X		X

Only case studies with sufficient information to begin to differentiate between social groups’ responses were used to create the table

^a^Severity of invasion. For full references, see Table S3

Adaptation is also often judged to be successful, unsuccessful or maladaptive, but this depends on scale and time—what may be adaptive at one scale or period in time may be maladaptive at another, and what is adaptive for one group of people may be maladaptive for another. Here, the concept of resilience is preferred—it refers to the amount of disturbance that a system can absorb before its key ‘control variables’ shift to an alternative stable state, or regime (Holling and Gunderson 2002; Folke et al. 2010).

**Fig. 3:**
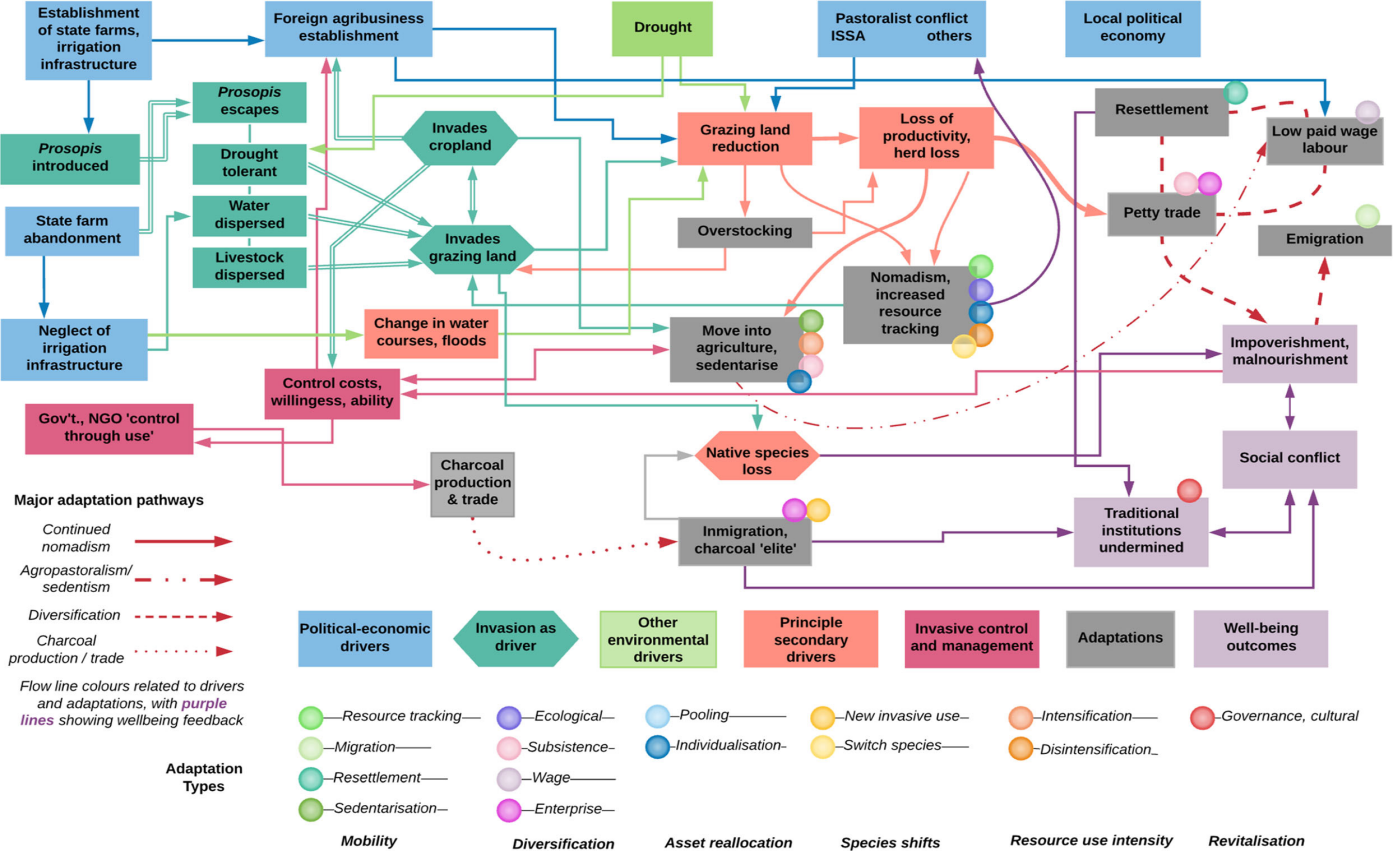
Drivers, adaptation types, pathways, feedbacks and outcomes associated with *Prosopis juliflora* invasion, Middle Awash Basin, Afar, Ethiopia. Own analysis based on case study sources listed in Table S3. Definitions are in Table S11. For adaptation pathways, see especially Müller-Mahn et al. (2010)

Feedbacks are integral to all systems. Negative feedbacks reduce fluctuations and stabilise important drivers, while positive feedbacks amplify processes that destabilise systems (see e.g. Nyström et al. 2012). ‘Human actions affect feedbacks and drivers…which may lead to regime shifts’ when ‘social and ecological feedbacks mutually reinforce each other and maintain or push a social-ecological system towards an undesirable state’ (Stockholm Resilience Centre 2016, p. 1). Causal loop diagrammes are used to capture destabilising effects (Hänke et al. 2017; Shackleton et al. 2018) as in Fig. 3, which presents the case of *Prosopis* invasion in Afar, Ethiopia. However, adaptation can also enhance ecosystem services and human well-being, avoiding system instability or enhancing resilience. For example, humans can break biotic feedbacks driven by invasive species by suppressing processes that favour invasives, or restoring processes favouring desirable species (Suding et al. 2004), as shown in the section on invasive control and management, below. These actions may lead to resilience ‘renewal’. Human adaptation can also lead to regime shifts that enhance ecosystems and human well-being with feedback loops across large areas (Fedele et al. 2018). Breaking feedbacks that maintain social-ecological systems in undesirable states is referred to as ‘transformation’ (Moore et al. 2014), which is defined here as ‘the creation of a fundamentally new system (with a change in state variables) when the existing system is untenable.’ While few case studies presented evidence of adaptation outcomes beyond very small areas or ‘spheres’, those outcomes that could be inferred from the case studies are presented in Table 4. While some of the information in Table 4 is documented, overall outcomes must be considered as tentative and largely hypothetical. With one exception (Anderson and Bollig 2016), none of the case studies addressed social-ecological outcomes directly and, in the majority of cases, only a single component of a social-ecological system (such as agriculture) was considered.

**Table 4:** Examples of case study social-ecological system resilience and shifts with human adaptation to invasive species, real and potential

References	Invasive	Feedback loop (real or potential)	Resilience maintained		Resilience renewed		Instability increased		Regime shift		Transformation	
			EC	WB	EC	WB	EC	WB	EC	WB	EC	WB
Horgan et al. (2014)	Golden apple snail	Pesticides cause collateral damage and harm native predators, possibly increasing invasion					−	−				
Aslan et al. (2009)	Yellow starthistle	Strong lack of coordination between ranchers, lag time in response, short-term responses, lead to further spread, increasing control costs that reduce returns, leading to further spread (documented), conversion of land to agriculture or development (authors’ speculation), which may stop spread (hypothetical)							**–**	**–**		
Tassin et al. (2012)	Australian acacia	Management of *acacia* in fallows increases biodiversity, useful species, income and well-being			+	+						
Siges et al. (2005)	*Piper aduncum*	Management and use of *Piper* increases ecosystem services and human well-being			+	+						
Pandey (2017)	Many invasives	Climate change and other pressures lead to large-scale land abandonment allowing further invasion; increased costs and reduced output leaves farming in deficit; outmigration and abandonment lead to further invasion							**–**	**–**		
Nepal case cluster	*Mikania micrantha*	Dung-based bio-gas stoves introduced to decrease fuelwood demand increased demand for grass to feed livestock. *Mikania* decreased grass resources, so people travelled further to collect them, further spreading *Mikania.* Decreasing fuelwood and grass resources increases dependence on bio-gas					−	−				
Roder et al. (1995a, b)	*Chromolaena*	Land pressure and population growth led to dramatically shortened fallows, degrading soils and allowing invasion. *C. odorata* used to stabilise fallow systems and yields			+	+						
Shackleton and Gambiza (2008)	*Euryops floribundus*	A control project cleared areas, increasing grazing capacity, which might increase stocking rates. Heavy grazing spreads invasive, leading to need for control. Invaded areas have higher species richness; control reduces resources for the poor, increasing resource pressure							**–**	**–**		
Bagnall-Oakeley et al. (1996)	*Imperata*	*Imperata* is controlled preventatively in jungle rubber systems with greater biodiversity and valuable trees	+	+								
Jagoret et al. (2012)	*Imperata*	Transformation of grass/cropland to cacao agroforestry overcame *Imperata*, water and soil fertility deficits, and increased tree diversity (67 species; 24% native); yields similar to those in other forest areas. Land was privatised, could lead to inequality and food crop deficits									+	+ (-?)
Martin (2014)	Water hyacinth	Hyacinth used as substrate in unique floating garden system, controlling spread. Intensification caused eutrophication, increasing invasion and reducing fish. In part because of this unique system, international designations brought many tourists, contributing to pollution							–	–		
McWilliam (2000)	*Chromolaena*	Invaded already degraded rangelands; grazing pressure led to abandonment; livestock moved to marginal lands and forests. Uncontrolled burning and grazing contributed to degradation across highlands. High dependence on cattle as wealth store and for exchange (author); degradation and negative well-being impacts likely to continue (hypothetical)							–	–		
Österle (2008); Vehrs (2016)	*Acacia* bush encroachment	Major shift from browsers to grazers herded near homesteads; browsers produced for meat. Allowed sedentarisation and farming. For most, herding still viable. For many, honey production provides income equal to livestock. Farming, honey production promoted by outsiders but spread on their own. Bush encroachment turned from negative to positive. But when cattle grazed further away, provokes conflict, which is ongoing									+	±

Only case studies with sufficient information were used to create the table. For full references, see Table S3

*EC* ecosystems, *WB* well-being

### Invasion and adaptation drivers

The ‘drivers’ concept is widely used to analyse causality in environmental change (Chaffin et al. 2016). ‘Direct drivers’ are physical or biological processes that have an ‘unequivocal’ influence on ecosystem processes, while ‘indirect’ drivers act ‘more diffusely’ to alter direct drivers—anthropogenic drivers are all considered as indirect. Environmental change entails multiple interacting drivers operating at different geographical and temporal scales (Nelson et al. 2005). In complex social-ecological systems, causal relations also tend to be highly context-dependent (Magliocca et al. 2018), which some invasion biologists argue is also the case with invasions and their impacts (Hulme et al. 2013; Bellard and Jeschke 2016).

For many invasion scientists, however, there is only one green ‘wheel’ (Fig. 1): the invasive species, together with the invasibility of the local environment. Shackleton et al.’s (2018) analysis of invasion-related regime shifts goes beyond this (see also Gaertner et al. 2014). It refers to social and ecological drivers and interactions as invasion drivers, and also to social-ecological feedbacks. However, three crucial processes are missing in their analysis. First, *invasions are only direct drivers of social change and adaptation when humans experience them as such*; people may not experience direct biophysical impacts or consider them as significant for well-being (of themselves, or of other significant species or ecosystems). In one of the metasynthesis case studies, for example, although the invasive had significant negative impacts, it had been around for so long that people assumed it was a natural part of the environment—only a minority thought that it might be subject to management (Berget et al. 2015). Second, *many of the drivers of invasion are, at the same time, drivers of human adaptation to invasives*. For example, in the Afar region of Ethiopia (Fig. 3), the establishment of State Farms on pastoralists’ grazing land drove both *Prosopis* invasion and pastoralists’ responses, including overgrazing of remaining grazing land, and movement of herds to new grazing areas. Third, *human adaptation to invasives itself becomes a driver of further social and ecological change and adaptation*, as it creates feedbacks that affect resilience or produce regime shifts. A regime shift has certainly occurred in the social-ecological system in Afar, where it is the interactions between the invasion and other drivers that explains ‘why pastoralists experience the impacts of *P. juliflora* in the manner they do’ and why and how they adapt to it (Rogers et al. 2017, pp. 8–9) (see below, and Fig. 3). In distinction to the studies on the Afar, Shackleton et al. (2018) provide little empirical evidence of what they term ‘social regime shifts’ for the case studies they analyse.

#### Framework and case study drivers and feedbacks

Case study researchers identified the drivers, driver interactions and their contextual contingencies involving different scales, agents and actors, and affecting different interlinked social-ecological system components. Due to geographical and temporal limits and research foci, most studies didn’t fully specify drivers and feedbacks but, where there are clusters of in-depth multidisciplinary case studies, these multiplied. Table S4 provides the categories, definitions and single case study examples, while Tables 3, 4 and Fig. 3 show some of the driver interactions that gave rise to invasion and adaptation processes and feedbacks.

Drivers were classed into the following categories: environmental (direct), and economic, socio-political and technological (indirect). Demographic drivers are indirect (the result of other drivers), and ‘technology’ includes both local and scientific knowledge. Notwithstanding the importance of cultural values, beliefs and norms as drivers of human–environment relations (e.g. Robbins 2004; Kull et al. 2011), there was insufficient information in the case studies to merit inclusion of this category. A single driver may be complex and categorised or counted in multiple ways (Anastasopoulou et al. 2009). A total of 89 drivers were found in the case studies, which were often clustered around specific resources (such as forests) or production systems (such as livestock), or were associated with livelihood pressures (e.g. around land or labour access). To better analyse such patterns and trends, drivers were further classified into sub-types related to water, vegetation, soil, land, livestock, forests and trees, livelihoods and invasive use; for sake of brevity, however, these categories are not systematically referred to below (but see Tables S4 and S5).

All case studies identified invasives as direct drivers of change and usually also specified those characteristics of the invasive and their associations with invasibility and spread in the local context. For example, in Afar, Ethiopia (Fig. 3), *Prosopis juliflora* is drought tolerant and dispersed by water and livestock; it outcompetes many native species, such as grasses. It also forms dense thickets that harbour predators and impede mobility. It has strong thorns that prevent predation and cause injury.

Other direct and indirect drivers are not directly associated with invasions, but are associated with broader change processes that create conditions allowing invasions (invasibility) and for specific adaptation responses. Table S5 provides the frequencies of mention of these drivers by categories and sub-types. For example, one ‘forest/tree related economic driver’ of invasion was deforestation for commercial agriculture, plantations, ranching, mining or logging, cited in four case studies (14% of the total).

All cases cited environmental drivers, for a total of 18 distinct drivers mentioned. Drivers were at times classed solely as environmental because no indirect drivers were specified, e.g. in the case of ‘disturbance,’ or where ‘livestock overgrazing’ led to invasion. Several, however, are also classed as economic, technological or socio-political (see Tables S4 and 4, McWilliam 2000; Pandey 2017). Economic drivers are market-related, that is, influenced by price signals; over half of the cases cited one or more of the 23 economic drivers. For example, both crop damage and profit motives were important in Cameroon farmers’ decision to convert *Imperata cylindrica*-dominated grasslands to cocoa plantations in a region that experts consider unsuitable for cocoa (Jagoret et al. 2012). Socio-political drivers (27 in total) were the most frequently cited, and are defined as non-market forces influencing decision-making, including phenomena such as subsistence goals, cultural values, governance and social and political power. In Mexico, for example, government road construction promoting colonisation led to immigration, large-scale deforestation and land use intensification, driving bracken fern invasion (Douterlungne et al. 2010). Many socio-political drivers provide evidence of cross-scale interactions, as in Fig. 3 above. A total of 33 technological drivers were cited, referring to the introduction of locally new technologies or the substitution or extension of existing technologies including scientific or local knowledge, which induce change or condition adaptation. For example, the Lacandon Maya’s indigenous knowledge of vegetation management and its deployment against a new invasive species allowed them to maintain the resilience of long-fallow swidden systems (Douterlungne et al. 2010).

Figure 3 illustrates the drivers of invasion and adaptation in Afar, Ethiopia, where around 80 000 pastoralists traditionally used the Awash river’s wetlands to graze cattle in the wet season, while grazing cattle up to 150 km away in the dry season. From 1940 to 1980, Somali Issa pastoralists moved into these dry season areas, generating violent conflict with the Afar and expelling them. This conflict was a higher scale socio-political driver, ‘fueled by geopolitical interests, ideologies and military support of Somali-governed states’ (Müller-Mahn and Rettberg 2012, p. 304). As well, the Ethiopian state established cotton farms and irrigation infrastructure (economic-socio-political-technological driver) on pastoralists’ wet season grazing land, introducing *Prosopis*^9^ to these farms to improve soils and provide wind breaks and shade (environmental-socio-political). State farms were abandoned when the government regime collapsed, allowing *Prosopis* to escape (socio-political). Subsequent governments failed to maintain hydraulic infrastructure, leading to river diversion and flood water irregularity (socio-political-technological-environmental); as *Prosopis* is easily water dispersed, it spread throughout pastoralists’ wet season grazing areas (Rettberg 2014). When drought occurred in 2002–2003, because of *Prosopis*, which is drought tolerant and outcompetes grasses, pastoralists were unable to recover herds (Rettberg 2010). *Prosopis* is spread by livestock, so most grazing lands were invaded. Unable to migrate seasonally to allow grasslands to recover (socio-political-environmental), remaining pastures were overgrazed (socio-political-environmental), further spreading *Prosopis* while reducing herds (Mehari 2015).

#### Invasive impacts as drivers

Invasive impacts are those that humans perceive as harmful or beneficial for themselves and their environments. In invasion biology, ‘impacts’ are generally ill-defined (understandably, given the above) and, until recently, were limited to harmful effects, ecological changes and small geographical scales (Jeschke et al. 2014) (see also Vilà et al. 2011; Hulme et al. 2013). Here, harmful impacts are those that reduce provisioning, regulating, supporting, and cultural goods and services and/or increase costs to achieve these, or increase human morbidity and mortality. *Beneficial impacts* are those that increase goods and services, reduce costs to achieve these or enhance human health (Table S6 provides definitions and examples). As is to be expected in HAIS case studies, 90% reported harmful impacts and over a third reported only harms—most studies reported adaptations that mitigated harms or turned harms to benefit. The most commonly mentioned harms were reductions in crop yields, grazing land potential and useful native species, and increases in labour requirements. Nearly two-thirds of the case studies reported beneficial impacts and a tenth were solely beneficial, but more than half reported both harms and benefits.

Several studies argued that benefits outweigh harms because people have managed harms and created benefits (Dove 1986; Jagoret et al. 2012; Tassin et al. 2012; Martin 2014; Berget et al. 2015). In part this is unsurprising because, depending on the context, ‘Invasions can have positive as well as negative feedbacks for ecosystem functions’ (Vilà et al. 2011, p. 702). However, in the case studies, such functions were often co-produced (Palomo et al. 2016), that is, people altered feedbacks through management. Table 4 shows several examples where such efforts renewed resilience (Roder et al. 1995a; Siges et al. 2005; Tassin et al. 2012). However, the capacity to mitigate harms and derive benefits was unequally distributed, as shown in Table 3, on adaptation pathways.

### Adaptations: Autochthonous invasive control, use and management (ICM)

ICM adaptations are people’s actions to directly control, use and manage invasive species. In cases where a single invasive generates only negative impacts, it is possible for these to be mitigated to ‘acceptable’ levels of harm by implementing existing ICM methods without further adaptation. However, when ICM alone requires deployment of substantial assets (e.g. labour and other resources), this can generate feedbacks for livelihoods and well-being that provoke further adaptation. In some cases, invasions and other drivers lead to regime shifts that then may become ecologically resilient, so systems cannot be returned to previous states simply by applying existing control methods (Suding et al. 2004) (see Table 4, McWilliam 2000; Shackleton and Gambiza 2008). In three of the reviewed cases, invasive harms were prevented with little diversion of resources, thus averting the need to adapt.^10^ In several, species shifts (Table 5) were evident: invasives were managed to provide substitutes for valuable species lost due to invasion or to renew system resilience (see below and Table 4).

Invasive scientists have always strongly promoted efforts to alter negative impacts through invasive species prevention, eradication or control, but such efforts are not considered as ‘adaptation’. Here, ‘invasive control and management’ (ICM) includes not only attempts to prevent or control the growth and propagation of invasive organisms (e.g. Tu et al. 2001; Clout and Williams 2009) but also to manage them to beneficial effect. The category ‘Invasive use and management as a resource’ captures the fact that people might use invasives as a resource, *not* manage or control beneficial invasives, or encourage their persistence or spread. ICM is further classified into ‘standard’ second-tier types: preventative, manual, cultural, chemical and biological, each with third-tier categories referring to different associated methods.

Two additional second-tier categories are associated with ICM: effectiveness and constraints. Effectiveness is the degree to which the methods used reduce invasive populations to below levels of unacceptable damage (or, where managed for benefits, achieve these); constraints are limits on people’s ability to achieve effectiveness. Assertions about effectiveness in the case studies were based on data ranging from experimental field trials (e.g. Roder et al. 1995a, b, 2001; Awanyo 2007) to informant statements, to judgements that production systems were performing well (e.g. Dove 1986). Table S7 provides definitions and examples.

Autochthonous ICM is significant in the absence of external control efforts *and* in their presence. External interventions were evident in only 15% of the case studies or clusters; in the rest, local people managed invasives alone. External controls were counter-productive in two cases (Table 4, Shackleton and Gambiza 2008, and Fig. 3), supportive but relatively unsuccessful in four (Eagle et al. 2007; Aslan et al. 2009; Johnson et al. 2011; Horgan et al. 2014), and suspended due to conflict in two (Müller-Mahn and Rettberg 2012; Shackleton et al. 2015).

ICM can be a complex adaptation, depending upon whether the priority is simply to reduce populations of invasive species through direct removal, or to change the environmental conditions and feedbacks that lead to invasive spread and impacts, towards ecosystem restoration (Suding et al. 2004). Most studies reported ICM methods across study populations rather than use by individuals—thus, the number of methods was likely under-reported. On average, four methods were reported per case; several reported seven or more. Most ICM appeared to be aimed at restoration—decreasing instability and promoting resilience—which requires a combination of methods such as restoring physical conditions, removing specific species (e.g. weeding), altering nutrient regimes (e.g. mulching), using fire, excluding or reintroducing herbivores (grazing) and adding species (e.g. establishing canopies to alter sunlight). All studies reported use of manual or mechanical methods, while a combination of manual and cultural methods, together with burning, were reported in slightly over half.

While some studies reported use of mechanical methods (e.g. tractor tillage, mowing), most controls were performed manually using rudimentary tools, which are mainly used to control small invasive populations or small invaded areas. Manual methods were also used to promote invasion, e.g. selective weeding to encourage invasive presence in fallows (Dove 1986; Roder et al. 1995a; Awanyo 2007; Tassin et al. 2012). When effectiveness was reported, manual and mechanical controls were effective in about half of the cases; in some, they could lead to undesired invasive spread (Aslan et al. 2009; Sullivan et al. 2017). Constraints were especially related to high labour demand and cost of wage labour and equipment.

The term ‘cultural control’ is used to categorise methods that modify environmental conditions, in effect, to disrupt the internal feedbacks that ‘constrain restoration of the degraded state…in ways that will facilitate the transition to the desired state’ (Suding et al. 2004, p. 50). They are aimed at ecosystem management, which is generally more scale-neutral compared with manual methods. In the studies, cultural controls were always combined with other ICM methods, which depend on substantial local ecological knowledge and increase adaptation complexity (Bastiaans et al. 2008). Indigenous Chinantec swidden farmers in Oaxaca, Mexico, successfully restored unproductive bracken fern-invaded land by clearing bracken, planting and weeding crops, caring for saplings that sprouted and planting perennials to shade out bracken and promote natural succession (Berget et al. 2015). As cultural controls serve to at least partially restore systems, they did not cause invasive spread and were the most effective of all reported methods, but labour demand, limited land access and low yields were mentioned as constraints.

If used appropriately and in combination with other methods, prescribed burning can be used toward restoration. It is commonly used against invasives that are not fire-resistant, but it can encourage those that are fire-adapted. It was used to clear land (which may control invasives) and for direct invasive control or to restore resources such as forage grasses threatened by invasion. Many cases reported that burning was effective but, in several, burning might have increased spread (Schneider and Geoghegan 2006; Aslan et al. 2009; Keoboualapha et al. 2013; Murphy et al. 2013). Burning was also preferred even when it was less effective, as it requires little labour and few inputs and can be used on a larger scale (Chikoye et al. 2000; Aslan et al. 2009). A few cited fire damage and burning prohibitions as constraints.

Chemical controls were cited in a third of cases, where most reported use by a small minority of informants who could afford them. Cost was not the only constraint—chemicals were also used far less than other methods due to collateral damage (e.g. to wildlife, crops, health). Chemical use can create greater ecosystem instability (e.g. Table 4, Horgan et al. 2014). Biological control refers to use of natural enemies (insects, predators or pathogens) and is often proposed as the most efficacious and cost-effective type of control, but non-target damage is a concern. Only native biological control agents were used in the case studies; in Asia, ducks were used to control golden apple snails (Halwart 1994) and plants were used to lure snails to areas where they were harvested or eaten by leeches (Joshi et al. 2001; Tsai et al. 2016). These controls were effective, but constraints included collateral damage and chemical defeat of these agents.

Invasive use (for goods and raw materials) is considered as a control option where other methods have failed or where use might keep invasive populations in check (Nuñez et al. 2012; Barnes et al. 2014). Table S7 presents definitions and examples (see also Table 3). In a large majority of the cases, people used the invasive for subsistence and, in some, for income and for new activities (diversification). For example, in Lake Inle, Myanmar, water hyacinth is used and sold as a substrate for a unique indigenous hydroponic agricultural system on the lake (Martin 2014). Use may involve management of invasives to ensure the required abundance for specific uses over time. While much invasive case study research refers to invasive use (see Shackleton et al. 2007, 2019b), a much smaller fraction refers to *management* for use. Management for use was found in a third of all cases reporting use—in most, controls were not implemented, but in half, invasive growth or spread was encouraged.

### Adaptations beyond invasives: Resource use and management systems

Given that invasives can alter feedbacks that lead to regime shift, and that ICM often involves alteration of these feedbacks, adaptation often entails changes in the use and management of a wider range of landscapes and wild and domesticated resources. The intention or effect of such adaptations may be to decrease instability, increase resilience or completely transform social-ecological systems (Table 4). In these processes, people may manage, offset or benefit from invasions and, when this is not feasible, change reliance on specific components, e.g. by switching species, diversifying, intensifying, disintensifying or migrating (Table 5).

Second-tier categories capture adaptations in production and wild resource management systems or abandonment (disintensification) as an adaptation. Two additional categories capture change beyond production systems: conservation of threatened species, and replacement of reduced or missing biological resources (Table S8 gives definitions and examples). In spite of the fact that highly biodiversity-dependent rural populations in developing regions rely on an extensive range of wild and domesticated biodiversity for livelihoods (e.g. Hickey et al. 2016), most of the case studies focused on a single production system, mainly agriculture, so adaptations in other resource use and management systems were probably under-reported.

Almost all cases involved cropping systems and more than half of these were adapted; especially, major changes were made to fallow systems. In some, both cropping and fallow systems were adapted by changing crop species and improving soil (Dove 1986; Burkard 2005; Tassin et al. 2012; Berget et al. 2015). Moving into agriculture was associated with pastoralist sedentarisation (Müller-Mahn and Rettberg 2012; Hamedu 2014; Mehari 2015; Becker et al. 2016; Abdulahi et al. 2017). In a few cases, when ICM demanded much labour and led to declining yields, farmland was abandoned, and new land cleared for agriculture (disintensification/intensification together) (Bagnall-Oakeley et al. 1996; Schneider and Geoghegan 2006).

Because invasives are very difficult to control on a large scale, they often have dramatic effects on grazing resources. Almost all livestock managers adapted livestock systems (see Table 4 and Fig. 3). In several cases, grazing land abandonment led to lack of management, which drove further invasion (McWilliam 2000; Schneider and Geoghegan 2006; Anderson and Bollig 2016; Greiner and Mwaka 2016). A major adaptation that could break feedback loops was to switch from grazers (negatively affected by invasions) to browsers (less or not affected), as in Kenya (see Table 4, Österle 2008; Vehrs 2016) and, to a lesser extent, Ethiopia (Müller-Mahn and Rettberg 2012). Volpato and King (2019) showed that such a process occurs in phases over decades. In other cases, part of the population abandoned livestock altogether, and some pastoralists also broke feedback loops by sedentarising and taking up agriculture (see Table 4, Österle 2008; Vehrs 2016, and Fig. 3). Adaptation also occurred where livestock rearing was supplementary to farming (see Table 3, Kent and Dorward 2014; and Thornton et al. 2019). People also diversified by moving into livestock production, such as in Laos (Keoboualapha et al. 2013) and Java, Indonesia (Dove 1986). *Imperata*, *Prosopis* and *Mikania* were often used as feed resources when native forage was reduced, the invasive provided a reliable supply, and use might have helped to control invasions (Burkard 2005; Siges et al. 2005; Rai and Scarborough 2015).

Nearly half of the cases reported adaptations related to the creation, management, use, conservation or restoration of forests, woodlands, trees and associated wild resource (see e.g. Table 4, Siges et al. 2005; Jagoret et al. 2012; Tassin et al. 2012 and the Nepal case cluster). In Afar, Ethiopia (Fig. 3), tree cutting was traditionally discouraged and charcoal making was punished but, with government and NGO promotion of *Prosopis* charcoal production as ‘control through use,’ cutting became tolerated (Hamedu 2014; Wakie et al. 2016). Some charcoal producers harvested native species with highly negative ecological and social repercussions. Another adaptation pathway that resulted in renewal or transformation was a switch from agriculture to agroforestry (e.g. Table 4, Jagoret et al. 2012; Tassin et al. 2012).

In eight cases, adaptations protected or encouraged a diversity of species with use values that were (or might otherwise be) threatened by invasion. In some, resilience was maintained or restored (e.g. Table 4, Bagnall-Oakeley et al. 1996; Tassin et al. 2012). In Cameroon, farmers transformed *Imperata* invaded cropland into cacao agroforestry systems that contained 67 different tree species, including many native forest trees (Jagoret et al. 2012). No case reported that threatened species were managed in the wild, but one reported that threatened trees were transplanted onto private land (Rai and Scarborough 2015).

When invasions and other drivers reduced access to biological resources, people often substituted these with other local or non-local resources. In some cases, locally available species were used as substitutes, which was possible due to ‘utilitarian redundancy’ (different species provide the same use or function), which depends in part on species richness (Santoro et al. 2015) and in part on rich local ethnobotanical knowledge (Díaz-Reviriego et al. 2016). In many cases, the invasive itself was used as a substitute. The most striking example is from Papua New Guinea, where *Piper aduncum* substituted for most woody forest resources lost due to invasion (Siges et al. 2005).

### Household adaptations

Households are the most ‘basic adaptation unit,’ that is, where most adaptation occurs (Thornton and Manasfi 2010). Household ‘attributes’ constrain or facilitate the pursuit of different adaptation pathways (Wise et al. 2014). As households and their members pursue different pathways (Table 3), different interactions and feedbacks arise (Thornton et al. 2019; Volpato and King 2019) that have different outcomes (Table 4).

The impact of an invasive on crop yields might be simply managed by weeding, but this can lead to a reallocation of household labour or hiring of wage labour, which can reduce the capital available for other investments, change the division of labour and reduce livelihood diversity and flexibility (Komatsu et al. 2018). Increased income from improved invaded fallows or invasive use may divert labour away from subsistence production and increase food purchases, improving or worsening nutrition (Jagoret et al. 2012). Changing well-being can lead to other adaptations, such as moving out of agriculture and into wage labour (diversification), which in turn might increase or decrease overall adaptive capacity and well-being or contribute to invasive spread, since invasives are no longer managed. Households thus must attempt to negotiate the feedbacks of even simple adaptation strategies or change strategies as these feedbacks become apparent. As adaptation pathways unfold, the required critical household attributes may change—for example, from local agroecological knowledge to marketing skills. Because of these feedbacks, ‘theoretically viable policies to stimulate or support adaptation may not result in the anticipated livelihood outcomes’ (Volpato and King 2019, p. 850).

In the framework, household adaptations refer to changes in household labour and capitals either for ICM or for other resource management adaptations. None of the case studies fully reported these feedbacks and subsequent adaptations, and most focused primarily on changes in labour and capital (e.g. credit, savings and technology). Table S9 presents definitions and examples.

Labour demand for ICM had the strongest feedbacks for household adaptation. In most of the cases, households supplied ICM labour and, in some, it substantially increased women’s (Shackleton et al. 2017a) or women’s and children’s workloads (Chikoye et al. 2006). There was little evidence of communal labour, and only minority used wage labour. Labour constraints for ICM were extremely common but, in some cases, there were low or no constraints. When demand was very high, this forced people to either stop ICM or abandon invaded land (Burkard 2005; Schneider and Geoghegan 2006; Mwangi and Swallow 2008; Müller-Mahn and Rettberg 2012; Keoboualapha et al. 2013). Some argued that labour and land access were the most important determinants of how invasions were perceived and managed (Dove 1986; Schneider and Geoghegan 2006). Higher labour demands were, at times, compensated by invasive benefits (Siges et al. 2005; Shackleton et al. 2015). Household labour also affects, and is affected by, resource management adaptations. The need to track dwindling resources had major consequences for non-timber forest product collectors in Nepal, who had to travel much further for lower rewards, forcing many people to shift livelihood strategies or substitute species (Rai and Scarborough 2015; Khadka 2017). However, in Papua New Guinea, agricultural labour demand decreased because *Piper* fallows were easier to clear, which also allowed women to participate in agriculture (Siges et al. 2005).

Financial costs other than wage labour were mainly related to purchases of chemicals and associated ICM equipment. In most cases where these costs were apparent, they were either financially damaging or unaffordable (Eagle et al. 2007; Aslan et al. 2009; Shackleton et al. 2017b). At times, major capital outlays were required to change resource use and management, such as to convert rangeland to agriculture, which is the only means of controlling *Prosopis* in Ethiopia (Mehari 2015).

In addition, resource use and management adaptations change what households invest (inputs) and produce (outputs) (see Table S9 for definitions and examples). Adaptation pathways often involve livelihood diversification through value-added activities (i.e. processing, marketing) and off-farm employment. When outputs (e.g. crop yields) were reduced, households often reallocated resources between these productive activities (e.g. in Table 4, Mwangi and Swallow 2008; Müller-Mahn and Rettberg 2012; Keoboualapha et al. 2013; Kent and Dorward 2014). No study provided a comprehensive assessment of such changes.

Most studies mentioned changes in inputs or outputs related to natural resource-based production systems where, in most cases, outputs declined. Increased outputs were more frequently reported from improved fallow systems. Reduced outputs may lead to shifts to other productive activities which can further reduce inputs and outputs in the invaded systems. In India, *Lantana* negatively affected outputs of cropping, grazing and NTFP collection. Some people adapted by temporarily migrating for wage labour, but the loss of labour due to migration has led some to abandon agricultural land and further reduce cattle herds (Table 4, Kent and Dorward 2014).

### Micro- and meso-level adaptations and change in social-ecological relations and well-being

Change and adaptation in social relations and their consequences for well-being are considered in two additional spheres corresponding to micro- and meso-scales of adaptation. Two questions are relevant here: one is how social relations at micro- and meso-scales affect adaptation pathways (e.g. Crane 2010; Eriksen et al. 2015; Sovacool 2018), and the second is how adaptation pathways affect social-ecological relations and outcomes at these scales. Table S10 provides definitions and examples.

As is also the case in much climate change adaptation research (Burnham and Ma 2018), many studies did not examine such multi-scalar interactions, e.g. between households, micro- and meso-levels. In some cases, however, this was because resilience was maintained (Bagnall-Oakeley et al. 1996; Douterlungne et al. 2010) and did not provoke such changes. Of those cases addressing social relations and well-being, the micro level was absent in 16% and the meso-level was absent in 31%. Two-thirds of the cases reporting nine or more social-ecological relations and well-being topics are case clusters, showing that knowledge of social-ecological relations and dynamics is enhanced when several multidisciplinary studies are carried out. One applied the HABC framework (Kent and Dorward 2014; see also Thornton et al. 2019 this issue). Adaptation to *Prosopis* invasion in Afar, Ethiopia, the most thoroughly documented to date, is used to illustrate how the conceptual framework is applied (Fig. 3).

#### Micro-level

Most cases discussed well-being and social stratification, which is often associated with different adaptation pathways (Table 4). Social stratification refers to how people are positioned in a status hierarchy based on socio-economic conditions or factors such as age, sex, ethnicity, occupation, type of production system (e.g. swidden versus intensive agriculture) or technology used (e.g. use of external inputs or mainly local ecological knowledge). In Afar (Fig. 3), between 1997 and 2001, 36% of cattle and 20% of camels were lost in the most invaded *woreda* (Mehari 2015), and the loss of many native species also had negative impacts for livelihoods and well-being (Wakie et al. 2016). Pastoralists became increasingly differentiated (socially stratified) by adaptation pathways, which were strongly associated with livestock losses. Those with the lowest losses continued nomadic pastoralism and combined this with other livelihood activities. Those who lost fewer livestock sedentarised and moved into agropastoralism, but this pathway required access to labour (especially for *Prosopis* control), credit, knowledge and markets (Müller-Mahn and Rettberg 2012). Poorer agropastoralists had to work for wages, limiting their ability to control *Prosopis* on their farms (Rogers et al. 2017). Those (60%) with the greatest livestock losses moved into activities previously used only in emergencies: > 90% worked on cotton farms, emigrated or sold fuelwood and mats. Poor households living near large farms or urban areas combined sheep and goat sales with up to four non-pastoral activities, most of which were low paid and temporary. Clan leaders, on the other hand, were enriched by leasing land and contracting out labour for *Prosopis* control to foreign agribusinesses. Women’s workloads massively increased and female-headed households became the most destitute (Müller-Mahn and Rettberg 2012).

Personal security also eroded with *Prosopis* invasion and adaptation. Immigrants who came to the area for the charcoal trade violated cultural norms and raped and even murdered pastoralists (Datona 2014; Wakie et al. 2016); in turn, charcoal producers had to be protected as *Prosopis* invasion forced lions and hyenas to move closer to villages, killing livestock and people (Mehari 2015). Health was also affected; *Prosopis* thorns wounded livestock as well as humans’ limbs and eyes, causing blindness, disability and amputations due to infection. Access to healthcare services was diminished as thickets and thorns impeded mobility, and *Prosopis* management costs diverted funds away from healthcare (Wakie et al. 2016).

#### Meso-level

Most cases citing meso-level adaptations referred to social institutions, governance and resource tenure, while some cited adaptations in land use and settlement patterns—the Afar case cluster combined these (Fig. 3). The government (national and local) considered pastoralists to be the source of land degradation and marginalised them in decision-making around several major interventions in the area, and thus failed to take their interests into account. Four adaptation processes transformed the Afar’s institutional capacity to sustainably manage resources, including the invasion: commodification and natural resource privatisation, cultural change (norms and values, communal resource management rules and traditional knowledge), impoverishment, and conflict with outsiders. Social norms of reciprocity and risk sharing became partly monetised, challenging social values and identity and weakening local institutions (the opposite of ‘revitalisation’ in Table 5) (Rettberg 2010; Müller-Mahn and Rettberg 2012). There were also greater pressures for cooperation, as higher demands were placed on increasingly limited resources, strengthening traditional management institutions in some communities (Müller-Mahn and Rettberg 2012). Communal livestock management generally eroded as herders made grazing and pasture management decisions individually. Before the invasion, when households lost livestock, clans divided risks and provided people with means to restock herds, but this became rare since the risks of loss were high due to invasion (Hamedu 2014). The ‘charcoal elite’ gained influence and undermined customary natural resource management institutions (Müller-Mahn and Rettberg 2012; Datona 2014). Invaded grazing land was leased to foreign agribusinesses, which caused pesticide pollution and salinization (Müller-Mahn and Rettberg 2012).

When linked with other drivers that negatively affect adaptive capacity, invasions can contribute to overt and, at times, violent conflict over land, rights to other resources (e.g. NTFPs, cattle) and over ICM. Non-violent conflict is often reported in the invasion literature because it impedes implementation of top-down control efforts (see e.g. Crowley et al. 2017). However, overt conflict is more likely to be revealed when social-ecological relations and well-being are investigated in-depth: of those cases reporting nine or more social-ecological relations and well-being topics, 60% reported conflict. In the Afar (Fig. 3), those pastoralists who continued nomadism moved livestock to new grazing areas in other regions, creating further resource-based conflict (Hamedu 2014). Young herders had to be armed and were increasingly unwilling to assume such risks. Many other conflicts were invasion-related: ‘with charcoal producers, who are generally seen as exploitative outsiders, with commercial plantations which pitch the pastoralists against the formalised bureaucracy and will of the state and with NGOs who promote utilisation strategies perceived as inappropriate’ (Rogers et al. 2017, p. 7). Many pastoralists were forced to resettle, amalgamating people and livestock from different locations and clans in new areas. Socially, ‘dislocation, displacement and distance are undermining traditional Afar social norms and patterns of behaviour…*P. juliflora* acts like…a ‘barbed-wire fence’…which forces a barrier between neighbours and limits the reconciliation of conflict’ (Rogers et al. 2017, p. 7). In Kenya, conflicts between livestock producers who lost grazing land and were forced to resettle were also reported with *Acacia*-dominated bush and *Prosopis* invasions (East Pokot and Baringo-Bogoria case study clusters). In Timor, *Chromolaena* invasion generated conflict between livestock owners and with the government over controls (McWilliam 2000). In all of these cases, cross-scale interactions and external interventions exacerbated pre-existing inequalities, shaped adaptation pathways and undermined local adaptive capacity.

### Adaptation types, pathways and outcomes

Adaptation types, pathways, feedbacks and outcomes in terms of social-ecological system resilience or shifts were discussed above (see Tables 3, 4, 5 and Fig. 3), but analysing these concepts separately serves to clarify the conceptual framework and its application. Table S11 provides definitions and examples.

#### Adaptation types

Table 5 and Fig. 3 present the types of adaptation found in the case studies. All of the types of adaptation identified in Thornton and Manasfi (2010) were also found in the case studies, with the exception of rationing. Innovation was not included, as researchers failed to state whether an adaptation action was novel or represented a new application or an extension of already existing knowledge or techniques. In two cases, for example, invasion was prevented by planting species that may or may not have been novel (e.g. introduced for this purpose), using existing knowledge about managing canopy cover to shade out undesired plants (Dove 1986; Douterlungne et al. 2010).

**Table 5:** Adaptation types and sub-types* in the case studies (*n* = 52 cases or case study clusters)

Type	Sub-type	Case count	% of cases
Mobility	Resource tracking	4	15.4
	Migration	8	5.8
	Resettlement	8	7.7
	Sedentarisation	3	3.8
Diversification	Ecological	8	15.4
	Subsistence	5	9.6
	Wage	8	15.4
	Enterprises	14	26.9
Asset (re)allocation	Pooling	2	3.9
	Individualisation	4	7.7
Species shifts	New invasives uses	21	40.4
	Switched species	18	34.6
	Market sourced	5	9.6
Resource use intensity	Intensification	8	15.4
	Disintensification	9	17.3
	Both	7	13.5
Revitalisation	Governance/cultural	2	3.8
	Conservation	3	5.8
	Restoration	9	1.9
Transformation		2	3.8

*For definitions and examples, see Table S11

Here, the focus is on the types of adaptation that were found to be most strongly related with biodiversity change. The most common type was shifts in species provoked by invasions (as evidenced by the high frequency of new uses of invasives), or that were related to other types of adaptation, such as sedentarisation and intensification (e.g. of agriculture). Adaptations in the ‘intensity of resource use’ are also important for understanding adaptation to biodiversity change. Disintensification (e.g. land abandonment), for example, is often poorly understood and considered as a maladaptation. In many cases, it led to invasive spread, but it also constituted part of complex adaptation pathways that otherwise could not have been pursued. Disintensification can also break positive feedbacks or generate negative feedbacks, increasing social-ecological resilience. Similarly, intensification is also often poorly understood both as a driver and an adaptation to environmental change (see e.g. Morrison 1994; Meyfroidt et al. 2018). In Indonesia, for example, given numerous external and internal pressures as well as *Imperata* invasion, abandoning dryland rice plots and shifting to more intensive wet rice production provided stability for most smallholders (Burkard 2005).

Another important adaptation sub-type related specifically to biodiversity change is ‘resource tracking,’ defined here as ‘human movements associated with change in natural resource availability across space and time.’ Resource tracking is related with foraging, hunting, grazing and even agriculture. It is associated with temporal factors, such as variability in species’ growth, predator and herbivore consumption and movements, and changes in water availability that occur over days, seasons or years, creating resource abundance or scarcity. Especially given climate-driven changes in species ranges and habitat fragmentation, which create spatial discontinuities in species’ abundances, resource tracking allows people to use and mix resources that would otherwise become unavailable (Hobbs et al. 2008). Species invasions often reduce the abundance of useful local species, while resource tracking allows continued access in areas where, for example, the invasive has not had similar impacts.

#### Adaptation pathways

Table 3 and Fig. 3 illustrate the factors influencing the adaptation pathways (or, at minimum, responses) pursued by different social groups; 21 case studies presented sufficient information to allow analysis, where a sample of these is presented in the table. Most researchers didn’t use a pathways concept or clearly differentiate between groups or the pathways these pursued—only one article used this concept (Müller-Mahn and Rettberg 2012). The most common factors influencing adaptation pathways were the use of specific resource areas, assets available to households and the severity of the invasion. In 72% of these cases, adaptation pathways were related with differential use of land, forests or other ecological niches. For example, pastoralists had different adaptation pathways compared with agropastoralists or farmers. Differential access to labour, capital, technology or knowledge influenced adaptation pathways in 86% of the cases, affecting especially the ability to mitigate invasive harms and to diversify. The severity of invasion influenced pathways in 24% of these cases; those experiencing more severe invasions more often pursued diversification and intensification/distensification pathways. Other factors influencing pathways and their outcomes included different uses of invasives (different benefits); specialisation, control methods and gender and other power relations.

#### Social-ecological outcomes: Resilience, instability, regime shifts and transformation

Table 4 presents the majority of cases where sufficient information was available to at least hypothesise real or potential outcomes in terms of stability or regime shifts for those *components* of the social-ecological system under study. In two of the case clusters (in the Afar and in East Pokot, Kenya) study areas and the number of publications were sufficient to draw inferences about entire social-ecological systems.

Resilience was maintained through preventative action in three cases (one of which is presented in the table), all of which were in long-fallow systems (see also Dove 1986; Douterlungne et al. 2010). In Laos, *Chromolaena odorata* was managed to restore resilience in degraded short-fallow systems (Roder et al. 1995a, b). Adaptation increased instability in Nepal’s Chitwan National Park and buffer zone, in part due to the external introduction of new technology that was intended to enhance stability (Rai and Scarborough 2015), but as well due to poor governance (Sullivan et al. 2017). Partial regime shifts that were positive both for ecosystems and human well-being were evident in two cases as people managed invasives for ecological and livelihood diversification (Siges et al. 2005; Tassin and Kull 2015). Negative and at least partial regime shifts were also evident, but these systems may have already undergone full regime shifts and, if not, these are likely in the future in Timor (McWilliam 2000) and Lake Inle, Myanmar (Martin 2014). Full regime shifts are evident in the Middle Awash Basin of Afar, Ethiopia, and in Kaski District, Nepal (Pandey 2017) but, in the former, invasion played a more catalytic role compared with the latter (Table 4).

Anderson and Bollig applied ‘adaptive cycle’ theory (Holling and Gunderson 2002) to understand long-term change in Kenya’s Baringo-Bogoria basin. They argued that the system collapsed in the early 19th century and is currently in the release phase, as pastoralists are fundamentally reorganising their specialised cattle system: ‘the most profound indications of collapse and reorganisation are to be seen in the bio-ecological changes…specifically in relation to the impact of invasive species as a primary driver of the release (Ω) phase’ (Anderson and Bollig 2016, p. 11). Researchers argued that, in the Afar, in the 1990s, the cumulative effects of changed flood regimes and *Prosopis* invasion tipped the region into a state of chronic food insecurity and impoverishment (Rettberg 2010; Müller-Mahn and Rettberg 2012; Mehari 2015; Rogers et al. 2017). Both pastoralist and ecosystem vulnerability increased dramatically. As a social-ecological system, pastoralism provided for high levels of human well-being and biodiversity over a long timeframe. Now,

Whilst the alternatives, mono-cropping, commercial plantations and small-scale cash crops, fail to offer the same level of environmental protection, with no incumbency upon users to preserve the unique ecosystem, they do offer the prospect of better confronting the invasion, a fact which offers the most significant threat to pastoralists. The tragedy of the invaded commons is that all of the co-evolved, ecological sensitivity and specialism is a burden rather than a boon (Rogers et al. 2017, 10).

A very different case, of adaptation leading to transformation, is found in Cameroon (Jagoret et al. 2012). *Imperata* invasion of savannah grasslands presented a major constraint for indigenous Yambassa farmers as it competed with crops for water and nutrients. Farmers used a gradual ‘agro-successional strategy to restore infertile degraded ecosystems,’ converting these grasslands into cacao and palm oil agroforestry systems in a zone that is not considered to be suitable for cacao. While cacao production is considered as a major driver of deforestation, in this case, it resulted in afforestation. Farmers, who efficiently managed trees to ‘maintain shading conditions that they considered optimal for both controlling *I. cylindrica* and cocoa tree growth, showed that it is possible to overcome the presence of I*. cylindrica*, water deficit and irregular rainfall distribution, and poor soil fertility’ (Jagoret et al. 2012, p. 502). Marketable cacao yields were similar to those in other areas. This process, however, led to land privatisation and may eventually lead to shortages of land for annual food or cash crops, which could lead to decreasing soil fertility and increased social tension and, eventually, further feedbacks and adaptation.

## Discussion and conclusions

Invasions drive rapid biodiversity change at local scales. Results of the case study metasynthesis show that not only the invasive, but as well other environmental, economic, socio-political and technological drivers create the conditions for invasibility and spread, influence impacts, and shape human adaptation and feedbacks. In adapting to invasive impacts, people manage invasives and resource systems in ways that increase or mitigate invasions and their impacts, and that create further ‘novel disturbances’ affecting invasibility and invasion dynamics. Adaptation also alters human relations and well-being, which feeds back at different scales into management, ecosystem dynamics and well-being in a complex, non-linear fashion. Different social groups adopt different adaptation types and pathways that are historically shaped and vary depending on contextual factors such as severity of invasion impacts, and possibilities for invasive control and management, including for benefit. Adaptation pathways intersect in space and time and generate diverse feedbacks that strongly affect social-ecological outcomes.

The metasynthesis shows that, in cases where local resource management systems have evolved over fairly long time periods within vacillating ecological environments, and local ecological knowledge has co-evolved to manage biological change, people often prevented invasion, ‘restored’ invaded environments or generated far greater benefits by altering resource systems to create greater biodiversity, use and exchange value and human well-being. In some cases, even in the presence of these conditions, as in the Afar, socio-economic, economic and technological drivers both overwhelmed and undermined local adaptive capacity, generating vicious invasion processes, major positive feedbacks and regime shift. Thus, human capacity to mitigate harms and derive benefits from invasions is, at the end of the day, a dependent variable. Misguided policies, political and economic marginalisation, loss of ecological knowledge and increasing social differentiation and conflict generate poverty, undermine local governance and adaptive capacity, and ensure that the least powerful bear the brunt of such ‘vicious’ invasions.

### Framework application: Policy, practice and research

The HAIS framework begins to fill a major gap in the adaptation to environmental change literature by addressing at least one biodiversity change driver and human adaptation to change provoked by this driver. It also begins to address major lacunae in the invasion science literature; it adopts a social-ecological systems approach to species invasions while integrating human adaptation into this approach. The third gap that remains to be addressed, however, is the renowned ‘knowing-doing’ gap in invasion science (Esler et al. 2010), that is, the gap between science and management.

#### Policy and planned invasive management

Invasion scientists may be very concerned that, given the already insufficient political will to prevent, eradicate or control invasive alien species, ‘adaptation’ in fact will translate into ‘accommodation’ or ‘acceptance’ of invasives, in effect legitimating a failure to act in future.^11^ Under which circumstances, then, should invasive policies and management plans seriously address HAIS as a response option? In the first instance, it must be considered in cases where invasive species are very damaging, and control efforts have failed or not been possible to implement—where there is effectively no other option than to adapt. This includes cases where, due to the type of invasive and invasion scale, resources are not available to effectively mitigate or control invasives, such as in forests, rangelands, savannahs and large water bodies, including oceans. It includes cases where there are no known effective control methods, or effective methods cannot be deployed due to non-target effects or strong political, ethical or social objections to available controls methods. It also applies to cases where an invasive generates substantial social, economic or ecological benefit and has been incorporated into social-ecological systems to such a degree that control or eradication would generate serious negative social-ecological impacts.

Yet the practical and policy implications of HAIS are more profound than this suggests. First and foremost, aside from those cases where invasion scientists and policy makers might consider that adaptation is an ‘acceptable’ option, autochthonous adaptation still occurs everywhere that invasives have an impact on human well-being. As the results above so clearly demonstrate, HAIS has major implications for invasion dynamics and broader social-ecological system feedbacks and outcomes. No matter how wide the reach of planned interventions, invasive management (of targeted *and* non-targeted invasives) will continue to be predominantly autochthonous. Autochthonous adaptation is thus by far the most important means at humanities’ disposal to mitigate invasive impacts, restore social-ecological resilience and, where necessary and possible, transform social-ecological systems to more desirable and sustainable states. Understanding and considering this fact at global, national and local levels can only lead to the formulation of policies and practices (e.g. around land management and pesticide use) that seek to influence autochthonous adaptation in ways that increase adaptive capacity, resilience and sustainability.

In those instances where invasives, other drivers and HAIS generate important positive feedbacks leading to loss of resilience and regime shifts, policies should be formulated to accommodate local contexts. Policies must be based on an understanding of these dynamics before drivers and feedbacks can be changed. To do more good than harm, invasive policy must also consider HAIS at different scales. Case studies often reported that development policies directly or indirectly contributed to invasions—often, governments undervalued, misunderstood and misrepresented local human–environment relations, livelihoods and production systems, often characterising them a priori as unproductive or environmentally destructive. In some cases, policies, regulations and control programmes were introduced that sharply reduced adaptive capacity and increased negative impacts.

Management interventions based on a poor understanding of invasion drivers and dynamics, human adaptation processes and social-ecological outcomes are much more likely to generate positive feedbacks. When different groups pursue different pathways that work at cross-purposes, this is likely to drive forms of adaptation that lead to greater instability and undesired social-ecological outcomes, as in the Afar. In Kenya, however, NGOs and multilateral development organisations supported autochthonous adaptation, introducing technological innovations, market channels and financial support that complemented local knowledge and initiatives (Österle 2008). In another case, researchers were able to suggest ways for NGOs and farmers to enhance local practices to increase biodiversity while maintaining the benefits that farmers derived from the invasive (Awanyo 2007). Positive synergies between policies, planned interventions and autochthonous adaptation can only be achieved when local resource managers are engaged, knowledgeable, willing and prepared to adapt according to pathways that coincide with their values, assets and visions of the future.

#### Caveats for research

The HAIS framework is intended to serve as a guide for future research, with several caveats. The case studies reviewed were often individually quite limited, so that some of the conclusions of the metasynthesis, especially those relating to (hypothetical) social-ecological system outcomes, must be strongly qualified. None of the studies covered all of the categories and interrelations, so many adaptations and feedbacks were missed. Most focused on a single production system rather than the diversity of systems that rural people manage. Adaptation pathways were often under-specified, which requires attention to social diversity and longer timeframes. Many cases differentiated people only within a single social category (e.g. ‘farmers’), overlooking other groups that were also very likely adapting. Few examined changes in ecosystem functions and biological diversity, and none covered entire social-ecological systems. Temporal and spatial scales were generally far too limited to examine adaptation pathways and social-ecological system change. With some exceptions, the examination of feedbacks between adaptation spheres was limited; often, micro- and meso-level change was neglected. This should not be construed as a critique of these studies—rather, it is a reflection of the diversity of case study aims, where none of the studies sought to examine HAIS. However, it also highlights the importance and strength of metanalysis as a tool, which is able to exploit case study diversity to achieve novel goals.

Future HAIS research should therefore consider a social-ecological systems approach, develop social-ecological timelines (Brattland et al. 2019; Thornton et al. 2019) to capture adaptation pathways and change over longer timeframes, address the full range of resources and ecosystems that people manage (including fishing, hunting, gathering, agriculture, livestock, homegardens) as well as the diversity of livelihood strategies pursued, examine social diversity and investigate cross-scale dynamics and the range of variables and feedbacks identified here at micro- and meso-scale. While it is clear that this breadth and depth requires coordinated interdisciplinary research, this proposition is not unrealistic—it is both necessary and worthwhile when the goal is either (a) to advance our understanding of human adaptation to the massive changes in biodiversity that are already underway and that will certainly greatly accelerate in the near term, or (b) to formulate invasive species policies and planned interventions that can genuinely effectuate positive change.

## Electronic supplementary material

Below is the link to the electronic supplementary material.

Supplementary material 1 (PDF 1084 kb)

## Footnotes

1 This is reflected in the GISD Invasive species database (see ‘literature search’ section). Anthromes refer to anthropogenic biomes (Ellis 2011).

2 The Ecosystem Services and Poverty Alleviation Programme (ESPA) of the UK Government funded the first research project on this theme, entitled ‘Human adaptation to biodiversity change: building and testing concepts, methods and tools for understanding and supporting autonomous adaptation’ (in short, ‘HABC’).

3 If HAIS were indeed a field of theorising or methods development, evidence of this would be found in (a) some degree of shared set of scientific references across case studies, including references to conceptual framework or theoretical publications, and/or to methods publications. If it is not, then these common references will be largely lacking (Trujillo and Long 2018). Co-citations bibliometric analysis was performed on references for each of the case studies (four of the case study article references could not be included as they were unavailable electronically). Briefly summarised, the analysis showed that the case studies lack common conceptual or methodological references; authors and research groups were much more likely to cite themselves than to cite other case literature and, with the exception of very few case studies, in general the researchers did not reference other HAIS studies in their work. See Table S1.

4 Howard 2009. ‘Human Adaptation to Biodiversity Change Research Programme: outline and notes toward a tentative conceptual framework.’ University of Kent and Wageningen University, unpublished manuscript.

5 http://www.iucngisd.org/gisd/100_worst.php (accessed 12 February 2018). CABI’s Invasive Species Compendium (https://www.cabi.org/isc) was used to confirm and supplement scientific and common names as search terms.

6 Most publications from disciplines other than invasion biology and ecology (e.g. entomology, plant pathology, weed science) do not use ‘invasive species’ terminology in titles, abstracts or keywords. If invasive terminology is not used, publications are not indexed as such in literature databases. To locate the majority of the literature, databases must be searched for ‘weeds,' ‘insects’ or ‘pathogens’, or for specific control methods (e.g. biological control) or specific invasive species (Howard, P.L. 2019. The conservation-development schism in alien invasive science: a problem for global relevance? Unpublished manuscript).

7 http://www.iucngisd.org/gisd/about.php (accessed 20/5/2019).

8 https://www.cabi.org/isc.

9 Some argue that *Prosopis* was introduced in the late 1970s by international development organisations and the government to rehabilitate degraded soils and supply fuelwood and fodder (Abdulahi et al. 2017); others argue that the introduction pathway is not clear (Mehari 2015; Rogers et al. 2017).

10 Two of these cases were detailed in studies that compared more than one set of adaptations to the invasive (Dove 1986; Bagnall-Oakeley et al. 1996), whereas the third case was included as it contrasted with other studies on the same invasive in nearby regions and provided an example of ‘restoration’ (Douterlungne et al. 2010).

11 Personal communications with several invasive species scientists at the first authors meeting of the IPBES Global Invasive Alien Species Assessment, Tsukuba, Japan, August 2019.
