# Supplementary material for: Human adaptation to invasive species: A conceptual framework based on a case study metasynthesis
Source: Ambio. 2019 Nov 24;48(12):1401–30. doi: 10.1007/s13280-019-01297-5 (PMC6883017; doi:10.1007/s13280-019-01297-5)
Supplement: Supplementary file 1 — Supplementary material 1 (PDF 1084 kb) [file 13280_2019_1297_MOESM1_ESM.pdf]

Ambio

Electronic Supplementary Material
This supplementary material has not been peer reviewed.

Title: Human adaptation to invasive species: A conceptual framework based on a case study metasynthesis

Author: Patricia L. Howard

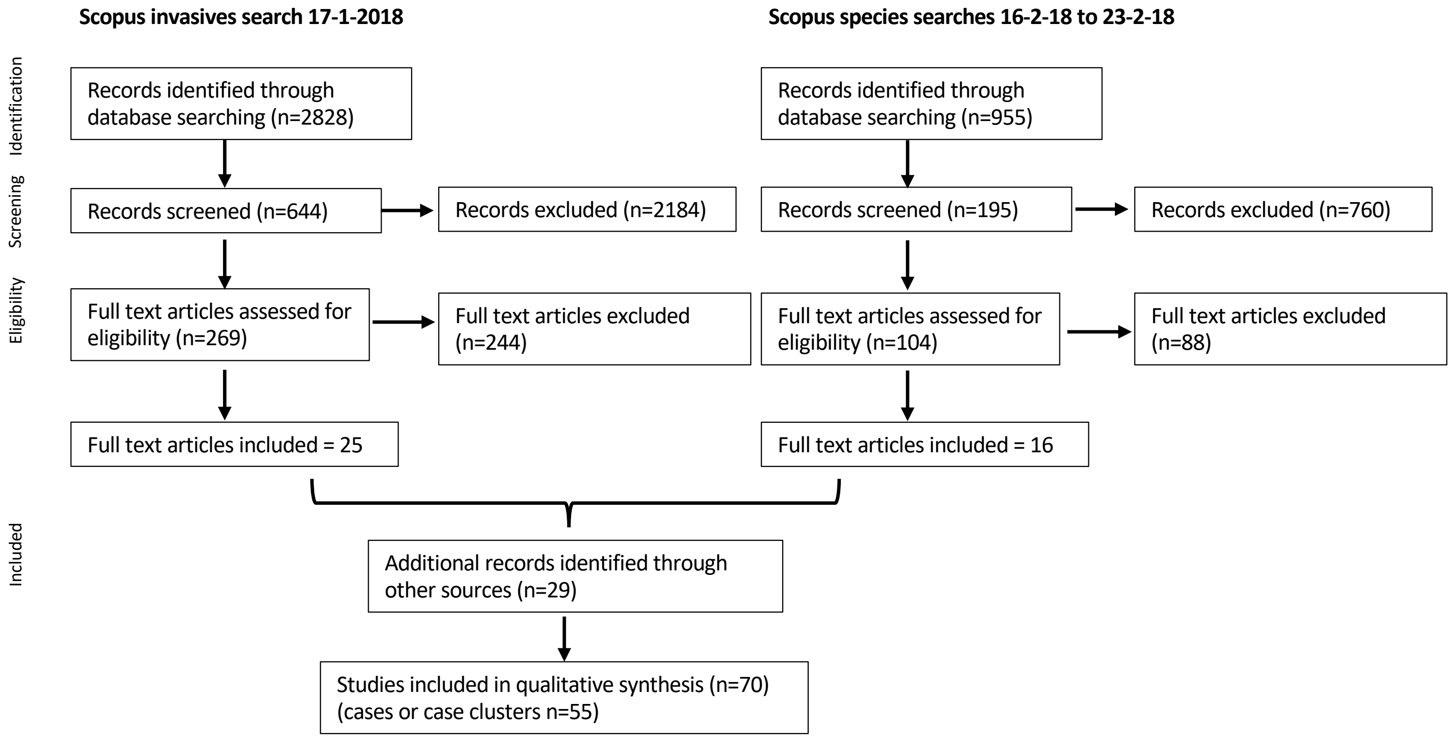

Figure S1. PRISMA* flow diagramme for Scopus literature searches
*Preferred Reporting Items for Systematic Reviews and Meta-Analyses (Moher et al., 2009).

Table S1. Case study authors most frequently cited by number cites and % times cited across all case studies*

Lead authors	Self-citing		Cited by own research group		Cited within case cluster		Cited by other case study authors		Total citations	
	Nr.	%	Nr.	%	Nr.	%	Nr.	%	Nr.	%
Rettberg, S.	15	78.9			3	15.8	1	5.3	19	
Wakie, T.T.	1	16.7			5	83.3			6	
Ilukor, J.	2	66.7			1	33.3			3	
Treydte, A.C.	1	50.0			1	50.0			2	
Ayanu, Y.	2	66.7			1	33.3			3	
Müller-Mahn, D.	1	100.0							1	
Abdulahi, M.M.					2	100.0				
Total	*22*	*61.1*			*13*	*36.1*	*1*	*2.7*	*36*	*17.3*
Shackletons (C.M., R.T., S.)	36	65.5			10	18.2	9	16.4	55	
Van Wilgen, B.W.	3	11.5			20	76.9	3	11.5	26	
Richardson, D.M.	7	20.0			18	51.4	10	28.6	35	
Fabricius, C.					4	100.0			4	
Kull, C.A.	8	100.0							8	
Total	*54*	*42.2*			*52*	*40.6*	*22*	*17.2*	*128*	*61.8*
Bollig, M.	4	22.2	14	77.8					18	
Mwangi, E. & Swallow, B.					2	14.3	12	85.7	14	
Becker, M.	3	60.0	1	20.0			1	20.0	5	
Greiner, C.	1	50.0	1	50.0					2	
Vehrs, H.P.			1	100.0					1	
Österle, M.			3	100.0					3	
Total	8	18.6	20	46.5	2	4.7	13	30.2	43	20.8
Grand total	*84*	*40.6*	*20*	*9.7*	*67*	*32.4*	*36*	*17.4*	*207*	*100.0*

*Total publications referenced = 60. Co-citations bibliometric analysis (see endnote 3) was performed on all references for each of the case studies. All references were downloaded from Scopus (11 December 2018) for each of the cited case studies and corrected for missing information where necessary. Three of the case study article references could not be included as they were unavailable, while references for the Martin 2014 were too numerous and not available electronically.

Table S2. Socio-economic content of GISD '100 Worst Invasive Species' cites in Scopus* and case study invasives as a proportion of these cites

**Total subject area delimited cites***	**No.**	**%**
Total GISD cites	235912	100.00
Total 'invasive' cites	11204	4.75
**Of invasive cites**	**No.**	**% invasive**
'Livelihood' cites*	181	1.62
'Population group'* cites	399	3.56
Combined livelihood/population cites	490	4.37
After screening for socio-economic content	227	2.03
**Top GISD invasives (>200 cites)**	**No.**	**% of total**
Capra hircus, goat	211	1.88
Micropterus salmoides, largemouth bass	213	1.90
Mus musculus, house mouse	217	1.94
Eichhornia crassipes, water hyacinth	226	2.02
Aedes albopictus, Asian tiger mosquito	234	2.09
Salmo trutta, sea trout	258	2.30
Lantana camara, lantana	264	2.36
Carcinus maenas, shore crab, green crab	277	2.47
Linepithema humile, Argentine ant	283	2.53
Solenopsis invicta, red imported fire ant	293	2.62
Rattus rattus, black rat	320	2.86
Oryctolagus cuniculus, rabbit	333	2.97
Sus scrofa, wild boar, feral pig	337	3.01
Oncorhynchus mykiss, rainbow trout	374	3.34
Cyprinus carpio, common carp	379	3.38
Rhinella marina, bullfrog, cane toad	478	4.27
Dreissena polymorpha, zebra mussel	657	5.86
Total	*5354*	*47.79*
**Top invasives with confirmed socio-economic content (5 + cites)**	**No.**	**% socio-economic cites**
Myocastor coypus, coypu	5	2.20
Pomacea canaliculata, golden apple snail	5	2.20
Vulpes vulpes, red fox	5	2.20
Mikania micrantha, bitter vine, mile-a-minute	6	2.64
Oryctolagus cuniculus, rabbit	6	2.64
Dreissena polymorpha, zebra mussel	8	3.52
Melaleuca quinquenervia, paperbark tree	8	3.52
Cyprinus carpio, common carp	9	3.96
Lantana camara, lantana	11	4.85
Lates niloticus, Nile perch, Victoria perch	11	4.85
Oncorhynchus mykiss, rainbow trout	11	4.85
Acacia mearnsii, Australian acacia, black wattle	13	5.73
Chromolaena odorata, triffid weed, Siam weed	14	6.17
Sus scrofa, wild boar, feral pig	15	6.61
Eichhornia crassipes, water hyacinth	37	16.30
Total	164	85.02
**Case study invasives**	**No. cites**	**%**
Of total delimited GISD cites	7336	3.1
Of total invasive cites	1244	11.1
Of total ‘livelihood’ cites	76	42.0
Of total ‘population group’ cites	56	14.0
Of total combined cites	105	21.4
Of total cites after screening	88	38.8

*For search terms and delimiters, see Table 2. Search conducted September 2019. Case study invasives highlighted in pink.

Table S3. Full citations of selected case study publications and case study clusters

Cite & use	Case No.	Invasive	Study site	Full citation
1 - S	1	*Typha domingensis*	Lake Pátzcuaro, Mexico	Hall, S.J. 2009. Cultural disturbances and local ecological knowledge mediate cattail (*Typha domingensis*) invasion in lake Pátzcuaro, México. *Human Ecology* 37: 241–249.
2 - S	2	*Spathodea campanulata*	Viti Levu, Fiji	Brown, P., and A. Daigneault. 2014. Cost-benefit analysis of managing the invasive African tulip tree (*Spathodea campanulata*) in the Pacific. *Environmental Science and Policy* 39: 65–76.
3 - M	3	*Herpestus javanicus*	Viti Levu, Fiji	Daigneault, A., and P. Brown. 2013. Invasive species management in the Pacific using survey data and benefit-cost analysis. Paper presented to the 57th Australian Agricultural & Resource Economics Society Annual Conference, 5-8 February 2013. Sydney, New South Wales, Australia.
	4	*Papuana uninodis*		
	5	*Pycnonotus cafer*		
	6	*Merremia peltata*		
4 - S	7	*Mimosa pigra*	Mekong River, Cambodia	Rijal, S., and R. Cochard. 2016. Invasion of *Mimosa pigra* on the cultivated Mekong River floodplains near Kratie, Cambodia: farmers' coping strategies, perceptions, and outlooks. *Regional Environmental Change* 16: 681–693.
5 - S	8	*Pomacea canaliculata*	Asia	Halwart, M. 1994. The golden apple snail *Pomacea canaliculata* in Asian rice farming systems: present impact and future threat. *International Journal of Pest Management* 40: 199–206.
6 - S	9	*Pomacea canaliculata*	Ifugao Province, Philippines	Joshi, R. C., M.S. Delacruz, E.C. Martin, J.C. Cabigat, R.G. Bahatan, A.D. Bahatan, E.H. Abayao, J. Choy-Awon, et al. 2001. Current status of the golden apple snail in the Ifugao rice terraces, Philippines. *Journal of Sustainable Agriculture* 18: 71–90.
7 - S	10	*Pomacea canaliculata*	Yilan County, Taiwan	Tsai, Y.-L., I. Carbonell, J. Chevrier, and A.L. Tsing. 2016. Golden snail operata: the more-than-human performance of friendly farming on Taiwan's Lanyang Plain. *Cultural Anthropology* 31: 520–544.
8 - S	11	*Pomacea canaliculata*	Ecuador	Horgan, F.G., M.I. Felix, D.E. Portalanza, L. Sánchez, W.M. Moya Rios, S.E. Farah, J.A. Wither, C.I. Andrade, et al. 2014. Responses by farmers to the apple snail invasion of Ecuador's rice fields and attitudes toward predatory snail kites. *Crop Protection* 62: 135–143.
9 - SD		*Supplemental data*		Borbor-Cordova, M.J., E.W. Boyer, W.H. McDowell, and C.A. Hall. 2006. Nitrogen and phosphorus budgets for a tropical watershed impacted by agricultural land use: Guayas, Ecuador. *Biogeochemistry* 79: 135–161.
10 - S	12	*Centaurea solstitialis*	California, USA	Aslan, C.E., M.B. Hufford, R.S. Epanchin-Niell, J.D. Port, J.P. Sexton, and T.M. Waring. 2009. Practical challenges in private stewardship of rangeland ecosystems: yellow starthistle control in Sierra Nevadan foothills. *Rangeland Ecology and Management* 62: 28–37.

Table S3. Full citations of selected case study publications and case study clusters, con't.

Cite & use	Case No.	Invasive	Study site	Full citation
11 - S	13	*Centaurea solstitialis*	California, USA	Eagle, A.J., M.E. Eiswerth, W.S. Johnson, S.E. Schoenig, and G.C. Van Kooten. 2007. Costs and losses imposed on California ranchers by yellow starthistle. *Rangeland Ecology and Management* 60: 369–377.
12 - S	14	*Taeniatherum caput-medusae ssp. asperum*	Oregon, USA	Johnson, D.D., K.W. Davies, P.T. Schreder, and A.M. Chamberlain. 2011. Perceptions of ranchers about medusahead (*Taeniatherum caput-medusae* (L.) Nevski) management on sagebrush steppe rangelands. *Environmental Management* 48: 400–417.
13 - S	15	*Acacia drepanolobium*	Borana rangelands, Ethiopia	Terefe, B., M. Limenih, A. Gure, and A. Angassa. 2011. Impact of *Acacia drepanolobium* (an invasive woody species) on gum-resin resources and local livelihood in Borana, Southern Ethiopia. *Tropical and Subtropical Agroecosystems* 14: 1063–1074.
14 - S	16	*Acacia spp.*	Madagascar, Congo, southern India	Tassin, J., H. Rangan, and C.A. Kull. 2012. Hybrid improved tree fallows: harnessing invasive woody legumes for agroforestry. *Agroforestry Systems* 84: 417–428.
15 - S	17	*Opuntia stricta*	Central Kenya	Shackleton, R.T., A.B. Witt, F.M. Piroris, and B.W. van Wilgen. 2017a. Distribution and socio-ecological impacts of the invasive alien cactus *Opuntia stricta* in eastern Africa. *Biological Invasions* 19: 2427–2441.
16 - S	18	*Piper aduncum*	Finschhafen District, Papua New Guinea	Siges, T.H., A.E. Hartemink, P. Hebinck, and B.J. Allen. 2005. The Invasive shrub *Piper aduncum* and rural livelihoods in the Finschhafen area of Papua New Guinea. *Human Ecology* 33: 875–893.
17 - SD		*Supplemental data*		Hartemink, A.E. 2010. The invasive shrub *Piper aduncum* in Papua New Guinea: a review. *Journal of Tropical Forest Science* 22: 202–213.
18 - S	19	*Various invasives*	Kaski District, Nepal	Pandey, R. 2017. Farmers' perception on agro-ecological implications of climate change in the Middle-Mountains of Nepal: a case of Lumle Village, Kaski. *Environment, Development and Sustainability*: 1–27.
19 - A	20	*Mikania micrantha*	Chitwan National Park & Buffer Zone, Nepal	Murphy, S.T., N. Subedi, S.R. Jnawali, B R. Lamichhane, G P. Upadhyay, R. Kock, and R. Amin. 2013. Invasive *Mikania* in Chitwan National Park, Nepal: the threat to the greater one-horned rhinoceros *Rhinoceros unicornis* and factors driving the invasion. *ORYX* 47: 361–368.
20 - A				Rai, R.K., and H. Scarborough. 2015. Economic value of mitigation of plant invaders in a subsistence economy: incorporating labour as a mode of payment. *Environment and Development Economics* 18: 225–244.

Table S3. Full citations of selected case study publications and case study clusters, con't.

Cite & use	Case No.	Invasive	Study site	Full citation
21 - A	20 con't.	*Mikania micrantha*	Chitwan National Park & Buffer Zone, Nepal	Rai, R.K., and H. Scarborough. 2015. Understanding the effects of invasive plants on rural forest-dependent communities. *Small-scale Forestry* 14: 59–72.
22 - A				Sullivan, A., A.M. York, D.D. White, S.J. Hall, and S.T. Yabiku. 2017a. *De jure* versus *de facto* institutions: trust, information, and collective efforts to manage the invasive mile-a-minute weed (*Mikania micrantha*). *International Journal of the Commons* 11: 171–199.
23 - A				Sullivan, A., A.M. York, L. An, S.T. Yabiku, and S.J. Hall. 2017b. How does perception at multiple levels influence collective action in the commons? The case of *Mikania micrantha* in Chitwan, Nepal. *Forest Policy and Economics* 80: 1–10.
24 - A				Khadka, A. 2017. Assessment of the perceived effects and management challenges of *Mikania micrantha* invasion in Chitwan National Park buffer zone community forest, Nepal. *Heliyon* 3.
25 - S	21	*Euryops Floribundus*	Eastern Cape Province, South Africa	Shackleton, C.M., and J. Gambiza. 2008. Social and ecological trade offs in combating land degradation: the case of invasion by a woody shrub (*Euryops floribundus*) at Macubeni, South Africa. *Land Degradation and Development* 19: 454–464.
26 - S	22	*Imperata cylindrica*	Central Sulawesi, Indonesia	Burkard, G. 2005. Sawah first! The cultural ecology of *alang-alang* in a rain forest margin community. *Journal of Agriculture and Rural Development in the Tropics and Subtropics* 106: 1–14.
27 - S	23	*Imperata cylindrica*	Northern Lao PDR	Keoboualapha, B., S. Simaraks, A. Jintrawet, T. Onpraphai, and A. Polthanee. 2013. Farmers' perceptions of *Imperata cylindrica* infestation in a slash-and-burn cultivation area of Northern Lao PDR. *Southeast Asian Studies* 2: 583–598.
28 - M	24	*Imperata cylindrica*	SE Asia, "standard rubber"	Bagnall-Oakeley, H., C. Conroy, A. Faiz, A. Gunawan, A. Gouyon, E. Penot, S. Liangsutthissagon, H.D. Nguyen, et al. 1996. *Imperata* management strategies used in smallholder rubber-based farming systems. *Agroforestry Systems* 36: 83–104.
	25		Sumatra & Kalimantan, Indonesia, "jungle rubber"	
29 - S	26	*Imperata cylindrica*	West Africa	Chikoye, D., V.M. Manyong, and F. Ekeleme. 2000. Characteristics of speargrass (*Imperata cylindrica*) dominated fields in West Africa: crops, soil properties, farmer perceptions and management strategies. *Crop Protection* 19: 481–487.
30 - S	27	*Imperata cylindrica*	Nigeria	Chikoye, D., J. Ellis-Jones, G. Tarawali, P. Kormawa, O. Nielsen, S. Ibana, and T.R. Avav. 2006. Farmers' perceptions of the speargrass (*Imperata cylindrica*) problem and its control in the lowland sub-humid savannah of Nigeria. *Journal of Food, Agriculture and Environment* 4: 118–126.

Table S3. Full citations of selected case study publications and case study clusters, con't.

Cite & use	Case No.	Invasive	Study site	Full citation
31 - S	28	*Imperata cylindrica*	Benin, Cameroon, Ghana, Nigeria	Chikoye, D., F. Ekeleme, and J.T. Ambe. 1999. Survey of distribution and farmers' perceptions of speargrass [*Imperata cylindrica* (L.) Raeuschel] in cassava-based systems in West Africa. *International Journal of Pest Management* 45: 305–311.
32 - S	29	*Imperata cylindrica*	Central Cameroon	Jagoret, P., I. Michel-Dounias, D. Snoeck, H.T. Ngnogué, and E. Malézieux. 2012. Afforestation of savannah with cocoa agroforestry systems: a small-farmer innovation in central Cameroon. *Agroforestry Systems* 86: 493–504.
33 - S	30	*Eichhornia crassipes*	Lake Inle, Myanmar	Martin, M. 2014. *The gardener and the fisherman in globalization: the Inle Lake (Myanmar) in globalization: a region under transition.* Masters Dissertation in Geography, Lyon, France: University Lyon 2 Lumière.
34 - S	31	*Chromolaena odorata*	Mara region, Tanzania	Shackleton, R.T., A.B. Witt, W. Nunda, and D.M. Richardson. 2017b. *Chromolaena odorata* (Siam weed) in eastern Africa: distribution and socio-ecological impacts. *Biological Invasions* 19: 1285–1298.
35 - S	32	*Chromolaena odorata*	Eastern region, Ghana	Awanyo, L. 2007. A Janus-faced biodiversity change and the partiality of ecological knowledge in a world biodiversity hotspot in Ghana: implications for biodiversity rehabilitation. *Geoforum* 38: 739–751.
36 - M	33	*Chromolaena odorata*	Timor	McWilliam, A. 2000. A plague on your house? Some impacts of *Chromolaena odorata* on Timorese livelihoods. *Human Ecology* 28: 451–469.
	34	*Lantana camara*		
37 - A	35	*Chromolaena odorata*	Northern Lao PDR	Roder, W., S. Phengchanh, B. Keoboualapha, and S. Maniphone. 1995a. *Chromolaena odorata* in slash-and-burn rice systems of Northern Laos. *Agroforestry Systems* 31: 79–92.
38 - A				Roder, W., S. Phengchanh, and B. Keoboulapha. 1995b. Relationships between soil, fallow period, weeds and rice yield in slash-and-burn systems of Laos. *Plant and Soil* 176: 27–36.
39 - A				Roder, W. 2001. *Slash-and-burn rice systems in the hills of northern Lao PDR: description, challenges, and opportunities*. Manila, Philippines: International Rice Research Institute (IRRI).
40 - M	36	*C. odorata & I. cylindrica*	Indonesia (drivers only, excluded from all other analyses)	Dove, M.R. 1986. The practical reason of weeds in Indonesia: peasant vs. state views of *Imperata* and *Chromolaena*. *Human Ecology* 14: 163–190.

Table S3. Full citations of selected case study publications and case study clusters, con't.

Cite & use	Case No.	Invasive	Study site	Full citation
40 – M con't.	37	*Imperata cylindrica*	Central Java, Javanese, short *Imperata* fallow	Dove, M.R. 1986. The practical reason of weeds in Indonesia: peasant vs. state views of *Imperata* and *Chromolaena*. *Human Ecology* 14: 163–190.
	38	*Imperata cylindrica*	South Sumatra, Ogan, long forest fallow	
	39	*Imperata cylindrica*	Southeast Kalimantan, Banjarese, short *Imperata* fallow	
	40	*Chromolaena odorata*	Southeast Kalimantan, Banjarese, short bush fallow	
	41	*Chromolaena odorata*	Mount Tambura, Bimanese farmers, long fallow	
	42	*Chromolaena odorata*	Mount Tambura, Bimanese farmers, short bush fallow	
	43	*I. cylindrica & C. odorata*	Mount Tambura, Proto-Banjarese Dayak farmers, long forest fallow	
41 - S	44	*Pteridium aquilinum*	Oaxaca, Mexico	Berget, C., E. Duran, and D.B. Bray. 2015. Participatory restoration of degraded agricultural areas invaded by bracken fern (*Pteridium aquilinum*) and conservation in the Chinantla Region, Oaxaca, Mexico. *Human Ecology* 43: 547–558.
42 - M	45	*Pteridium aquilinum*	Southern Yucatan, large ejido	Schneider, L., and J. Geoghegan. 2006. Land abandonment in an agricultural frontier after a plant invasion: the case of bracken fern in southern Yucatán, Mexico. *Agricultural and Resource Economics Review* 35: 167–177; Schneider, L.C. 2006. Invasive species and land-use: the effect of land management practices on bracken fern invasion in the region of Calakmul, Mexico. *Journal of Latin American Geography* 5: 91–107.
43 - M	46		Southern Yucatan, small ejido	
44 - S	47	*Pteridium aquilinum*	Chiapas, Mexico	Douterlungne, D., S.I. Levy-Tacher, D.J. Golicher, and F.R. Dañobeytia. 2010. Applying indigenous knowledge to the restoration of degraded tropical rain forest clearings dominated by bracken fern. *Restoration Ecology* 18: 322–329.

Table S3. Full citations of selected case study publications and case study clusters, con't.

Cite & use	Case No.	Invasive	Study site	Full citation
45 - S	48	*Prosopis juliflora*	Invasive range, South Africa	Shackleton, R.T., D.C. Le Maitre, and D.M. Richardson. 2015. Stakeholder perceptions and practices regarding *Prosopis* (mesquite) invasions and management in South Africa. *Ambio* 44: 569–581.
46 - A	49	*Prosopis juliflora, Dodonaea viscosa*	Baringo-Bogoria Basin, Kenya	Mwangi, E., and B. Swallow. 2008. *Prosopis juliflora* invasion and rural livelihoods in the Lake Baringo Area of Kenya. *Conservation and Society* 6(2): 130.
47 - A				Becker, M., M. Alvarez, G. Heller, P. Leparmarai, D. Maina, I. Malombe, M. Bollig, and H. Vehrs. 2016. Land-use changes and the invasion dynamics of shrubs in Baringo. *Journal of Eastern African Studies* 10: 111–129.
48 - A	50	*Acacia spp. bush encroachment*	East Pokot, Baringo District, Kenya	Vehrs, H.P. 2016. Changes in landscape vegetation, forage plant composition and herding structure in the pastoralist livelihoods of East Pokot, Kenya. *Journal of Eastern African Studies* 10: 88–110.
49 - A				Greiner, C., and I. Mwaka. 2016. Agricultural change at the margins: adaptation and intensification in a Kenyan dryland. *Journal of Eastern African Studies* 10: 130–149.
50 - A				Österle, M. 2008. From cattle to goats: the transformation of East Pokot pastoralism in Kenya. *Nomadic Peoples* 12: 81–91.
51 - SD	49, 50	*Supplemental data*	Baringo-Borgoria Basin, East Pokot, Kenya	Bollig, M., and A. Schulte. 1999. Environmental change and pastoral perceptions: degradation and indigenous knowledge in two African pastoral communities. *Human Ecology* 27: 493–514.
52 - SD				Anderson, D.M., and M. Bollig. 2016. Resilience and collapse: histories, ecologies, conflicts and identities in the Baringo-Bogoria basin, Kenya. *Journal of Eastern African Studies* 10: 1–20.
53 - A	51	*Prosopis juliflora*	Middle Awash Basin, southern Afar State, including Baadu	Rettberg, S. 2010. Contested narratives of pastoral vulnerability and risk in Ethiopia's Afar region. *Pastoralism* 1: 248–273.
54 - A				Müller-Mahn, D., S. Rettberg, and G. Getachew. 2010. Pathways and dead ends of pastoral development among the Afar and Karrayu in Ethiopia. *The European Journal of Development Research* 22: 660–677.
55 - A				Müller-Mahn, D., and Rettberg, S. 2012. Human-Environment interactions: the invasion of *Prosopis Juliflora* in the drylands of Northeast Ethiopia. In *Changing deserts – integrating people and their environments.*, ed. Mol, L.; Sternberg, T., 297–316. Cambridge, UK: Whitehorse Press.

Table S3. Full citations of selected case study publications and case study clusters, con't.

Cite & use	Case No.	Invasive	Study site	Full citation
56 - A	51, con't.	*Prosopis juliflora*	Middle Awash Basin, southern Afar State, including Baadu	Datona, M. 2014. Socio-economic impacts of *Prosopis juliflora*-related charcoal trade in Gewane Woreda, Afar region. In *Managing* Prosopis Juliflora *for better (agro-) pastoral livelihoods in the Horn of Africa. Proceedings of the regional conference May 1 - May 2, 2014, Addis Ababa, Ethiopia*, 129–136. Bonn and Eschborn, Germany: Deutsche Gesellschaft für Internationale Zusammenarbeit (GIZ) GmbH.
57 - A				Ayanu, Y., A. Jentsch, D. Müller-Mahn, S. Rettberg, C. Romankiewicz, and T. Koellner. 2014. Ecosystem engineer unleashed: *Prosopis juliflora* threatening ecosystem services? *Regional Environmental Change* 15: 155–167.
58 - A				Hamedu, H. 2014. Socioeconomic and ecological impacts of *Prosopis juliflora* invasion in Gewane and Buremudaytu woredas of the Afar Region. In *Managing* Prosopis Juliflora *for better (agro-) pastoral livelihoods in the Horn of Africa (op. cit).*
59 - A				Rettberg, S. 2014. The spread of *Prosopis juliflora* in the wetlands of the Middle Awash Basin. In *Managing* Prosopis Juliflora *for better (agro-) pastoral Livelihoods in the Horn of Africa (op. cit).*
60 - SD		*Supplemental data*		Abdulahi, M.M., J.A. Ute, and T. Regasa. 2017. *Prosopis Juliflora* l: distribution, impacts and available control methods in Ethiopia. *Tropical and Subtropical Agroecosystems* 20: 75–89.
61 - A	52		Gewane, Buremudaytu, and Amibara woredas, Middle Awash Basin	Mehari, Z.H. 2015. The invasion of *Prosopis juliflora* and Afar pastoral livelihoods in the Middle Awash area of Ethiopia. *Ecological Processes* 4: 1–9.
62 -A				Wakie, T.T., M. Laituri, and P.H. Evangelista. 2016. Assessing the distribution and impacts of *Prosopis juliflora* through participatory approaches. *Applied Geography* 66: 132–143.
63 - A				Ilukor, J., S. Rettberg, A. Treydte, and R. Birner. 2016. To eradicate or not to eradicate? Recommendations on *Prosopis juliflora* management in Afar, Ethiopia, from an interdis-ciplinary perspective. *Pastoralism* 6.
64 - A				Rogers, P., F. Nunan, and A.A. Fentie. 2017. Reimagining invasions: the social and cultural impacts of *Prosopis* on pastoralists in southern Afar, Ethiopia. *Pastoralism* 7.
65	53	*Prosopis juliflora*	Allideghi Wildlife Re-serve, Oromia Region, Ethiopia	Kebede, A.T., and D.L. Coppock. 2015. Livestock-mediated dispersal of *Prosopis juliflora* imperils grasslands and the endangered Grevy's zebra in northeastern Ethiopia. *Rangeland Ecology and Management* 68: 402–407.

Table S3. Full citations of selected case study publications and case study clusters, con't.

Cite & use	Case No.	Invasive	Study site	Full citation
66 - A	54	*Lantana camara*	Western Ghats, India - basket makers	Kannan, R., C.M. Shackleton, and R.U. Shaanker. 2014. Invasive alien species as drivers in socio-ecological systems: local adaptations towards use of *Lantana* in Southern India. *Environment, Development and Sustainability* 16: 649–669.
67 - A				Kannan, R., C.M. Shackleton, S. Krishnan, and R.U. Shaanker. 2016. Can local use assist in controlling invasive alien species in tropical forests? The case of *Lantana camara* in southern India. *Forest Ecology and Management* 376: 166–173.
68 - A	55	*Lantana camara*	Western Ghats, Soliga & Lingayat tribes	Sundaram, B., S. Krishnan, A. J. Hiremath, and G. Joseph. 2012. Ecology and impacts of the invasive species, *Lantana camara*, in a social-ecological system in South India: perspectives from local knowledge. *Human Ecology* 40: 931–942.
69 - A				Kent, R., and A. Dorward. 2014. Livelihood responses to *Lantana camara* invasion and biodiversity change in southern India: application of an asset function framework. *Regional Environmental Change* 15: 353–364.
70 - S	56	*Lantana camara*	Central Uganda	Shackleton, R T., A.B. Witt, W. Aool, and C.F. Pratt. 2017c. Distribution of the invasive alien weed, *Lantana camara*, and its ecological and livelihood impacts in eastern Africa. *African Journal of Range and Forage Science* 34: 1–11.

Table S4. HAIS Framework Drivers. Categories, definitions and case study examples

FIRST-TIER DRIVER CATEGORY: ENVIRONMENTAL

External conditions, resources, stimuli etc. with which an organism interacts, including all of the biotic and abiotic factors that surround and affect the survival and development of an organism or population, not otherwise classifiable

First-tier type	Second-tier sub-types	Second-tier sub-type definitions	Examples: direct	Examples: indirect	Case study
**Climate related**			Increase in abundance and altitudinal extension of invasive species.	Increase in pests & diseases, other drivers, led farmers to abandon agriculture, increasing invasion.	Various, Kaski District, Nepal (Pandey, 2017)
**Water related**	Water levels	Weather conditions, species composition, abiotic causes	Rising lake level provides water for lateral spread of invasive.	Main river sometimes runs dry due to course changes & flood water irregularity, reducing grazing land and leading to overgrazing, leading to spread.	*P. juliflora,* Kenya and Ethiopia (case study clusters)
	Water nutrients	Unspecified	Increases invasion of plants that are nutrient-demanding.		*E. crassipe*s, Myanmar (Martin, 2014)
	Drought / water shortage	Climate, biotic & abiotic change	Invasive is drought-resistant; livestock consume pods during drought, dispersing invasive further.	Drought events led to livestock mortality & drove land-use change, tillage, burning & increased pasture stocking densities, which drove invasion.	*P. juliflora,* Kenya and Ethiopia (case study clusters)
	Flooding		Floods disperse invasive.	Floods stopped control efforts, so area was re-invaded.	*P. juliflora,* Kenya & Ethiopia (case study clusters)
**Vegetation related**	Bush encroachment	Change in bush:grass ratio from rainfall, fire, grazing seasonality	Itself may be considered as an invasion.	Bush encroachment led to overgrazing which increases other invasions.	*D. viscosa*, Kenya (E. Pokot case study cluster)
	Fire regime	Naturally occurring fires from e.g. storms, litter accumulation, etc., plus accidental fires, affected by and affecting vegetation	Invasive is fire intolerant.	Soil erosion may be due to crop land abandonment or recovery without rehabilitation; grazing pressure follows. High grazing pressure leads to lower fire frequency & intensity, leading to invasion.	E. *floribundus*, South Africa (Shackleton and Gambiza, 2008)
**Soil-related**		Wind & water erosion, unspecified change in soils; unspecified	Invasive distribution largely determined by soil moisture.		*I. cylindrica*, W. Africa (Chikoye 1999)
**Livestock-related**	Overgrazing / overstocking	Unspecified	Invades degraded areas subject to heavy grazing.	See above	*E. floribundus*, South Africa (Shackleton & Gambiza, 2008)

FIRST-TIER DRIVER CATEGORY: ENVIRONMENTAL, con't.					
**First-tier type**	**Second-tier sub-types**	**Second-tier sub-type definitions**	**Examples: direct**	**Examples: indirect**	**Case study**
**Land-related**	Land abandon-ment	Unspecified	Crop land abandonment provides invasion nuclei.	Grazing land abandonment led to ces-sation of management (e.g. prescribed burning which controls invasive), allowing further invasion.	*E. floribundus,* South Africa (Shackleton and Gambiza 2008)
	Disturbance	Unspecified	Invasive aggressively colonises fallows.	Long history of disturbance in forest reserve from agriculture, road building, quarrying, and bamboo extraction changed forest structure, composition, diversity, richness and, with other drivers, to invasion.	*P. aduncum*, Papua New Guinea (Siges et al. 2005)
**Forest – tree-related**	Forest biodiversity / use decline		Poor regeneration and declining natural bamboo stock bamboo (mast flow-ering).	Long history of disturbance in forest reserve from agriculture, road building, quarrying, and bamboo extraction led to change in forest structure, composi-tion, diversity, richness and, with other drivers, invasion.	*L. camara*, India (case study cluster)
	Deforestation	Climate change, fire, storms, floods, pests & diseases; unspecified	Farmers identified drivers of increasing invasion and damage severity: deforestation (among others), no indirect driver identified.		*I. cylindrica,* Nigeria (Chikoye et al. 2006)
**Invasive use**		Loss /reduction in suitable native species	Useful native plant species decreased with invasion.	Initially controlled, but outside promo-tion of economic use increased demand, reducing demand for native species and reducing local controls, increasing invasive spread.	*T. domingensis*, Mexico (Hall 2009)
**Livelihood-related**	High dependence on wild plant use	Wild plant species' abundance, richness, diversity	Decline in forest succession from disturbance (see 'disturbance').	High dependence on forest successions for wild plant resources; high depend-ence on NTFPs led to invasive use.	*P. aduncum*, Papua New Guinea (Siges et al. 2005)
	Land pressure	Land resource degrada-tion, general (soil, vegetation, water) general	Degraded rangelands.	Degraded rangelands permit invasions, increasing degradation; cattle displaced to highlands where burning is uncon-trolled; grazing contributes to wide-spread soil erosion & land degradation.	*L. camara* & *C. odorata*, Timor (McWilliams 2000)

**FIRST-TIER DRIVER CATEGORY: ENVIRONMENTAL,** con't.					
**First-tier type**	**Second-tier sub-types**	**Second-tier sub-type definitions**	**Examples: direct**	**Examples: indirect**	**Case study**
**Livelihood-related, con't.**	Market pressure	Unsustainable commercial resource use, general	Forest disturbance, change in structure, composition, and invasion.	Indiscriminate large-scale commercial bamboo harvesting.	*L. camara*, India (Kannan et al. 2014)
	Human mobility	Spatial & temporal shifting of population for resource use and consumption	Population movements to search for resources (e.g. NTFPs, forage) spreads invasive.	Movements out of community forests into national park in search of NTFPs leads to invasive spread.	*M. micrantha*, Nepal (Murphy et al. 2013)

**FIRST-TIER DRIVER CATEGORY: ECONOMIC**<br>System of production and exchange in which decisions around investment, production, and distribution are guided by price signals				
**First-tier type**	**Second-tier sub-types**	**Second-tier sub-type definitions**	**Examples: indirect**	**Case study**
**Water-related**	Water nutrients	Discharge from industries, intensive agriculture/ livestock, tourism, etc.	Intensive floating garden agriculture, agricultural runoff from catchment areas, and runoff from mines and tourist facilities led to higher nutrient levels and eutrophication, stimulating water hyacinth spread.	*E. crassipes*, Myanmar (Martin, 2014)
**Soil-related**	Erosion, nutrients, salinity, moisture, biology	Intensive cultivation / grazing for commercial use.	Large-scale commercial irrigated agriculture led to soil salinization, tolerated by *P. juliflora*, contributing to invasion.	*P. juliflora*, Ethiopia (Rettberg & Müller-Mahn 2012)
**Livestock-related**	Livestock / grazing increase	Market driven - market integration, prices, competition, profit-seeking.	Private ranchers clear much land but don't always invest labour to maintain pastures. Opened areas are invaded.	*P. aquilinum*, Mexico (Schneider & Geoghegan 2006)
	Livestock / grazing decrease	Decrease due to declining commercial profitability of livestock.	Increasing land prices spur housing development and intensive agriculture, putting pressure on ranching; ranching is declining. Most ranchers focus on short-term solutions rather than long-term control.	*C. solstitialis*, USA (Aslan et al. 2009)
**Land-related**	Land use change	From competing commercial land uses that e.g. raise land price above what land users are able to pay.		
**Agriculture-related**	Intensification	To increase productivity and market access from market integration, prices, competition, profit-seeking.	High input, intensive rice production for market; main concerns are economic efficiency and agro-technical performance. Invasive controlled with pesticides that harm a natural invasive predator and increase crop pests & diseases.	*P. canaliculata*, Ecuador (Horgan et al., 2014; Borbor-Cordova et al. 2006]

FIRST-TIER DRIVER CATEGORY: ECONOMIC, con't.				
**First-tier type**	**Second-tier sub-types**	**Second-tier sub-type definitions**	**Examples: indirect**	**Case study**
**Agriculture-related,** con't.	Shortening of fallow periods	To increase production per unit of land due to market integration, prices, competition, profit-seeking.	Timorese swidden systems evolved with the Colonial plantation system & is a product of Colonial intervention, which oriented swiddens for market production. Created a less biologically diverse, more extensive form of agriculture that encroached into and burned forests with a reduction in fallows and inadequate fallow management.	*L. camara* & *C. odorata*, Timor (Shepherd & Palmer 2015; McWilliams 2000).
	Fallow improvement	Market driven - market integration, prices, competition, profit-seeking.	Desire for fallow products leads farmers to create 'hybrid' 'improved' fallows based on *Acacia* spp. - 'hybrid' as they are partly natural and partly managed; 'improved' as they are managed to yield greater benefits compared with natural fallows. Market-oriented and profitable.	*Acacia* spp., Madagascar, Congo, India (Tassin et al. 2012)
	Displacement of traditional agriculture	Market competition, land value increase	Invasion due to abandonment of dryland rice plots; area of invaded dryland rises with the area of wet rice; insufficient labour available to cultivate and weed both. Subsistence and market production, formerly done in one swidden system, separated into two - irrigated rice and non-irrigated rice.	*I. cylindrica*, Indonesia (Burkard 2005)
**Forest-related**	Forest management	Market driven - market integration, prices, competition, profit-seeking	Large-scale unsustainable commercial bamboo extraction led to change in forest structure, composition, diversity, richness and, with other drivers, to invasion.	*L. camara*, India (Kannan et al. 2014)
	Forest use intensification; agroforestry	Market driven - integration, prices, competition, profit-seeking	Local farmers' conversion of *Imperata* invaded grasslands to cocoa agroforestry is economically viable.	*I.cylindrica,* Cameroon (Jagoret et al. 2012)
	Forest biodiversity / use decline	Declining profitability of forest use	Drastic reduction in bamboo due to commercial over-exploitation in part led to invasion; invasive used as a substitute and is now commercially exploited for livelihoods.	*L. camara*, India (Kannan et al. 2014)
	Deforestation	For commercial agriculture, plantations, ranching, mining, logging, etc.	Government road construction promoting colonisation led to immigration, large-scale permanent forest clearance, land use intensification for ranching and agriculture, soil compaction, changes in ecological processes, and loss of above- and belowground species diversity –directly causing invasion.	*P. aquillnum*, Mexico (Douterlungne et al. 2010)

FIRST-TIER DRIVER CATEGORY: ECONOMIC, con't.				
**First-tier type**	**Second-tier sub-types**	**Second-tier sub-type definitions**	**Examples: indirect**	**Case study**
**Invasive-related**	Invasive use	Market driven invasive use; profitability of invasive use.	Charcoal made from invasive; producers harvest native trees for high quality charcoal, increasing spread. Associations licensed to produce, but subsequently banned due to environmental & social damage & limited benefits; possible that restrictions are ignored as little effective government oversight.	*P. juliflora*, Ethiopia (Wakie et al. 2016)
**Livelihood related**	High dependence on wild plant use	Profitability of wild plant use for commercial purposes.	Rubber and other valuable trees produced for commercial purposes in long-fallow systems where vegetation is managed to prevent invasion.	*I. cylindrica*, Indonesia - 'jungle rubber system' (Bagnall-Oakeley et al 1996)
	High dependence on wild plant use	Profitability of wild plant use for commercial purposes.	Rubber and other valuable trees produced for commercial purposes in long-fallow systems where vegetation is managed to prevent invasion.	*I. cylindrica*, Indonesia - 'jungle rubber system' (Bagnall-Oakeley et al 1996)
	Land pressure	Cost of land, other factors of production for intensification.	The larger the total land area available (ejido), the greater the invasion. Lowest density where agricultural land is scarce and farmed more intensively; highest in areas with land surpluses and less intensive cultivation. Farmers with high land pressure seek to pay for access to better parcels but opportunities are limited.	*P. aquillnum*, Mexico (Schneider & Geoghegan 2006)
	Market pressure	Prices, costs, competition drive resource management decisions.	Ranchers with invasions more likely to report marginal effectiveness and return on investment with controls	*T. caput-medusae* spp. *asperum*, USA (Johnson et al 2011).
	Human population pressure	Vis a vis existing income opportunities.	Farmland abandonment associated with climate change & increased emigration - land abandonment is severe; agricultural abandonment encourages spread of invasives. HHs are marginal with very limited resources.	*M. micrantha* and other invasives, Nepal (Pandey 2017)
	Labour availability	Access to / cost of wage labour.	Short *C. odorata* fallows predominate where access to wage labour is scarce; farmers seek to maximise returns to labour - there is less labour demand in *C. odorata* fallows compared with *I. cylindrica* fallows.	*C. odorata* and *I. cylindrica*, Indonesia (Dove 1986, various cases)
	Capital availability	Access to income, savings, credit, etc.	Many pastoralists who would like to sedentarise and engage in agriculture in response to invasion and many other stressors don't have access to the required capital.	*P. juliflora*, Ethiopia (Hamedu 2014)

FIRST-TIER DRIVER CATEGORY: ECONOMIC, con't.				
**First-tier type**	**Second-tier sub-types**	**Second-tier sub-type definitions**	**Examples: indirect**	**Case study**
**Livelihood-related,** con't.	Human mobility	Access to means of transport, cost.	Harvesting of invasive has declined due to lack of access to canoes, among others, contributes to lack of control.	*T. domingensis*, Mexico (Hall 2009)
	Off-farm work, emigration	Significant as a proportion of HH income.	Cost of control is high in terms of labour. With low land pressures & other income sources, labour investment not made.	*P. aquillnum*, Mexico (Schneider & Geoghegan 2006)
	Social status	Economic marginalisation – exclusion from participating in the larger economic system in beneficial ways.	Pastoralists consider the loss of grazing land to *P. juliflora* as an 'autocratic governmental intervention that disregarded their interests' (p. 163); government does not have pastoralists' interests in mind.	*P. juliflora*, Ethiopia (case study cluster)
**FIRST-TIER DRIVER CATEGORY: SOCIO-POLITICAL** Non-market forces influencing decision-making, including participation in governance, distribution of social and political power (rights, duties, obligations, enforcement capacity), and dispute resolution mechanisms; includes subsistence-oriented production decision-making, and non-market influences on subsistence.				
**Water-related**	Water levels	Management neglect - persistent failure to meet a set of basic requirements to maintain (e.g. a hydraulic) system in a reasonable functional state.	State farms were abandoned with regime collapse so irrigation infrastructure, dikes & dams not maintained, leading to river diversion, changes in water levels, and further *Prosopis* spread.	*P. juliflora*, Ethiopia (Rettberg & Müller-Mahn 2012)
	Drought /water shortage		Irrigation infrastructure not maintained, leading to flooding; large areas become inaccessible to pastoralists from flooding, leading to greater livestock mobility and spread of invasive; main river also runs dry due to course changes & flood water irregularity, which favours invasive.	*P. juliflora*, Ethiopia (case study cluster)
	Flooding			
**Vegetation-related**	Fire regime	Restrictions on use of fire prohibited in a prescribed area under specific conditions to achieve a desired outcome.	A government ban on the use of traditional range fire is probably one of the reasons for increasing bush encroachment.	*A. drepanolobium*, Ethiopia (Terrefe et al. 2011)
**Soil-related**	Erosion, nutrients, salinity, moisture, biology	Intensive cultivation / grazing for subsistence use.	Swidden agriculture identified as driver of forest degradation; decline in soil PH and nutrients with continuous cultivation. Farmers prefer short *C. odorata* fallows to quickly replenish soil nutrients.	*C. odorata*, Ghana (Awanyo 2007)

FIRST-TIER DRIVER CATEGORY: SOCIO-POLITICAL, con't.				
**First-tier type**	**Second-tier sub-types**	**Second-tier sub-type definitions**	**Examples: indirect**	**Case study**
**Livestock-related**	Livestock / grazing increase	External (e.g. State, int'l. development organisation) driven livestock expansion - e.g. by means of policies and policy instruments.	Dutch introduced cattle - expanded to 700,000 head today; cattle trade likely spread *C. odorata*. Invasion leads to loss of grazing resources and movements of livestock into non-invaded land, resulting in land degradation.	*L. camara* and *C. odorata*, Timor (McWilliams 2000)
	Livestock / grazing decrease	Lack/loss of access to grazing land; State or other ban on traditional grazing management and/or erosion of traditional grazing management institutions leading to grazing land degradation and subsequent herd reductions.	Land shortage from population growth, bush encroachment; cattle populations stagnant. Violent conflict forced grazing land abandonment, lack of management increased encroachment. Other savannahs overgrazed, with more encroachment. Encouraged invasive spread and greater livestock movements. Browsers increased greatly in number and importance, where output is mainly destined for meat markets.	Bush encroachment (*Acacia* spp.), *D. viscosa* - E. Pokot, Kenya (case study cluster)
	Overgrazing / overstocking	Lack/loss of access to grazing land/ forage resources (various drivers) while maintaining or increasing livestock numbers for subsistence.	State farms established by evicting pastoralists, reducing grazing land; farms abandoned with regime collapse so invasive escaped. Violent conflict with Issa-Somali pastoralists fuelled by geopolitics; Afar expelled from own grazing land, reducing wet season grazing area, leading to overgrazing, soil degradation, and *P. juliflora* dispersal.	*P. juliflora*, Ethiopia case cluster
**Land-related**	Land abandonment	Forced land abandonment (e.g. through conflict or expropriation); conflict; declining viability of agriculture / grazing.	With apartheid, blacks forced to relocate to remote area, with little infrastructure and employment, resulting in high human and livestock pressures. High soil erosion may be due to crop land abandonment or recovery without rehabilitation; grazing pressure follows.	*E. floribundus*, South Africa (Shackleton and Gambiza 2008)
**Agriculture-related**	Intensification	External introduction (e.g. State, int'l development organisations) of HYVs & external inputs, permanent crops.	Government sees swidden cultivation as cause of deforestation and invasion. Banned it and promoted irrigated rice; invaded dryland rice area rises with area in wet rice. Farmers can't control *Imperata* in dry rice due to excessive labour demands.	*I. cylindrica*, Indonesia (Burkard 2005)
	Shortening of fallow periods	Population pressure combined with land shortage, or unspecified reason for shortening of fallow period.	Fallow periods have shortened due to high population pressure on limited arable land. The positive relationship between cropping intensity and Imperata infestation suggests that intensification contributes to infestation.	*I. cylindrica*, Benin, Cameroon, Ghana, Nigeria (Chikoye 1999)
	Fallow improvement	For subsistence intensification (increase outputs per unit area).	Farmers prefer *C. odorata* fallows to hasten soil regeneration and shorten fallow period.	*C. odorata*, Ghana (Awanyo 2007)

FIRST-TIER DRIVER CATEGORY: SOCIO-POLITICAL, con't.				
**First-tier type**	**Second-tier sub-types**	**Second-tier sub-type definitions**	**Examples: indirect**	**Case study**
**Agriculture-related,** con't.	Displacement of traditional agriculture	Govt' ban on/ discouragement of traditional agriculture through policies and policy instruments, e.g. regarding land use.	Traditional forest-based swiddens are much less susceptible to *Imperata* invasion. Colonialists expanded export crop agricul-ture & intensified food crop agriculture, shortening fallows so that forest-fallows were replaced by *Imperata* invaded fallows. Today, gov't. promotes agricultural intensification, modernization, including alteration or banning of all traditional agriculture, especially that which uses fire.	*I. cylindrica* & *C. odorata*, Indonesia (Dove 1986)
**Forest / tree related**	Forest management	For subsistence goals; State or conservation org. bans on local management, entry.	Invasion promoted indirectly by the Conservation Area Project and community forestry, which reduce human forest interventions that would have controlled invasive species.	*M. micrantha* and others, Kaski, Nepal (Pandey 2017)
	Forest use intensifi-cation; agroforestry	For subsistence; State promotion e.g. of colonisation, plantations, etc.	Farmers restore small bracken areas to produce secondary crops. A third of care for saplings that sprout in the plot, managing natural succession with bananas, pine & cedar to shade out bracken fern.	*P. aquillinum*, Mexico (Berget 2016)
	Forest biodiversity / use decline	Ban on local uses, e.g. swidden ag-riculture, grazing, NTFP collection.	Colonialists designated upper slopes as a forest reserve, pro-hibiting agriculture & greatly restricted land access, forcing farmers to develop a system of semi-permanent cultivation, replacing long fallows with manure & intensive animal husbandry based on *Imperata* fallows.	*I. cylindrica*, Indonesia (Dove 1986 - Javanese)
	Deforestation	For subsistence agriculture, grazing, fuel.	Government road construction promoting colonisation led to immigration, large-scale deforestation & land use intensification, directly causing invasion.	*P. aquillinum*, Mexico (Douterlungne et al. 2010)
**Invasive-related**	Invasive use	Promotion of invasive use by State and non-state actors; subsistence-oriented invasive use.	Introduced by govt' for afforestation and environmental rehabilitation to benefit irrigated state cotton farms & work-ers' settlements; with invasion, promotes 'control through utilisation' - e.g. for woodfuel, charcoal, animal feed, some of which promote spread and environmental degradation.	*P. juliflora*, Ethiopia (case cluster)
**Livelihood-related**	High dependence on wild plant use	Viability of wild plant use for subsistence.	Farmers us on forests for livestock & crops; community forests provide fuelwood, fodder, and thatching. People permitted to enter national park to collect NTFPs & burn parts to encourage regeneration. NTFP collection & *Mikania* use promote spread, reducing NTFPs; now enter Park more than permitted.	*M. micrantha*, Nepal (case study cluster)

FIRST-TIER DRIVER CATEGORY: SOCIO-POLITICAL, con't.				
**First-tier type**	**Second-tier sub-types**	**Second-tier sub-type definitions**	**Examples: indirect**	**Case study**
**Livelihood-related,** con't.	Land pressure	Regulations affecting land access / use; land appropriation for non-subsistence purposes.	Gov't. banned floating garden (water hyacinth based) expansion, establishing a perimeter in the lake; they increased within perimeter through intensification.	*E. crassipes*, Myanmar (Martin 2015)
	Market pressure	State or development organisation promotion of market integration through e.g. policies, taxation, assistance.	Projects promote adoption of legume cover crops and herbicides. Some promote high-input high-output systems with extension, rubber HYVs & clones, chemical inputs, and credit - farmers are obliged to accept the whole package.	*I. cylindrica*, SE Asia (Bagnall-Oakeley et al., stand-ard rubber, 1996)
	Human population pressure	Access to subsistence resources; policies e.g. encouraging new settlement.	Biosphere Reserve declaration meant to help protect the lake but draws even more tourists, so more deforestation, hotels and agriculture to feed more people. Tourism rose to 842m/yr in 2013, promoted by gov't., int'l. designations, unique culture and environment of the Lake.	*E. crassipes*, Myanmar (Martin 2015)
	Labour availability	Population dynamics, health, competing labour demands, labour sharing arrangements.	Swidden system based on *C. odorata* and secondary forest fallows due to lower population density, greater reliance on family labour; producers seek to maximise returns to labour rather than to land.	*C. odorata,* Banjarese short bush fallows, Indonesia (Dove 1986).
	Capital availability	Class, caste, gender, ethnic barriers limiting access e.g. to credit, asset ownership.	Clearing invasive to allow natural grasslands to return would mainly benefit livestock-owning households. Livestock owners mainly male and wealthier. Non-owners benefit from livestock presence, but bear the costs of degradation and depend on the invasive species as a resource.	*E. floribundus*, South Africa (Shackleton and Gambiza 2008)
	Human mobility	Borders, boundaries, State restrictions on mobility, conflict with other resource users / owners, inter-state conflict.	Violent conflict forced grazing land abandonment; lack of management doubled Acacia & reduced grass from 38% in 1973 to 13% in 1978. Other savannahs became overgrazed, giving way to bush encroachment. Encouraged invasive spread, which then contributed to greater livestock movements.	*Acacia* spp., *D. viscosa* – E. Pokot, Kenya (case study cluster)
	Off-farm work, emi-gration	Labour supply, division of labour, social support networks, remittances.	Mainly remittances from international emigration and wage labour that appears to be supporting local livelihoods; people lack resources to combat invasive species.	*M. micrantha* and others, Kaski, Nepal (Pandey 2017)

**FIRST-TIER DRIVER CATEGORY: SOCIO-POLITICAL,** con't.				
**First-tier type**	**Second-tier sub-types**	**Second-tier sub-type definitions**	**Examples: indirect**	**Case study**
**Livelihood-related, con't.**	Social status	Class, caste, gender, ethnic, liveli-hood barriers preventing or limit-ing participation in decision-mak-ing or exercise of certain rights.	State considers that pastoralists degrade land. *P. juliflora* dramatically increases drought vulnerability. Pastoralists consider the loss of land to *P. juliflora* as an autocratic governmental intervention that disregarded their interests.	*P. juliflora*, Ethiopia (case cluster)
**FIRST-TIER DRIVER CATEGORY: TECHNOLOGICAL** The introduction of new technologies (new to the region or locality) or the substitution or extension of existing technologies that induce ecosystem change. Includes the state of scientific and local (i.e. traditional) knowledge, its diffusion and use.				
**Water-related**	Water levels	Hydraulic engineering systems - e.g. irrigation systems including canals, ditches, sluice gates, etc.	Invasive spread through irrigation and other waterways into rice fields. Aquatic habitat planted with rice seedlings is an ideal habitat. Easy rearing methods so readily diffused.	*P. canaliculata*, Asia (Halwart 1994)
	Water nutrients	Inadequate sewage / disposal infrastructure for human popula-tion, activities.	Invasive spread through irrigation and other waterways into rice fields. Aquatic habitat planted with rice seedlings is an ideal habitat. Easy rearing methods so readily diffused.	*P. canaliculata*, Asia (Halwart 1994)
	Drought /water shortage	Hydraulic engineering - irrigation systems (unintended).	Intensive floating garden agriculture, agricultural runoff from catchment areas, and runoff from mines and tourist facilities led to higher nutrient levels and eutrophication, stimulating water hyacinth spread.	*E. crassipes*, Myanmar (Martin 2015)
	Flooding	Hydraulic engineering - irrigation systems (unintended).	Unmanaged irrigation systems led to changes in river course; runs dry due to course changes & flood water irregularity. Irrigation changed seasonal run-off patterns, severely disturb-ing the area and reducing dry-season grazing, leading to over-grazing, greater pastoralist mobility, and *P. juliflora* spread.	*P. juliflora*, Ethiopia (case cluster)
**Vegetation-related**	Fire regime	Intentional use of fire in a predetermined area under specific conditions to manage vegetation regeneration.	Violent conflict forced grazing land abandonment, lack of management doubled *Acacia* and reduced grass. Burning to manage grazing lands, contain encroachment - when grazing land management stops, invasion occurs.	*Acacia* spp., *D. viscosa* – E. Pokot, Kenya (case study cluster)
**Soil-related**	Erosion, nutrients, salinity, moisture, biology	E.g. use of chemicals & fertilisers, excessive water recharge e.g. from irrigation.	Large-scale irrigated agriculture leads to salinization of soils & groundwater, which favour the invasive.	*P. juliflora*, Ethiopia (Müller-Mahn et al. 2010)

**FIRST-TIER DRIVER CATEGORY: TECHNOLOGICAL**, con't.				
**First-tier type**	**Second-tier sub-types**	**Second-tier sub-type definitions**	**Examples: indirect**	**Case study**
**Livestock-related**	Livestock / grazing increase	Use of livestock e.g. for agricultural intensification (draught animal power, manure, transport)	Due to loss of land from conservation efforts, farmers forced to develop a system of semi-permanent cultivation, replacing long fallows with manure and adopting intensive animal husbandry based on cut-and-carry fodder - *Imperata* from fallows and upper slopes.	*I. cylindrica*, Indonesia (Dove 1986 - Javanese)
	Livestock / grazing decrease	Declining productivity of livestock, increasing morbidity/ mortality	Livestock nos. strongly reduced from drought, disease, grazing land loss to Issa, agribusiness, and *P. juliflora* invasion. Many lost with severe drought, as weakened due to lack of feed & water-borne diseases. *P. juliflora* aggravated drought effects - before invasion, sufficient forage even with drought, and seasonal migration for grazing allowed grazing lands to recover - after, different seasonal grazing lands invaded, limiting transhumance, leading to overgrazing and loss of stock.	*P. juliflora*, Ethiopia (case cluster)
	Overgrazing / overstocking	Declining productivity of grazing land while maintaining same or increasing livestock numbers		
**Land-related**	Land abandonment	Declining productivity of agriculture, grazing	Invasion attributed to excessively long cropping period resulting from new rice hybrids and increases in wet rice cultivation that largely displaced traditional mixed crop swiddens - *Imperata* covers abandoned dry fields in one season, productivity reduced dramatically compared to labour input required to weed.	*I. cylindrica*, Indonesia (Burkard 2005)
**Agriculture-related**	Intensification	Through high external input use (e.g. HYVs, synthetic chemicals, machinery & equipment)		
**Forest / tree-related**	Forest use intensification; agroforestry	Roads, infrastructure, machinery to allow intensification	Long history of disturbance in forest reserve from agriculture, road building, quarrying, and bamboo extraction, where state encouraged large-scale commercial bamboo harvesting; led to invasion.	*L. camara,* India (Kannan et al. 2014; Kent and Dorward 2004).
	Forest biodiversity / use decline	Ban on local management, e.g. fire, clearing; promotion of alternative fuels	Bio-gas plants promoted by World Wildlife Fund to reduce fuelwood collection; widely adopted. Bio-gas generated from dung, which requires livestock, which require fodder from forests; grass collection (e.g. for fodder) increases its spread.	*M. micrantha*, Nepal (case study cluster)
**Invasive-related**	Invasive use	Technological change promoting invasive use (e.g. introduction of knowledge, machinery)	Outside promotion of technological change (training, marketing for women materials) increased demand for invasive.	*T. domingensis,* Mexico (Hall 2009)

FIRST-TIER DRIVER CATEGORY: TECHNOLOGICAL, con't.				
**First-tier type**	**Second-tier sub-types**	**Second-tier sub-type definitions**	**Examples: indirect**	**Case study**
**Livelihood-related**	High dependence on wild plant use	Technology /skills for wild plants use.	Inthe had developed unique floating garden (hydroponic) agriculture in late 19th C., became famous worldwide; incorporated water hyacinth as main substrate when invasion expanded.	*E. crassipes,* Myanmar (Martin 2015)
	Land pressure	Technology for land use intensification.	Those with more capital (draught animal power) cultivate close to the village using short Imperata fallows. Land can be cultivated more intensively, requires more capital and more labour, wage labour is available. Those without capital farm more extensively on less valuable land further away.	*I. cylindrica* and *C. odorata*, Banjarese, Indonesia (Dove 1986)
	Market pressure	Promotion of technology, crops, etc. requiring greater market integration.	Road infrastructure, greater market access allowed 'floating garden' agriculture to expand; market liberalisation increased market integration, access to HYVs and chemical inputs, intensification contributed to fishing decline	*E. crassipes*, Myanmar (Martin 2015)
	Labour availability	High labour demands of technology vis a vis the supply of labour.	Wet rice cultivation demands a lot of labour. With labour constraints, farmers prioritize wet rice over dryland cultiva-tion; dryland plots are quickly invaded.	*I. cylindrica*, Indonesia (Burkard 2005)
	Capital availability	Access to and cost of technology.	Improved rubber project systems give better returns on investment but costs are high, and returns are delayed until rubber matures; many are indebted.	*I. cylindrica*, SE Asia (Bagnall-Oakeley et al., standard rub-ber, 1996)
	Human mobility	Transport and transport infrastructure conditions.	Road infrastructure, greater market access allowed 'floating garden' agriculture to expand.	*E. crassipes,* Myanmar (Martin 2015)
	Social status	Social status associated with technology and knowledge employed.	Floating garden agriculture based on water hyacinth promoted as a tourist attraction. Tourism rose, promoted by gov't., int'l. designations, unique culture and environment of the Lake.	*E. crassipes,* Myanmar (Martin 2015)

Table S5. HAIS Framework Drivers: Other Drivers of Invasion and Adaptation by Category and Types. Frequency of mention, frequency percentages, and percent cases reporting

**Driver category**		**Environmental drivers** (n=55, 100%)[1]				**Economic drivers** (n=29, 52.7%)			
**Driver type**	**Driver sub-type**	**Environmental sub-type**	**Freq.**[2]	**Freq. %**[3]	**% cases**[4]	**Economic sub-type**	**Freq.**[2]	**Freq. %**[3]	**% cases**[4]
**Climate**		Climate change, weather	5	7.8	9.1				
**Water related**	Change in water level	Weather, species, abiotic	4	6.3	7.3				
	Water nutrients	Unspecified	1	1.6	1.8	Discharge industries, etc.	1	1.1	3.4
	Drought / water shortage	Climate, biotic, abiotic	3	4.7	5.5				
	Flooding	Unspecified	2	3.1	3.6				
**Vegetation related**	Bush encroachment	Bush:grass ratio weather, fire	2	3.1	3.6				
	Fire regime	Storms, litter accumulation, etc.	5	7.8	9.1				
**Soil related**	Soil degradation, erosion, condition	Water & wind erosion, nutrients, salinity, moisture, etc.	4	6.3	7.3	Intensive cultivation / grazing for commercial use	3	3.3	10.3
**Livestock related**	Livestock / grazing increase					Market-driven livestock expansion	4	4.4	13.8
	Livestock / grazing decrease					Declining commercial profitability of livestock	4	4.4	13.8
	Overstocking/ overgrazing	Unspecified	3	4.7	5.5				
**Land related**	Land abandonment	Unspecified	1	1.6	1.8				
	Land use change					Competing commercial land uses	4	4.4	13.8
	Disturbance	Unspecified	7	10.9	12.7				
**Agriculture related**	Intensification					To increase productivity, market access, profit-seeking	8	8.8	27.6
	Shortening fallow periods					For market intensification	1	1.1	3.4
	Fallow improvement					For market intensification	1	1.1	3.4
	Displacement of traditional agriculture					Market competition, land value increase	1	1.1	3.4

[1]Number and percent of cases reporting the driver category. [2]Frequencies for sub-types refer to the number of times the driver sub-type was mentioned across all cases (see final row of each driver category). [3]Frequency percentages are the proportion that specific sub-type drivers represent of the total of the total sub-type drivers *within*

Table S5. HAIS Framework Drivers: Other drivers of invasion and adaptation by category, type, and sub-type: frequency of mention, frequency percentages, and percent cases reporting (con't.)

**Driver category**		**Environmental drivers**, con't.				**Economic drivers**, con't.			
**Driver type**	**Driver sub-type**	**Environmental sub-type**	**Freq.**	**Freq. %**	**% cases**	**Economic sub-type**	**Freq.**	**Freq. %**	**% cases**
**Forest / tree related**	Forest management					Market intensification	2	2.2	6.9
	Forest use intensification; agroforestry					Market driven, profit seeking	2	2.2	6.9
	Forest biodiversity / use decline	Unspecified	4	6.3	7.3	Declining profitability of forest use	2	2.2	6.9
	Deforestation	Climate, fires, storms, disease	2	3.1	3.6	Commercial agriculture, plantations, ranching, mining, logging	4	4.4	13.8
**Livelihood related**	High dependence on wild plant use	Wild plant abundance, richness, diversity	6	9.4	10.9	Profitability of wild plant use for markets	8	8.8	27.6
	Land pressure	Land resource degradation general	3	4.7	5.5	Cost of land, other factors of production for intensification	4	4.4	13.8
	Market pressure	Unsustainable resource use, general	4	6.3	7.3	Prices, costs, competition drive management decisions	6	6.6	20.7
	Population pressure					*Vis a vis* existing income opportunities	4	4.4	13.8
	Labour availability					Access to / cost of wage labour	7	7.7	24.1
	Capital availability					Lack of access to income, savings, credit, etc.	7	7.7	24.1
	Mobility	Spatial & temporal shifting of population for harvest, use	3	4.7	5.5	Access to means of transport	1	1.1	3.4
	Emigration, off-farm work					Proportion of HH income	6	6.6	20.7
	Social status					Economic marginalisation	3	3.3	10.3
**Invasive use**		Loss /reduction in suitable native species	5	7.8	9.1	Market driven; profitability of invasive use	8	8.8	27.6
**Freq. driver category**			64	16.5			91	23.4	

each driver category. The final row of each driver category presents the percentages of total sub-driver frequencies across all cases. [4]Case percentages are the proportion of cases *within* each driver category that reported that driver sub-type. For categories and definitions, see Tables S4.

Table S5. HAIS Framework Drivers: Other drivers of invasion and adaptation by category, type, and sub-type: frequency of mention, frequency percentages, and percent cases reporting (con't.)

**Driver category**		**Socio-political drivers** (n = 40, 72.7%)				**Technological drivers** (n = 33, 54.5%)			
**Driver type**	**Driver sub-type**	**Socio-political sub-type**	**Freq.**	**Freq. %**	**% cases**	**Technological sub-type**	**Freq.**	**Freq. %**	**% cases**
**Water related**	Change in water level	Management neglect	1	0.7	2.5	Hydraulic engineering	2	2.3	6.1
	Water nutrients					Inadequate sewage infrast.	1	1.1	3.0
	Drought / water shortage	Management neglect	1	0.7	2.5	Hydraulic engineering	1	1.1	3.0
	Flooding	Management neglect	1	0.7	2.5	Hydraulic engineering	1	1.1	3.0
	Fire regime	Restrictions on prescribed burning	3	2.1	7.5	To manage vegetation	8	9.1	24.2
**Soil related**	Soil degradation, erosion, characteristics	Intensive cultivation /grazing for subsistence use	3	2.1	7.5	E.g. use of chemicals & fertilisers, excessive water recharge e.g. from irrigation	1	1.1	3.0
**Livestock related**	Livestock / grazing increase	External-agent driven livestock expansion	3	2.1	7.5	Use of livestock for e.g. agricultural intensification	2	2.3	6.1
	Livestock / grazing decrease	Lack/loss of access to grazing land; forced abandonment; ban on or loss of trad'l. mgmt.	7	4.8	17.5	Declining livestock productivity, increasing morbidity/mortality	4	4.5	12.1
	Overstocking/overgrazing	Lack/loss of access to grazing land/forage resources, increase in livestock for subsistence	4	2.7	10.0	Declining productivity of grazing land	8	9.1	24.2
**Land related**	Land abandonment	Forced abandonment, conflict; declining viability of agriculture/grazing	11	7.5	27.5	Declining productivity of agriculture/grazing	5	5.7	15.2
**Agriculture related**	Intensification	External introduction of HYVs, inputs, permanent crops	4	2.7	10.0	Through high external input use	5	5.7	15.2
	Shortening fallow periods	Population pressure, land shortage, or unspecified	10	6.8	25.0				
	Fallow improvement	Subsistence intensification	8	5.5	20.0				
	Displacement of traditional agriculture	Gov't ban / discouragement of traditional agriculture	7	4.8	17.5				

Table S5. Other drivers of invasion and adaptation by category, type, and sub-type: frequency of mention, frequency percentages, and percent cases reporting (con't.)

**Driver category**		**Socio-political drivers**, con't.				**Technological drivers**, con't.			
**Driver type**	**Driver sub-type**	**Socio-political sub-type**	**Freq.**	**Freq. %**	**% cases**	**Technological sub-type**	**Freq.**	**Freq. %**	**% cases**
**Forest / tree related**	Forest management	For subsistence goals; bans on local management	3	2.1	7.5				
	Forest use intensification; agroforestry	For subsistence goals; externally promoted, e.g. colonisation	6	4.1	15.0	Roads, infrastructure, machinery	1	1.1	3.0
	Forest biodiversity / use decline	Ban on local uses, e.g. swiddens, grazing, NTFP collection	3	2.1	7.5	Ban on local management, e.g. fire, clearing; promotion of alternative fuels	3	3.4	9.1
	Deforestation	For subsistence agriculture, grazing, fuel	5	3.4	12.5				
**Livelihood related**	High dependence on wild plant use	Viability of subsistence wild plant use	4	2.7	10.0	Technology/skills for wild plant use	13	14.8	39.4
	Land pressure	Regulations affecting land access/ use	8	5.5	20.0	Technology for land use intensification	9	10.2	27.3
	Market pressure	External promotion of market integration through policies, taxation, assistance	6	4.1	15.0	Promotion of technology, crops etc. requiring greater market integration	6	6.8	18.2
	Human population pressure	Access to subsistence resources; settlement policies	11	7.5	27.5				
	Labour availability	Population dynamics, competing labour demands	10	6.8	25.0	High labour demands of existing technology	2	2.3	6.1
	Capital availability	Class, caste, gender, ethnic barriers	3	2.1	7.5	Access to / cost of technology	5	5.7	15.2
	Mobility	Borders, boundaries, prohibitions, conflict	3	2.1	7.5	Transport infrastructure conditions	2	2.3	6.1
	Emigration, off-farm work	Labour supply, social support networks, remittances	3	2.1	7.5				
	Social status	Class, caste, gender, ethnic barriers limiting participation or exercise of rights	7	4.8	17.5	Status of technology used	2	2.3	6.1
**Invasive use**		Promotion by external actors; subsistence- use	11	7.5	27.5	Technological change promoting invasive use	7	8.0	21.2
**Freq. driver category**			146	37.5			88	22.6	

Table S6. HAIS Framework Drivers: Local Invasive Impacts. Categories, definitions and case study examples

**FIRST-TIER DRIVER CATEGORY: LOCAL INVASIVE IMPACTS**<br>Direct harms and benefits of the invasives for local human populations, generally related to their resource base and production systems.				
**Second-tier category /definition**	**Third-tier categories**	**Third-tier definitions**	**Example**	**Case study**
**Harmful impacts** – Effects that reduce provisioning, regulat-ing, supporting, and cultural goods and services and / or that increase costs to the local population to achieve these goods and services; effects that increase human morbidity and mortality.	Reduces (useful) native biodiversity	Decreases the abundance or richness of species that are (perceived to be) endemic to the local environment and that provide benefits to local population.	Replaces native species and wildlife habitats, so large mammals move to the core area of the park and tourism declines. Substantially reduces fuelwood and fodder availability.	*M. micrantha*, Nepal (Case cluster)
	Reduces / stops forest succession	Slows or inhibits the succession of vegetation in a (previously) forested site, from bare ground through to mature forest, with vegetation becom-ing taller and more complex over time.	Halts forest succession, so Lancandon Maya farmers devised a way to prevent invasion by planting Balsa trees.	*P. aquilinum*, Chiapas, Mexico (Douterlungne et al. 2010)
	Reduces crop yields (including permanent crops)	Reduces the amount of an agricultural crop (or perineal) harvested per unit of land.	25% of land is less productive with high risk of crop failure. Virtually all farmers reported reduced crop yields, reduced growth & quality.	*I. cylindrica,* Northern Lao PDR, Keoboualapha et al. 2013
	Forces land abandon-ment / reduces availability	Implicated in the unwanted cessation of agricultural or livestock activities on a given land area; creates conditions where some land previously cultivated or grazed can no longer be used for these purposes (e.g. due to excessive labour requirements, soil degradation)	In the largest cooperative (ejido), invaded plots are too large to weed, so are aban-doned; in the more land-restricted cooperative, farmers attempt to purchase access to non-invaded land.	*P. aquilinum*, Southern Yucatan, Mexico (Schnei-der & Geoghegan, 2006)
	Crop depredation by wildlife	Causes wild animals to begin to consume, or increase consumption of, or otherwise damage crops.	Invasion causes reduction in forage, so wild herbivores raid crops.	*L. camara*, India (Sundaram 2012)
	Increases labour re-quirements	Increases the amount of work, especially physical work, to achieve a specific task or goal increases, e.g. per unit of land or output; reduces returns to labour.	Labour requirements for invasive control in dry rice cultivation are excessive, so much of this land is abandoned in favour of wet rice cultivation alone.	*I. cylindrica,* Central Sulawesi, Indonesia (Burkard 2005)

**FIRST-TIER DRIVER CATEGORY: LOCAL INVASIVE IMPACTS**, con’t.				
**Second-tier category & definition**	**Third-tier categories**	**Third-tier definitions**	**Example**	**Case study**
**Harmful impacts**, con’t.	Increases pests	Increases the abundance or type of animals, insects or pathogens detrimental to human concerns.	Thickets may harbour pests; predators can’t shelter & breed in them; indirectly promotes pests by altering water balance & promoting grass weeds in paddy.	*M. pigra*, Cambodia (Rijal and Cochard 2016).
	Increases soil erosion / degradation	Causes a decline in soil condition relative to requirements for human resource (e.g. agriculture) use over time.	Ogan farmers think invasive reduces soil nutrients & prevents water penetration.	*I. cylindrica*, south Sumatra, Indonesia (Dove 1986)
	Reduces grazing land productivity / fodder availability	Causes a reduction in feed availability for herbivores per unit of land; a reduction in dried sources of herbivore feed.	Reduces dry season fodder, already re-duced by land degradation and *L. camara* invasion, leading to even greater grazing pressure & competition for limited feed.	*C. odorata*, Timor (McWilliams 2000)
	Reduces livestock productivity	Causes a reduction in reproductive capacity, growth, and output per head of livestock; an increase in morbidity or mortality that affects output.	Livestock lost in thickets are prey to wild animals; pods and thorns cause jaw dis-ease & dental damage, blindness, lame-ness, and digestive problems increasing morbidity & mortality.	*P. juliflora*, Afar, Ethiopia (case study cluster)
	Increases livestock / human predation	Causes an increase in wild animals killing livestock or humans.	Invasive reduces visibility & blocks forest pathways, increasing wildlife attack on humans and livestock. Some women won't collect NTFPs for cash, preferring lower wages to work in villages.	*L. camara*, India (Kent and Dorward 2014)
	Increased fires / fire risk	Causes an increase in the incidence or probability of accidental fires.	Invasive in areas prone to recurrent burn-ing; increases fires in perennial crops.	*I. cylindrica*, West Africa (Chikoye et al. 2000)
	Fish declines	Causes a reduction in the amount, size or quality of fish procured by harvest.	Fishing declined in part possibly due to in-vasive’s effects on nursing and breeding grounds.	*Eichhornia crassipes*, Myanmar (Martin 2014)
	Reduces mobility (humans / livestock)	Causes a decrease in the ability of a human or livestock population to move freely in geographical space over time.	Human and livestock mobility severely impeded by dense thickets, roads impass-able and tyres punctured; access to river and wells restricted. Access to education, healthcare and social events restricted due to mobility, animal threats and control costs.	*P. juliflora*, Afar, Ethiopia (case study cluster)

**FIRST-TIER DRIVER CATEGORY: LOCAL INVASIVE IMPACTS**, con't.				
**Second-tier categories/definitions**	**Third-tier categories**	**Third-tier definitions**	**Example**	**Case study**
**Harmful impacts**, con't.	Reduces water availability	Reduces the hydrologic capacity of a water source to sustain demand after considering other current water uses and conditions, or reduces access to water sources.	Dense thickets restrict access to lake shores and river banks, thus restricting water supplies for livestock.	*P. juliflora*, Kenya (Mwangi & Swallow 2008)
	Adversely affects human / livestock health	Adversely affects the state of being free from illness or injury of humans or their domestic animal resources.	Invasive is toxic to horses and cattle.	*P. aquilinum*, Southern Yucatan, Mexico (Schneider & Geoghegan, 2006)
**Beneficial impacts** - Increases provisioning, regulating, supporting, and / or cultural goods and services and /or that reduce costs to the local population to achieve these goods and services; effects that enhance human health.	Local economic use	Use of an invasive species for economic gain (income, profit).	Provides fuelwood, fencing, and is a substitute for bamboo and rattan for basket and furniture making – it is abundantly available and can be used with 'zero-investment'.	*L. camara*, India (Kannan et al. 2014, 2016)
	Increases useful biodiversity	Increases the abundance or richness of species that are (perceived to be) endemic to the local environment and that provide benefits to local populations.	50% report that invasive increases other trees and shrubs as it protects seedlings and saplings from livestock browsing.	*O. stricta*, Kenya (Shackleton et al. 2017)
	Provides wood, fuel, fencing	Use of a plant for its porous and fibrous structural tissue found in stems, roots of trees and other woody species for construction, fencing, and fuel.	Extensive use for fuel, ceremonial wood piles and stockade/corral fencing. 94% use it mainly for cooking and heating; 63% collect daily or a few times per week – essential to women and the poor.	*E. florbundus,* South Africa (Shackleton and Gambiza 2008)
	Substrate for hydroponic agriculture	Growth of vegetables without the use of soil, based on plant fibre that provides support.	Incorporated as a principle substrate for iconic 'floating garden agriculture'. Dense mats accumulating in swampy areas are harvested and used for gardens.	*E. crassipes*, Myanmar (Martin 2014)
	Provides livestock feed or forage	Provides food for livestock either through direct grazing or cut and carry or gathering, or after drying or processing.	Intensive animal husbandry is based on cut-and-carry *Imperata* fodder from fallows and volcano's upper slopes.	*I. cylindrica*, central Java, Indonesia (Dove 1986)
	Other subsistence use /food, medicinal	Provides direct use values for supporting human life and needs as medicine, food, fibre, chemicals, etc. (wood, see above).	Eaten by humans.	*P. canaliculata*, Philippines (Joshi et al. 2001)

FIRST-TIER DRIVER CATEGORY: LOCAL INVASIVE IMPACTS, con't.				
**Second-tier categories/definitions**	**Third-tier categories**	**Third-tier definitions**	**Example**	**Case study**
**Beneficial impacts**, con't.	Cultural use / e.g. ritual	Symbolic use of biological organisms for aesthetic, religious, and status-related (e.g. identity) purposes.	Used in witchcraft.	*M. peltata*, Fiji (Daigneault and Brown 2014)
	Labour saving	Reduces the amount of work, especially physical work, to achieve a specific task or goal increases, e.g. per unit of land or output; increases returns to labour.	With *C. odorata* dominated fallows, land is easier to clear and *Imperata* grass is suppressed, so there is less weeding and returns to labour are higher.	*C. odorata,* Banjarese short fallow bush swiddens system, southeast Kalimantan, Indonesia (Dove 1986)
	Suppresses undesired species (e.g. invasives)	Reduces the abundance of plant species that have a harmful effect for human activities.	In experimental plots, *C. odorata* is the most effective at suppressing other weeds and has lowest measured weed biomass.	*C. odorata*, northern Lao PDR (Roder et al. 1995a).
	Reduces predator / pest populations	Decreases the abundance or type of plants, animals, insects or predators whose actions are detrimental to human concerns.	Provides insect control.	*P. cafer*, Fiji (Daigneault and Brown 2014)
	Provides erosion control	Facilitates reduction in the wearing away of topsoil.	Wet rice farmers value grass-cover on slopes close to wet rice fields as it protects against soil erosion.	*I. cylindrica,* Central Sulawesi, Indonesia (Burkard 2005)
	Provides shade	Provides comparative darkness or obscurity protecting species from harmful effects of heat and glare of sunlight.	Provides fencing to contain pigs, which also shades pigs.	*P. aduncum, Papua New Guinea* (Siges et al. 2005)
	Provides windbreaks	A plantation usually made up of vegetation planted in such a manner as to provide shelter from wind and protect soil from erosion.	Planted for windbreaks.	*P. aduncum,* Papua New Guinea (Siges et al. 2005)
	Improves soils / provides green manure	Use and management of vegetation to increase soil fertility; as plants that are incorporated back into the soil.	Improved *Acacia* fallows provide greater soil fertility and green manure.	*Acacia* spp. Madagascar, Congo, and India (Tassin et al. 2012)
	Provides habitat for wild animals	Provides or enhances the environment of a wild animal.	Invasive provides habitat for desirable wild animal species.	*S. campanulata*, Fiji (Brown and Daigneault 2014)

Table S7. HAIS Framework Adaptation Spheres: Invasive Control, Use, and Management. Categories, definitions, and case study examples

FIRST-TIER ADAPATION CATEGORY: INVASIVE CONTROL AND MANAGEMENT<br>Any attempt to prevent or control the growth and propagation of invasive organisms or to manage or use them to beneficial effect				
**Second-tier categories/ definitions**	**Third-tier categories**	**Third-tier definitions**	**Example**	**Case study**
**Manual and mechanical methods** - any physical activity that inhibits unwanted plant growth or makes growing conditions unfavourable, or that encourages desirable plant growth, either selectively or non-selectively.	Pulling and digging	Methods that uproot and remove the unwanted plant from the soil (or partial removal to encourage better growth).	Control - selective weeding, avoid last weeding to enhance *C. odorata* presence; Management - selective thinning of *Acacia* seedlings to encourage growth of larger trees.	*C. odorata*, Lao PDR (Roder et al. 1995a); *Acacia* spp., various, (Tassin et al. 2012)
	Mowing, brush cutting, weed eating	Cutting or shredding above ground to prevent and reduce seed populations and restrict growth (or selective cutting to encourage regeneration).	Control- slashing at ground level w/out removing rootstock - 3/4s in densely invaded and half in less densely invaded areas; Management – harvesters cut stems selectively to use for basket making and never uproot plants.	*C. odorata*, Tanzania (Shackleton et al. 2017); *L. camara*, India (Kannan et al. 2016)
	Mulching	A layer of vegetative material applied to the soil surface to block light, preventing seed germination and growth.	83% saw no mulching benefits compared to current practices.	*C. odorata*, Ghana (Awanyo 2007)
	Tillage	Turning over the soil to damage vegetative parts of plants and expose root systems to desiccation.	Dry soil tillage increases snail mortality.	*P. canaliculata*, Philippines (Joshi et al. 2001)
	Killing (e.g. pests)	Eliminate an organism by causing its death.	Hunting, trapping.	*H. javanicus*, *P. cafer,* Fiji (Brown and Daigneault 2014)
**Cultural control and management** - any activity to modify an organism's growing environment to reduce, increase, or otherwise manage its prevalence.	Competition	Selecting species or varieties that compete with an invasive species for resources.	Planting *Scoenoplectus* clones to compete with *Typha*.	*T. domingensis,* Mexico (Hall 2009)
	Revegetation	Actions to reduce or eliminate an interfering plant problem by maintaining or replanting a mix of vegetation that competes with or suppresses an invasive, or that promotes an invasive.	Control - recovery of abandoned plots through establishment of perennials; Management - farmers collect *Acacia* pods and cover with burnable litter to ensure germination after harvest, or spread seeds in fallow fields.	*I. cylindirica*, Indonesia (Burkard 2005); *Acacia* spp., Madagascar (Tassin et al. 2012)

**FIRST-TIER ADAPATION CATEGORY: INVASIVE CONTROL AND MANAGEMENT**, con't.				
**Second-tier categories/ definitions**	**Third-tier categories**	**Third-tier definitions**	**Example**	**Case study**
**Cultural control and management** con't.	Canopy closure	Managing via planting, pruning, thinning, and leaf and lateral removal to create an upper layer or habitat zone formed by crowns that overlap, forming a virtually continuous layer, to reduce invasive growth or reproduction.	Oil palm planted at high density, generating dense cover in 4-5 years, then felled and cocoa planted with fruit trees and some native trees preserved.	*I. cylindrica*, Cameroon (Jagoret et al 2012)
	Water management	Managing water levels (flooding or drawdowns) affecting soil structure, organic content, seed bank and species composition, usually in wetland environments.	Level land and make channels to collect snails.	*P. canaliculata*, Asia (Halwart 1994)
	Cultivation / grazing	The planting of agricultural crops to eliminate invasives through competition or habitat alteration, or through livestock damage and consumption, or conditioning grasslands to make them more competitive and resistant to invasion.	Plant an annual crop and rubber before *Imperata* re-establishes.	*I. cylindrica*, SE Asia (Bagnall-Oakeley et al 1996)
	Soil management	Manipulating soil condition to provide an optimal chemical, physical and biological habitat for desirable species and a less desirable habitat for harmful invasives, or to enhance environments for invasive reproduction.	*C. odorata* provides a major source of organic matter and soil nutrients in fallows and when slashed and added to soil during land preparation; livestock grazing provides dung and the invasive provides green manure to enhance soils.	*C. odorata*, Ghana (Awanyo 2007); *Acacia* spp., various (Tassin et al. 2012)
**Prescribed burning -** use of fire in a predetermined area under specific conditions to reduce or eliminate an invasive species through direct damage to vegetation and / or destruction of reproductive material.			7-12% use fire to remove excess vegetation before hoe-tillage. 8% clear fields with fire and know it doesn't control *Imperata* effectively but it is cheaper than other methods.	*I. cylindrica,* West Africa (Chikoye et al. 2000)
**Chemical controls** - the use of chemicals that either kill or inhibit the development of invasive species.	Natural toxins or repellents	Poisons or repellents of plant or animal origin found in the local environment that is used to discourage, damage or kill an invasive organism.	Animal fat used to poison *Typha*.	*T. domingensis*, Mexico (Hall 2009)
	Synthetic herbicides	Manufactured chemical substances used to damage or kill invasive plants.	20% use herbicides in crop fields.	*L. camara*, Uganda (Shackleton et al. 2017a)

FIRST-TIER ADAPATION CATEGORY: INVASIVE CONTROL AND MANAGEMENT, con't.				
**Second-tier categories/definitions**	**Third-tier categories**	**Third-tier definitions**	**Example**	**Case study**
**Chemical controls**, con't.	Synthetic pesticides	Manufactured chemical substances used to kill insects or molluscs.	49% use endosulfan, 65% metaldehyde and 15% neonicitinoid insecticides. Initially 98% used endosulfan.	*P. canaliculata*, Ecuador (Horgan et al. 2014)
**Biological controls (BC)** - by means of natural enemies (insects, predators, pathogens)	Native	Use of locally occurring species or pathogens as biological control agents.	Use plant attractants and encourage leeches that eat snails.	*P. canaliculata*, Taiwan (Tsai et al. 2016)
	Introduced	Use of non-native, introduced species or pathogens as biological control agents.	16% of ranchers use biological controls.	*C. solstitialis,* USA (Eagle et al. 2007)
**ICM effectiveness** - degree to which the methods used are successful or unsuccessful at reducing invasive populations to below levels of unacceptable damage or otherwise achieving management goals	Effective	Control method (or combination of control methods) deemed to be effective, i.e. the damaging effects of the populations of the invasive were kept to below what causes unacceptable levels of damage.	Experimental results validated Lacadon method of direct succession, confirming Balsa's potential as a facilitator for restoring degraded tropical forests. Essentially prevents invasion.	*P. aquilinum*, Mexico (Douterlungne et al. 2010)
	Ineffective	Control method (or combination of methods) deemed to be ineffective, i.e. the damaging effects of the invasive were not kept to below what would cause unacceptable levels of damage.	Clearing *Imperata* sheet leaves rhizomes in soil, allowing rapid regrowth. Controls require massive amounts of labour given rapid regrowth, so often neglected and immature rubber suffers from competition and fire damage.	*I. cylindrica*, SE Asia (Bagnall-Oakeley et al. 1996)
	Partly effective /mixed	Mixed effective and ineffective – depending possibly on the different control methods used (some effective, some not), the combination of control methods overall, or the results of a specific control method.	If farmers cannot weed early enough or sufficiently, reduces yields. Livestock owners have no possible means of control on rangelands, and government doesn't attempt to control.	*C. odorata*, Timor (McWilliams 2000)
	May spread	The control method(s) used may result in the spread (greater areal extent) of the invasive.	Mowing does not prevent seed set and may spread seed.	*C. solstitialis*, USA (Aslan et al. 2009)

**FIRST-TIER ADAPATION CATEGORY: INVASIVE CONTROL AND MANAGEMENT**, con't.				
**Second-tier categories/ definitions**	**Third-tier categories**	**Third-tier definitions**	**Example**	**Case study**
**ICM constraints** - restrictions or limits on the ability to implement control efforts or achieve management goals	High labour demands	The control method(s) used require a high quantity of working hours relative to other labour demands; generally lowers returns to labour.	Very labour intensive. 65% of farmers think clearing invaded plots is not worthwhile given labour investment and rather low harvests.	*I. cylindrica*, Indonesia (Burkard 2005)
	Cost	The control method(s) used require high financial outlays (wage labour, equipment, chemical or other purchased inputs) relative to the amount of capital available; lowers returns overall.	Most farmers can't develop sustainable management strategies due to: lack of capital (62.9%), equipment (6.6%), herbicides (2.2%).	*I. cylindrica,* West Africa (Chikoye et al. 2000)
	Impractical to apply	The control method(s) used or available for use are not adapted to local conditions and may interfere with other production goals.	Fallowing/ afforestation impractical due to limited land access. Cover cropping can compete with crops.	*I. cylindrica*, Nigeria (Chikoye et al. 2006)
	Causes collateral damage	Use of control method damages or kills other living organisms or interferes with ecosystem services or human activities	Farmers using pesticides had more problems with caterpillars, viruses and fungal diseases than farmers not using.	*P. canaliculata*, Ecuador (Horgan et al. 2014)
	Lack of knowledge / insufficient application	The control method(s) used or available for use require knowledge (e.g. technical) that local users do not possess; or the control method(s) are applied in a manner that lessens the effectiveness overall.	Spreads across boundaries with high risk of reinfestation. Ongoing infestations when controls delayed. 13% didn't control as not a concern, too costly, no time, or think controls are ineffective.	*C. solstitialis*, USA (Aslan et al 2009)
	Prohibitions	Legal sanctions are imposed if the control method is used.	High use of harmful molluscides until prohibited by the State.	*P. canaliculata*, Ecuador (Horgan et al. 2014)
	Other	Any other constraint to the application of a specific control method or combination of methods.	Efforts to eradicate *Prosopis* are very costly and mainly ineffective. Total eradication is impossible and unsustainable. Government proposals are control through use or biological control, which hasn't been attempted.	*P. juliflora*, Ethiopia (Afar case study cluster)

**FIRST-TIER ADAPATION CATEGORY: INVASIVE CONTROL AND MANAGEMENT**, con't.				
**Second-tier catego-ries/ definitions**	**Third-tier categories**	**Third-tier definitions**	**Example**	**Case study**
**ICM Constraints**, con't.	Unwillingness to adopt effective controls	The perceptions of different groups about whether controls are required, which influences some groups' willingness to adopt effective controls.	Some see no need to control as they have sufficient supply; others are unhappy that it is not controlled because it limits other resources; some say that it never threatened other resources, which were never scarce.	*T. domingensis*, Mexico (Hall 2009)
	Promoted control measures impractical	Perception that controls promoted by ex-ternal or internal agents are not adapted to one's own conditions and / or may in-terfere with one's own production goals.	Farmers reject most government / scientific advice as ill-informed and impractical (e.g. using rice bran as an attractant, which is expensive).	*P. canaliculata*, Taiwan (Tsai et al. 2016)
**FIRST-TIER ADAPTATION CATEGORY: INVASIVE USE AND MANAGEMENT AS A RESOURCE**<br>Incorporation of invasive species use into livelihoods and management of invasive species growing conditions to ensure availability				
**Utilisation of invasive species** - direct use of the invasive for goods and raw materials	For markets	Using the invasive species for commercial purposes - for monetary income or profit.	*Piper* fuelwood preferred over indigenous species, sold for cash.	*P. aduncum*, Papua New Guinea<br>(Siges et al. 2005)
	For own use	Using the invasive species for subsistence (production, consumption, savings).	Substitute missing resources using it for briquettes (65%), fodder (62%), medicine (53%), and green manure (34%).	*M. micrantha*, Nepal (Khadka 2017)
	For complementary activities	Using the invasive as one input (a com-plementary resource) into new activities, e.g. enterprises or production activities.	Used for charcoal production; charcoal is sold daily.	*Acacia* and other fallow species, Madagascar (Tassin et al. 2012)
**Manage invasive as a resource** - actions intended to ensure the required abun-dance (availability) of invasive species for specific uses over time	Encourage reproduction	Actions directed toward increasing the abundance of an invasive.	If natural processes are insufficient for good regeneration, farmers create conditions for fallow invasion by woody legumes.	*Acacia* spp. Madagascar, Congo, India<br>(Tassin et al. 2012)
	Stop or don't imple-ment controls	Ceasing or failing to implement actions intended to reduce the abundance of or eliminate an invasive species.	Stop control in older rice as snails control weeds and do not harm mature rice plants.	*P. canaliculata*, Taiwan (Tsai et al. 2016)

Table S8. HAIS Framework Adaptation Spheres: Resource Use and Management. Categories, definitions, and case study examples

**FIRST-TIER ADAPTATION CATEGORY: RESOURCE USE AND MANAGEMENT**<br>Adaptations to invasive species and other associated drivers that seek to reduce the vulnerability of natural-resource based production systems and to manage, offset, or benefit from the effects, or to reduce reliance on such resources where such responses are not feasible				
**Second-tier catego-ries/definitions**	**Third-tier categories**	**Third-tier definitions**	**Example**	**Case study**
**Cropping systems** - the crops, crop sequences and management techniques used on a particular agricul-tural field over a period of years. It includes all spatial and temporal as-pects of managing an agricultural system.	Move into agriculture	Engage in agricultural production (crops, perennials) when this was pre-viously not an activity.	Many pastoralists moved into agriculture to different degrees at different altitudes as pastoralism became less viable.	*Acacia* bush encroach-ment, *D. viscosa*,<br>E. Pokot, Kenya (cluster)
	Crop cultivars	Within a given crop species, a change in the plants that are selected for de-sirable characteristics that can be maintained by propagation.	Farmers use local rice cultivars that are resistant to snail damage.	*P. canaliculata*, Indonesia (Joshi et al. 2001)
	Crop species	A change in the species of plants grown and harvested extensively for profit or subsistence (species - two in-dividuals can produce fertile offspring).	Many farmers with very low rice yields substi-tute with Job's tears (*Coix lacryma-job*i L.) or maize (*Zea mays* L.), which compete with *Imperata*.	*I. cylindrica,* Lao PDR (Keoboualapha et al. 2013)
	Soil improvement	Actions to reduce erosion, salinity; improve nutrients, moisture, biology.	Distinct advantages in shortened fallows – ability to prevent soil erosion on sloping land after harvest; enhances soil fertility, improving yields and allowing shorter fallows (1995a); enhances soil structure (2001). Suppresses other weedy invasives.	*C. odorata*, Lao PDR (Roder 1995a, 2001)
	Major changes to cropping system	Implementation of a number of changes that substantially alter how a cropping system functions and / or what it produces.	*Imperata* is a major constraint for all crops. Farmers converted *Imperata* grasslands to cocoa plantations, sewing either oil palms or short-cycle annual crops for 2–3 years inter-planted with cocoa, fruit trees and oil palms, where young trees are associated with annual crops for another 2-3 years, until canopy forms.	*I. cylindrica*, Cameroon (Jagoret et al. 2012)
	Major changes to fallow system	Implementation of a number of changes that substantially alter how a fallow system functions and / or what it produces.	*Piper* causes lack of fallow succession; trees are left in fields and coppiced for mulch; ashes used for fertiliser.	*P. aduncum*, Papua New Guinea<br>(Siges et al. 2005)

FIRST-TIER ADAPTATION CATEGORY: RESOURCE USE AND MANAGEMENT, con't.				
**Second-tier catego-ries/ definitions**	**Third-tier categories**	**Third-tier definitions**	**Example**	**Case study**
**Livestock systems** - the livestock, inte-gration of livestock with crops, relation to land and agro-ecological zone, intensity of produc-tion and type of product, including all spatial and temporal aspects of managing a livestock system.	Move into / out of livestock	Engage in livestock production when this was previously not an activity, or cease livestock production.	Farmers with very low yields start rearing livestock.	*I. cylindrica*, Lao PDR (Keoboualapha et al. 2013)
	Livestock numbers	A substantial increase or decrease over time in the number of livestock maintained.	A 49% decrease in number of livestock per household due to invasive's effects on forage, but people still keep livestock.	*M. micrantha*, Nepal (Sullivan et al. 2017a)
	Livestock species	A substantial change in the species of livestock maintained, or in the species composition of the entire herd.	Major shift to small ruminant and camel pro-duction herded near homesteads thus avoiding resource competition, where forage is abundant.	*Acacia* spp., *D. viscosa*, E. Pokot, Kenya (cluster)
	Grazing areas / feed sources	Substantial shifts in movements of livestock for grazing, in grazing areas used, and / or in other sources of livestock feed (e.g. cut and carry, purchases).	Traditional grazing areas became unusable. Graze at greater distances on more marginal lands & in forests. Those using cut & carry systems forced to allow cattle to roam to find forage.	*C. odorata*, Timor (McWilliams 2000)
	Sedentarise	Settling of a nomadic population permanently in one place for a long time.	One adaptation pathway – agro-pastoralism and sedentarisation in areas with market access & employment.	*P. juliflora*, Ethiopia (Afar case study cluster)
**Fishing systems** - the activities, tech-niques, equipment, and spatial and temporal aspects of the act of catching or rearing fish or other aquatic animals.	Fishing intensity	Fishing effort per unit area per unit time.	Catches, average size declined dramatically. Hyacinth may negatively affect fish.	*E. crassipes*, Myanmar (Martin 2014)
	Fishing effort	The amount of fishing carried out, related to the combination of inputs such as the time spent fishing, amounts of hooks or nets used, etc.	Lakeshore invasion of *P. juliflora* prevents fishing.	P. juliflora, Kenya (Mwangi and Swallow 2008)
	Fish species	The species of fish or other aquatic animals caught or reared.	Various species, including some endemics, appear to be near extinction. Hyacinth may negatively affect some species.	*E. crassipes*, Myanmar (Martin 2014)
	Fishing grounds	Geographical areas of fishing activity.	No example found.	

**FIRST-TIER ADAPTATION CATEGORY: RESOURCE USE AND MANAGEMENT**, con't.				
**Second-tier categories/ definitions**	**Third-tier categories**	**Third-tier definitions**	**Example**	**Case study**
**Tree/forest systems** - creation, management, use, conservation, and restoration of forests, woodlands, and trees, associated resources to meet desired goals, including adequate supply and demand.	Move into / out of forestry / agroforestry	Engage in new forest-related activities; cease forest-related activities; engage in agroforestry when this was not previously done.	Farmer development of successful cocoa agroforestry systems on *Imperata* grassland in an area where cocoa production is considered not to be viable.	*I. cylindrica*, Cameroon (Jagoret et al. 2012)
	Demand	Willingness and ability to use a forests/trees and / or the ecological functions these provide.	Demand for NTFPs in Core Park area has increased as has illegal collection. Most residents collect more fodder from Park core than from community forests, private forests, or elsewhere.	*M. micrantha*, Nepal (Murphy et al. 2013)
	Management	Process of controlling use or exploitation of forested land, administrative, economic, legal, and social.	Afar culture discouraged tree cutting & traditional law punished charcoal making. Cutting *P. juliflora* is tolerated & some harvest native species with highly negative effects for ecosystem.	*P. juliflora*, Ethiopia (Ayanu et al. 2014)
	Use	The direct and indirect benefits derived from forests/trees and / or the ecological functions these provide.	Invasive impedes NTFP collection as blocks forest paths; use of NTFPs has declined.	*L. camara*, India (Kent and Dorward 2014)
	Supply	The amount and quality of the direct or indirect benefits derived that are available for use from forests / trees.	Major reduction in NTFPs. Fewer people entering CFs regularly to collect NTFPs (from 98 to 46%) (from 99 to 50%); collection freq. declined from 6 to 1.6 days/wk (6.84 to 3.39); 67% said NTFPS now scarce.	*M. micrantha*, Nepal (Rai and Scarborough, 2015; Khadka, 2017)
	Afforestation	The process of planting large numbers of trees on land which has few or no trees on it.	Converts invaded cropland and fallows into agroforestry systems with many native tree species - 4,846 trees of 67 species inventoried in plantations, fruit = 48%; oil palm 29%, forest trees 24%.	*I. cylindrica*, Cameroon (Jagoret et al. 2012)
	Forest clearing	The process by which vegetation, such as trees and bushes, together with their roots are temporarily or permanently removed.	As land becomes scarcer, there is more need to open invaded land rather than abandon it, or open forest land.	*I. cylindrica*, SE Asia - standard rubber (Bagnall-Oakeley et al. 1996)

<table>
<tr><th colspan="5">FIRST-TIER ADAPTATION CATEGORY: RESOURCE USE AND MANAGEMENT, con’t.</th></tr>
<tr><th>Second-tier categories/ definitions</th><th>Third-tier categories</th><th>Third-tier definitions</th><th>Example</th><th>Case study</th></tr>
<tr><td rowspan="4">Wild resource systems - creation, management, use, conservation, and restoration of (non-forested) areas harbouring wild species or of wild species themselves to meet desired goals.</td><td>Demand</td><td>Willingness and ability to use wild resources and / or the ecological functions these provide.</td><td>Difficult to meet demand for large-stemmed Lantana for furniture making after continuous harvesting for > 10 yrs, reduced density, heavy coppicing causes substantial stem mortality & plant dies if primary stem harvested in wet season.</td><td>L. camara, India
(Kannan et al. 2016)</td></tr>
<tr><td>Management</td><td>Process of controlling use or exploitation of wild resource habitats or species, process of manipulating resources and / or their habitats to ensure desired abundance - administrative, economic, legal, and social.</td><td>Change control and management of existing aquatic plant resource and of invasive, including harvesting techniques, according to changing preferences, demand and supply.</td><td>T. domingensis, Mexico
(Hall 2009)</td></tr>
<tr><td>Use</td><td>The direct and indirect benefits derived from wild resources and / or the ecological functions these provide.</td><td>Trees are planted in Imperata plots and, of the 33 different local plant species that grow in these, 27 are used frequently as fertiliser, construction material, traditional medicine, fodder, fuelwood, etc.</td><td>I. cylindrica, Indonesia
(Burkard 2005)</td></tr>
<tr><td>Supply</td><td>The amount and quality of the direct or indirect benefits derived that are available for use from wild resources.</td><td>A multitude of valuable wild products are obtained from Acacia improved fallows.</td><td>Acacia spp., various
(Tassin et al. 2012)</td></tr>
<tr><td rowspan="2">Conserve / protect threatened species - actions to preserve species with use values that are (or might be, in the absence of the action) threatened by the invasion and its effects on ecosystem processes.</td><td>Management protects or generates useful biodiversity</td><td>Resource use and invasive management are oriented toward generally protecting or encouraging a diversity of species that have direct or indirect use values for humans that are (or might otherwise be) threatened by invasion.</td><td>Greater biodiversity, more valuable trees and no sheet Imperata in jungle rubber systems. Secondary forest regenerates among rubber trees; trees with food or economic value are encouraged. Invasion is effectively prevented.</td><td>I. cylindrica, Indonesia - jungle rubber
(Bagnall Oakeley et al. 1996)</td></tr>
<tr><td>Manage in the wild</td><td>Actions to promote conditions in natural or semi-natural habitats of undomesticated species so ensure a level of abundance that ensures a minimum viable population.</td><td colspan="2">No examples found.</td></tr>
</table>

**FIRST-TIER ADAPTATION CATEGORY: RESOURCE USE AND MANAGEMENT**, con't				
**Second-tier catego-ries/definitions**	**Third-tier categories**	**Third-tier definitions**	**Example**	**Case study**
**Conserve / protect threatened species**, con't.	Transplant / harbour on own land	Moving a threatened species from a natural or semi-natural habitat to an area under one's control where it can be protected, maintained and regenerated.	85% plant trees on private land to replace missing NTFPs from community forests due to invasion.	*M. micrantha*, Nepal (Rai and Scarborough 2011)
**Abandonment -** Cessation of specific forms of land use in a given area.	Agriculture	Cessation of cropping activities in a given area.	Abandoned dryland plots: 7% from *Imperata* infestation; 9% declining soil fertility; 15% distance/steepness; 15% destroyed by pigs; 43% lack of capital / labour to manage invasions; 29% lack of time; 27% management of wet rice plots.	*I. cylindrica*, Indonesia (Burkard 2005)
	Livestock / grazing	Cessation of livestock activities / grazing in a given area.	*C. odorata* is unpalatable, so grazing land unusable; invasion can't be controlled so livestock must be moved to other areas, leading to overstocking and degradation.	*L. camara*, *C. odorata*, Timor (McWilliams 2000)
	Fishing	Cessation of fishing activities in a given area.	Access to the lake for fishing restricted due to *Prosopis* thickets.	*P. juliflora*, Kenya (Mwangi and Swallow 2008)
	Other	Cessation of other land uses (e.g. human settlements) in a given area.	Settlements abandoned due to invasion; some communities also lost many households to emigration.	*P. juliflora*, Ethiopia (Afar cluster)
**Replace reduced/ missing biological re-sources -** using other local resources or non-local sources to substitute those that reduced in abundance or have become otherwise inaccessible relative to demand.	Substitute with other local resources	Using other local biological resources in place of the missing / reduced resource(s).	Fuelwood substituted with dung used in biogas plants.	*M. micrantha*, Nepal (case study cluster)
	Substitute with invasive	Using the invasive as a substitute for one or more of the reduced / missing biologi-cal resources.	Most resources lost when forest succession was halted by invasive are substituted by use of the invasive.	*P. aduncum,* Papua New Guinea (Siges et al. 2005)
	Substitute through trade	Trading (cash, in kind, etc.) to obtain substitutes the reduced or missing resources.	Purchase fuelwood/fodder in local markets.	*M. micrantha*, Nepal (case study cluster)

Table S9. HAIS Framework Adaptation Spheres: Household Adaptation. Categories, definitions, and case study examples

**FIRST-TIER ADAPTATION CATEGORY: HOUSEHOLD ADAPTATIONS** Changes in the use and organisation of labour and capitals for invasive control and management, and related resource management adaptation within a household (a small group of persons who share the same living accommodation, who pool some, or all, of their income and wealth and who consume certain types of goods and services collectively, mainly housing and food)				
**SECOND-TIER CATEGORY: HOUSEHOLD LABOUR ORGANISATION** Changes in the social organisation of labour: demand, supply, divisions, both productive and unproductive, for invasive control and resource management adaptation				
**Third-tier categories/definitions**	**Fourth-tier categories**	**Fourth-tier definitions**	**Example**	**Case study**
**Source of labour for invasive control and management** - the people engaged in or available for work entailed in controlling or managing invasive species.	Own or household labour	Work is supplied by the labour of the person or persons directly engaged in the productive activity affected by the invasive or by members of that person's household.	Avg. of 22 days/ ha to clear *Imperata* (24 for forest clearance), but soil fertility is lower. Perennials require less labour and suffer less degradation if abandoned temporarily.	*I. cylindrica*, Indonesia (Burkard 2005)
	Communal labour	Work is shared by some or all members of a community through different arrangements, e.g. labour exchange, collective labour.	Households organise collective removal efforts with neighbours, which are the only collective efforts that exist - local governance bodies and the State doesn't attempt to control.	*M. micrantha*, Nepal (Sullivan et al. 2017a)
	Wage labour	The socioeconomic relationship between a worker and an employer, where the worker sells his or her labour under a formal or informal employment contract. These transactions usually occur in a labour market where wages are market determined.	Where invasion is dense, 42% pay to clear fields; where less dense, 21% pay. Wages higher in dense areas - avg. of US$ 550/yr versus US$ 340/yr in less dense areas.	*C. odorata*, Tanzania (Shackleton et al. 2017)
**ICM - related labour constraints** - restrictions or limits on the ability to implement ICM efforts related to labour demand or supply.	Labour intensive	Requiring a large workforce or a large amount of work relative to output.	All use hand-pulling - time consuming. Most hoe weed; slashing is fast and immediately controls, but must be done frequently, as *Imperata* returns. 20% dig out rhizomes; very effective but labour intensive.	*I. cylindrica*, Nigeria (Chikoye et al. 2006)

**FIRST-TIER ADAPTATION CATEGORY: HOUSEHOLD ADAPTATIONS**, con't.				
**SECOND-TIER CATEGORY: HOUSEHOLD LABOUR ORGANISATION**, con't.				
**Third-tier cate-gories/ definitions**	**Fourth-tier categories**	**Fourth-tier definitions**	**Example**	**Case study**
**ICM – related labour constraints**, con't.	Demand significant as a proportion of total workload	The quantity (in time) of work required is determined to represents a relatively large proportion of total workload (e.g. for all productivity activities combined, or for a specific set of productive activities, e.g. cropping).	24 days/yr avg. to control, reduces productivity - represents a significant proportion of total workload.	*S. campanulata*, Fiji (Brown and Daigneault 2014)
	Demand is a major constraint to control / management	Demands for work are so high relative to supply that this impedes effective control or management.	Very high labour demands for control and recovery, often not available if farmers are also engaged in wet rice production.	*I. cylindrica*, Indonesia (Burkard 2005)
	Reduces productivity / returns to labour	The proportionate gain in output from controls is less than the expansion of the labour force; output per worker declines.	Weeding requirements lower returns to labour.	*C. odorata*, Lao PDR (Roder, 1995a, 1995b)
	Labour demand varies widely	The amount work required has a large range depending on various other factors, e.g. degree of invasion, type of cropping system.	Increases weeding time 1- 40 days/yr – per ha. mean 11 ± 9; median 7 d/y/ ha; those with more than 1 crop and those with fallows weed more, with larger holdings weeded less.	*M. pigra*, Cambodia (Rijal and Cochard 2016)
	No constraints, or low labour requirements	No restrictions or limits due to the amount of work required as labour requirements are low relative to the demand and supply of labour generally.	High labour demands are a major constraint for clearing and weeding in standard rubber systems but in jungle rubber systems, labour presents no constraint as invasion is effectively prevented.	*I. cylindrica*, SE Asia, Indonesia (Bagnall-Oakeley et al. 1996)
**Other change in social organisation of labour for adaptation** - associated with harms & benefits or resource management adaptation	Travel time	Time spent in traveling while working, or from the place of residence to the place of work, or to engage in other activities.	Increase in travel time and distance to find NTFPs. Grass collection: from 1VDC travel an average of 8.4 km, from another avg. of 5.9 km. Across VDCs, travel furthest for thatch grass and shortest for fuelwood.	*M. micrantha*, Nepal (Murphy et al. 2013)
	Productive time	Time spent in work that produces an output or other direct benefit.	Less time is spent on land clearing as *Piper* fallows are easy to clear; returns to labour are higher.	*P. aduncum*, Papua New Guinea (Siges et al. 2005)

**FIRST-TIER ADAPTATION CATEGORY: HOUSEHOLD ADAPTATIONS**, con't.				
**SECOND-TIER CATEGORY: HOUSEHOLD LABOUR ORGANISATION**, con't.				
**Third-tier categories/ definitions**	**Fourth-tier categories**	**Fourth-tier definitions**	**Example**	**Case study**
**Other change in social organisation of labour**, con't.	Unproductive time	Time spent in work that would otherwise not be spent in absence of the invasion; time that yields no additional output.	Amount of fodder & fuelwood collected per trip declined by more than half (57% collected 50-75 kg fuelwood, now down to 1.2%); 46% saw no reduction.	*M. micrantha*, Nepal (Rai & Scarborough, 2015; Khadka, 2017)
	Household labour divisions	The assignment of different work responsibilities to different members of a household usually depending on age, sex, kinship and relationship with the household head.	Women now sell fuelwood which provides an independent source of income, and can prepare land (previously a male activity) as Piper makes it easy to clear.	*P. aduncum*, Papua New Guinea (Siges et al. 2005)
**SECOND-TIER CATEGORY: HOUSEHOLD CAPITALS**<br>Changes in supply of financial (e.g. credit, savings) and physical (equipment, tools, buildings, livestock) assets required for livelihood activities, especially production				
**Capitals for invasive control and management** - physical and financial assets required for invasive control and management.	Non-labour inputs	Financial and other outlays for equipment, chemicals, or other consumables used for control, excluding labour.	*Imperata* system requires capital outlays e.g. for cattle ploughing; *C. odorata* system does not.	*I. cylindrica*, *C. odorata*, Indonesia, Banjarese (Dove 1986)
**Capital-related control constraints for control and management** - restrictions or limits on the ability to implement control or management efforts related to access to physical and financial capital.	Lack money to control	Producers lack access to the financial capital (e.g. savings, cash, credit) to pay for wage labour, equipment, chemicals, etc. required to effectively control the invasive.	Herbicides - 20% spend about $US500 / yr but most can't afford herbicides or wage labour for control.	*L. camara*, Uganda (Shackleton et al 2017a)
	Financially draining	Producers have financial capital but outlays cause financial stress or hardship for livelihoods or for the production system, making it economically less viable / profitable or risky, possibly leading to abandonment.	Controls require a mean annual expenditure of US$1247. Can be financially draining as must be done over a number of years.	*C. solstitialis*, USA (Eagle et al. 2007)

FIRST-TIER ADAPTATION CATEGORY: HOUSEHOLD ADAPTATIONS, con't.				
**SECOND-TIER CATEGORY: HOUSEHOLD PRODUCTION**<br>Changes in what is put into a productive process or system and in what is produced (output) by household members from productive resource use and change in off-farm productive activities				
**Third-tier cate-gories/ definitions**	**Fourth-tier categories**	**Fourth-tier definitions**	**Example**	**Case study**
**Resource inputs/outputs** - overall effects (from invasive control/management and adaptation) on what is put into a productive process or system and what is produced (output), excepting labour.	Crops	Non-labour related inputs and outputs related to agriculture / cropping systems.	Cocoa plantations intercropped with oil palm and other valuable trees -marketable cocoa yield similar to gallery forest plantations & and close to yields in forest areas in C and S Cameroon.	*I. cylindrica*, Cameroon (Jagoret et al. 2012)
	Livestock	Non-labour related inputs and outputs related to livestock systems.	In past year on avg. 9 goats, 7 sheep, and 15 cattle lost per household; livestock weight loss, reduced milk yields.	*O. stricta*, Kenya (Shackleton et al. 2017)
	Fishing	Non-labour related inputs and outputs related to fishing or aquaculture.	Fish reduction from pesticide use.	*P. canaliculata*, Asia (Halwart 1994)
	Income	Non-labour related inputs and outputs of activities that generate money, especially on a regular basis, in relation to overall production systems (e.g. crops, livestock, fishing) combined when these are commercially oriented.	Lost cattle; lost forage value + control costs = 7-16% of total pasture revenue.	*C. solstitialis*, USA (Eagle et al. 2007)
	Other	Non-labour related inputs and outputs related to other resource-based productive activities, e.g. NTFP collection and management.	Supply of NTFPs has declined overall.	*L. camara*, India (case study cluster)
**Off-farm productive activities** - activities away from farms/places of settlement to earn income or other means of living.	Local wage labour	Engagement in off-farm wage labour that doesn't require a change of residence and occurs on a non-seasonal (i.e. permanent, temporary) basis.	Leads to decline in other activities such as cropping, grazing, and NTFP collection, but may yield higher incomes given effects of the invasion.	*L. camara*, India (Kent and Dorward 2014)

FIRST-TIER ADAPTATION CATEGORY: HOUSEHOLD ADAPTATIONS, con't.				
**SECOND-TIER CATEGORY: HOUSEHOLD PRODUCTION**, con't.				
**Third-tier categories/ definitions**	**Fourth-tier categories**	**Fourth-tier definitions**	**Example**	**Case study**
**Off-farm productive activities**, con't.	Local enterprises	Engagement in a business near the place of residence / farm that may or may not employ a small number of workers and does not have a high volume of sales; usually privately owned and operated sole proprietorship.	Enterprises such as charcoal production bring substantial additional income.	*Acacia* spp. Madagascar, Congo, India (Tassin et al. 2012)
	Seasonal migration	Engagement in movements for paid work or to track resources for only part of a year because the work performed for income or production depends on seasonal conditions.	Some pastoralists seasonally migrate for work; pastoralists must migrate further and for longer periods for grazing.	*P. juliflora*, Ethiopia (Afar case study cluster)
	Emigration	Leaving one's own country to take up paid work or engage in an enterprise; part of the income earned may become household income in the form of remittances.	There is substantial emigration of youth for foreign employment as households lack resources to cope with climate change and invasions.	*M. micrantha* and various others, Nepal (Pandey 2017)

Table S10. HAIS Framework Adaptation Spheres: Social-ecological Relations and Human Well-being. Categories, definitions, and case study examples

**FIRST-TIER ADAPTATION CATEGORY: SOCIO-ECOLOGICAL RELATIONS AND WELL-BEING**<br>Social-ecological refers to mutually conditioning relations between humans and between humans and nature, and well-being refers to human material living conditions, quality of life, and sustainability				
**Second-tier catego-ries/definitions**	**Third-tier categories**	**Third-tier definitions**	**Example**	**Case study**
**Micro-level socio-ecological relations and well-being** - change in small-scale social-ecological relations and well-being of individuals, households, and other small social groups.	Social stratification	Changes in the ranking of individuals, households, or other social groups in an enduring hierarchy of status based on socioeconomic conditions, or factors such as age, sex, or ethnicity.	Those who lost most livestock had less ability to diversify than those who did not. Three groups - 1) those who sedentarised & became agro-pastoralists; 2) those who diversified income; 3) those who maintained nomadic pastoralism. Women have massively increased workloads; female-headed HH most destitute. Clan leaders enriched by land leasing, labour contracting for *P. juliflora* control for foreign agribusiness.	*P. juliflora*, Ethiopia (Rettberg and Müller-Mahn 2012)
	Subsistence production	The production of direct use values for supporting human life and needs.	Clearing invasive to allow natural grasslands to return mainly benefits livestock-owning HHs. Livestock owners mainly male and wealthier. Non-owners bear costs of degradation & removal of a key species. Negative impacts removal mainly felt by women who collect wood.	*E. floribundus*, South Africa (Shackleton and Gambiza 2008)
	Income /savings	Income - money received on a regular basis, for work, through production or investment; savings - excess of income over consumption; production put by as illiquid assets to smooth or buffer consumption & permit investment.	In India, *Acacia* fallows are a savings account; trees are harvested in bulk at the end of the fallow cycle to pay major expenses.	*Acacia* spp., India (Tassin et al. 2012)
	Entrepreneurship	Establishment or performance of a business that generates self-employment or profit.	60% of pastoralists are involved in other income-generating activities due to livestock loss. > 90% earn from wage labour on cotton farms, outmigration, fuelwood & mat sales - before only in emergencies. Poor HH settled near large farms or urban areas sell sheep & goats & up to 4 non-pastoral activities - most are low paid and temporary. Charcoal trade dominated by immigrants; most benefits accrue to charcoal owners.	*P. juliflora*, Ethiopia (Afar case study cluster)
	Employment	Having paid work, conditions of paid work.		

FIRST-TIER ADAPTATION CATEGORY: SOCIO-ECOLOGICAL RELATIONS AND WELL-BEING, con't.				
**Second-tier categories/definitions**	**Third-tier categories**	**Third-tier definitions**	**Example**	**Case study**
**Micro-level socio-ecological relations and well-being**, con't.	Poverty	A state characterised by any or all of the following conditions: lack of income and productive resources to ensure sustainable livelihoods; hunger and malnutrition; ill health; limited or lack of access to education and other basic services; increased morbidity and mortality from illness; homelessness and inadequate housing; unsafe environments and social discrimination and exclusion.	Cumulative effects of changed flood regimes and *P. juliflora* invasion in 1990s tipped the Afar into a state of chronic food insecurity and impoverishment. Sharing of milk, the most important & culturally significant resource, is restricted due to low supplies which 'diminishes their way of life and corrupts and impoverishes their culture, ultimately transforming their i dentity' (Rogers et al 2017, 8).	*P. juliflora*, Ethiopia (Afar case study cluster)
	Food security	When people, at all times, have physical, social and economic access to sufficient, safe and nutritious food to meet dietary needs for a productive and healthy life.		
	Vulnerability	The inability of people to withstand adverse impacts from multiple stressors to which they are exposed; the relative degree of exposure to damage or harm.	Pastoralism...has been demonstrated to effectively coexist with local environments and to support the maintenance of areas rich in biodiversity. Whilst the alternatives, mono-cropping, commercial plantations and small-scale cash crops, fail to offer the same level of environmental protection, with no incumbency upon users to preserve the unique ecosystem, they do offer the prospect of better confronting the invasion, a fact which offers the most significant threat to pastoralists. The tragedy of the invaded commons is that all of the co-evolved, ecological sensitivity and specialism is a burden rather than a boon.'	*P. juliflora*, Ethiopia (Rogers et al 2017, 10)
	Dependency	A state of relying on or being controlled by someone or something else.	On the state for solutions to grazing land invasion and resource competition, which are not forthcoming.	*C. odorata*, Timor (McWilliams 2000)

FIRST-TIER ADAPTATION CATEGORY: SOCIO-ECOLOGICAL RELATIONS AND WELL-BEING, con't.				
**Second-tier catego-ries/definitions**	**Third-tier categories**	**Third-tier definitions**	**Example**	**Case study**
**Micro-level socio-ecological relations and well-being**, con't.	Security	Being free from danger or threat, e.g. of physical harm.	To herd camels & cattle at distance - must purchase weapons for young herders – but young men more unwilling to assume risks. Clans leasing land to foreign agribusiness helps establish territorial claims against Issa. Charcoal workers risk wild animal attacks, must be protected by other men; increasing incidence of rape & murder of women by charcoal workers.	*P. juliflora*, Ethiopia (Afar case study cluster)
	Health	The state of being free from illness or injury.	Thorns wound limbs & eyes, cause blindness, disability, and amputations due to infection. Access to healthcare diminished due to impeded mobility and costs of *Prosopis* management. Decreasing water quality poses threats to health.	*P. juliflora*, Ethiopia (Afar case study cluster)
**Meso-level socio-ecological relations** - change in social-ecological relations falling between the micro- and macro-levels, such as a community or an organization, land-scape or ecosys-tem; analyses spe-cifically designed to reveal connections between micro- and macro-levels.	Social institutions / governance	Social institutions - established sets of norms and subsystems that support a society's survival; Governance - an or-ganization or mechanism that formally or informally guides the decision-mak-ing process and sets into motion the different actors and apparatuses in the implementation process, where the State is generally the main actor.	Charcoal 'elite' is gaining influence & undermining cus-tomary institutions in natural resource management. Communal livestock management has eroded - herders make grazing & pasture management decisions individu-ally. Before, when HHs lost livestock the clan divided risks & provided means to restock; now this is rare, as risks are very high due to *Prosopis*.	*P. juliflora*, Ethiopia (Afar case study cluster)
	Settlement / land use patterns	The spatial distribution of places where people live and interact, and the spatial distribution of land by func-tions.	*Piper* allows new areas to be cleared for settlement; smaller, more isolated hamlets are created by close kin.	*P. aduncum,* Papua New Guinea (Siges et al. 2005)

FIRST-TIER ADAPTATION CATEGORY: SOCIO-ECOLOGICAL RELATIONS AND WELL-BEING, con't.				
**Second-tier categories/definitions**	**Third-tier categories**	**Third-tier definitions**	**Example**	**Case study**
**Meso-level socio-ecological relations**, con't.	Resource tenure	Legal or customary relationship between individuals or groups with respect to natural resources; how entitlements are allocated within societies.	Invasions support government classification of land as 'empty' and 'unowned' and therefore subject to appropriation for 'development' without compensation.	*I. cylindrica* and *C. odorata*, Indonesia (Dove 1986)
	Knowledge	Facts, information, and skills acquired through experience or learning; theoretical or practical understanding of a subject.	Long-term management of an ecosystem creates sophisticated knowledge of forest dynamics. 'there have been few attempts to convert this rich source of traditional ecological knowledge into scientifically validated tools for ecological restoration' (p. 322).	*P. aquillinum*, Mexico (Douterlungne et al. 2010)
	Marginalisation	Treatment of a person, group, or concept as insignificant or peripheral.	Blacks forced to relocate to the area under apartheid. Women and poor don't participate in workshops on invasive control; dominated by powerful male livestock owners. No critical detailed assessment of extent or intensity of degradation caused by invasion, or its causes or consequences for land dynamics and livelihoods.	*E. floibundus*, South Africa (Shackleton and Gambiza 2008)
**Conflict** - a struggle or clash between opposing forces; state of opposition between ideas, interests, etc; disagreement or controversy, may be violent; occurring at micro or meso-scales.	Over land	With respect to competing functions of land, land management, scarcity, and tenure.	At times violent; over grazing and homestead land as pastoralists forced to seek land, grazing resources and water in others' territories. Greater conflict will occur without invasive control and adaptation to rapidly evolving change processes.	*P. juliflora*, Baringo-Bogoria lakes (Kenya case study cluster)
	Over resource rights	With respect to competing functions of natural resources (biotic and abiotic, including e.g. water), management of such resources, scarcity, and tenure.	*Prosopis* charcoal trade generating conflicts over the distribution of costs & benefits between pastoralists & charcoal owners; conflicts with charcoal producers who are seen as exploitative outsiders & with NGOs that promote inappropriate utilisation strategies. Conflicts exist between charcoal owners & producers, and between clans, charcoal makers, and communities.	*P. juliflora*, Ethiopia (Afar case study cluster)
	Over invasive control and management	With respect to attempts to prevent or control the growth and propagation of invasive organisms, or to manage them for beneficial ends.	Conflict between Community Forest members and an NGO over perception that *Mikania* is not a problem. No efforts on the part of any government entity, NGO or village development or community forest committee to control; all collective efforts are organised by HHs. Distrust between members and committee leaders.	*M. micrantha*, Nepal (Sullivan et al 2017a, 2017b)

Table S11. HAIS Framework Adaptation Types, Pathways, Feedbacks, and Social-ecological Outcomes. Categories, definitions, and case study examples.

**FIRST-TIER ADAPTATION CATEGORY: ADAPTATION TYPES**<br>Adaptation types refers to social processes and strategies that have common characteristics, and that have been identified as occurring across different cultures and time periods in response to environmental change.				
**Second-tier category & definition**	**Third-tier categories**	**Third-tier definitions**	**Example**	**Case study**
**Mobility** – Movement to avoid risk or pool resources, or in search of natural resources or better circumstances.	Resource tracking	Human movements associated with change in natural resource availability across space and time; often humans move with their assets (e.g. livestock, equipment, dwellings).	Due to invasion, people must travel further to collect NTFPs, often entering the Core Park area illegally to collect.	*Mikania micrantha*, Nepal, Murphy et al. 2013
	Migration	Movement of people from a locality of origin to a destination place across some predefined, political boundary, temporarily or permanently, not associated primarily with resource tracking.	Young men temporarily out-migrate for wage labour in nearby quarries: push factor is *Lantana* invasion in agriculture, pull factor is higher income from wages than from agriculture.	*Lantana camara,* India, Kent & Dorward 2014
	Resettlement	The transfer of a substantial group of people from one dwelling site to another, usually for a lengthy period of time.	*Prosopis* forced people out of original settlements, amalgamating people and livestock from different locations and clans.	*Prosopis juliflora*, Afar, Ethiopia, Hamedu 2014
	Sedentarisation	Settling of a nomadic population.	Bush encroachment and conflict, development projects, encouraged people to settle and adopt agriculture.	Bush encroachment, Kenya, Greiner and Mwaka 2016
**Diversification** – an increase in the number of components of a production or livelihood system (diversity), and changes in the organization of that diversity in space and time.	Ecological	Temporal and spatial: increasing the number or type ecological niches, species or varieties used, or planting, harvesting, hunting, fishing times, to accommodate fluctuations or improve circumstances, enhance flexibility.	Farmers substitute more competitive crops, introduce perennials, move into livestock production.	*Imperata cylindrica*, Lao PDR, Keoboualapha et al. 2013
	Subsistence	Increasing the number of components of a livelihood system to enhance flexibility and subsistence.	Farmers begin to cultivate minor crops and perennials on reclaimed bracken fern land.	Bracken fern, Oaxaca, Mexico, Berget et al. 2015
	Wage labour	Exchanging labour power for cash or other remuneration; income can be used to substitute for non-wage goods and services.	To control golden apple snails, women and small children collect 1 large can for about US$ 0.50.	Golden apple snail, Ifugao, Philippines, Joshi et al. 2001

**FIRST-TIER ADAPTATION CATEGORY: ADAPTATION TYPES**, con't.				
**Second-tier categories/ definitions**	**Third-tier categories**	**Third-tier definitions**	**Example**	**Case study**
**Diversification,** con't.	Enterprises	Engaging in the production of goods or services for market exchange, where income can be used to substitute for non-wage goods and services.	Women sell *Piper* fuelwood.	*Piper aduncum*, Papua New Guinea, Siges et al. 2005
**Asset (re)allocation** - the distribution of access, control, management, and associated risks and benefits of assets, within and between individuals and social groups.	Pooling	Increasing sharing control or management of assets and resources (wealth, labour, knowledge) across individuals or social groups.	Agropastoralists provide grain and *Prosopis* control in exchange for milk and protection against Issa from mobile pastoralists.	*Prosopis juliflora*, Afar Ethiopia, Müller-Mahn & Rettberg 2012
	Individualisation	Moving toward individualism and self-reliance, where risks, property, management, goods, or services are transferred from groups to individuals, and where individual interests achieve precedence over that of a social group.	Privatisation of land rights with conversion to cacao agroforestry.	*Imperata cylindrica*, Cameroon, Jagoret et al. 2012
**Rationing -** Extend the supply of resources by controlling their circulation and consumption over time and space. Storage is among the most basic forms of rationing.			Not found.	Not found
**Shifts in species or varieties** - changes in the use of specific species, specie's parts, or varieties that can form part of any of the above adaptation types.	Substitution	Use of one species or variety, or its products, to substitute another.	Pastoralists shift from grazers to browsers.	Bush encroachment, Kenya, Österle 2008, Vehrs 2016
	New uses	A species, variety, or specie's product that was previously not present or known is used for purposes that fulfil pre-existing production/ consumption needs, or for innovation.	Invasive is hunted for food and for control	Small Indian mongoose, Fiji, Brown and Daigneault 2014
	Market sources	Species or species products that cannot be accessed or are otherwise too costly to use are sourced through cash payments. May involve human biological corridors.	Feed and fuelwood declines lead to market purchase.	*Mikania micrantha*, Nepal, Rai and Scarborough 2013

**FIRST-TIER ADAPTATION CATEGORY: ADAPTATION TYPES**, con't.				
**Second-tier category & definition**	**Third-tier categories**	**Third-tier definitions**	**Example**	**Case study**
**Resource use intensity -** practices that change productivity by changing inputs per unit, or ecosystem properties, or the temporal frequency of use, increasing or reducing output per unit**.**	Intensification	Increasing productivity by changing inputs, ecosystem properties, or temporal frequency of use, increasing output per unit.	Shift to cacao/oil palm, greater tree diversity through agroforestry.	*Imperata cylindrica*, Cameroon, Jagoret et al. 2012
	Disintensification	Decreasing productivity by changing inputs, ecosystem properties, or temporal frequency of use, decreasing output per unit.	Grazing land abandonment.	*Chromolaena odorata,* Timor. McWilliam 2000
	Both	Combination of strategies e.g. to produce different goods and services in the same area, to increase resilience or maintain a basket of outputs per unit while reducing non-land inputs.	Wet rice plot intensification, dry rice plot abandonment due in part to high labour requirements for control, planting perennials for cash, use of communal invaded plot biodiversity.	*Imperata cylindrica*, Indonesia. Burkard 2005
**Innovation -** New, unplanned method or technique that arises to address a certain need...does not refer to applying existing strategies used by a minority.			Unknown - many probably represent the extension of pre-existing practice.	
**Revitalisation** - organized or individual actions to reduce stress, improve livelihoods, and counter loss of biotic resources.	Cultural/institutional	Organized reconfiguration of ideology and institutional practices to reduce stress and create a more satisfying culture, by reviving historical practices, beliefs, or knowledge (see also Asset (re-)allocation).	None; the opposite occurred in Afar, Ethiopia, where cultural institutions have broken down.	*Prosopis juliflora,* Afar Ethiopia. Müller-Mahn & Rettberg 2012
	Conservation	Protection of species or habitats, including biodiversity more generally.	Lacandon Maya swidden cultivators planted balsa trees to shade out bracken fern, preserving long fallow succession and associated diversity.	Bracken fern, Chiapas, Mexico. Douterlungne et al. 2010
	Restoration	Systems that have become unstable due to positive feedbacks are returned to resilience.	*Acacia* fallows are managed to produce multiple economically important products, green manure and fodder.	Multiple locations. Australian *acacia*. Tassin et al. 2012.

**FIRST-TIER ADAPTATION CATEGORY: ADAPTATION PATHWAYS**			
Adaptation pathways are combinations of adaptation types pursued by different social groups over time and space that are conditioned by cultural, political, economic, environmental, and development contexts and by individual and group knowledge, values, livelihood assets and strategies, resource systems and niches, which are influenced by historical pathways. Create and are subject to feedbacks.			
**Example**			**Case study**
Indigenous Banjarese swidden farmers. Those with more capital and access to wage labour cultivate close to the village, use short *Imperata* fallows. Those with less capital and only family labour cultivate further from village, more extensively, using *Chromolaena* improved fallows.			*Imperata* and *Chromolaena odorata*, Indonesia. Dove 1986.
**FIRST-TIER CATEGORY ADAPTATION: ADAPTATION FEEDBACKS** Feedbacks are the effect that change in one part of a system has on another and how this then affects the source of the change inducing more or less of it. Form the basic dynamics for regulating the state of the social-ecological system.			
**Second-tier categories**	**Second-tier definitions**	**Example**	**Case study**
**Positive feedbacks**	Amplify processes that destabilise systems.	Pesticides cause collateral damage and harm native predators, possibly increasing invasion.	Golden apple snail, Asia, Horgan et al. 2014
**Negative feedbacks**	Reduce fluctuations and stabilise important drivers.	Management of *acacia* in fallows increases biodiversity, useful species, income and well-being.	*Acacia* spp., Tassin et al. 2012
**Feedback loops**	Occur when social and ecological feedbacks are mutually reinforcing, leading to possible regime shifts.	Dung-based bio-gas stoves introduced to decrease fuelwood demand increased demand for grass to feed livestock, grass collection can disperse invasive. *Mikania* invasion decreased fuelwood and grass resources, so people travelled further to collect them. This travel, and people's efforts to control *Mikania* (in part to increase grass resources), further dispersed *Mikania*, increasing dependence on bio-gas.	*Mikania micrantha*, Nepal, Rai and Scarborough 2013

FIRST-TIER ADAPTATION CATEGORY: SOCIO-ECOLOGICAL OUTCOMES<br>Social-ecological outcomes are the effects of change in social-ecological system feedbacks leading to resilience or shifts to alternate states with different controlling variables, ecosystem services, and well-being effects.				
**Second-tier category & definition**	**Third-tier category**	**Third-tier definition**	**Example**	**Case study**
**Resilience** - 'the magnitude of disturbance that can be absorbed before a system changes its structure by changing the variables and processes that control behaviour' (Holling and Gunderson 2002, 28).	**Resilience maintained**	Disturbance is eliminated, or impacts on ecosystems and human well-being are nullified before system becomes unstable.	*Imperata* isn't a serious weed in immature rubber as it is controlled preventatively in this agroforestry system with its greater biodiversity, and more valuable trees.	*Imperata cylindrica*, Indonesia, Bagnall-Oakeley, et al. 1996
	**Resilience renewed**	System becomes unstable, but disturbance is reduced or impacts of disturbance on ecosystems and human wellbeing are managed.	Land pressure and population growth led to dramatically shortened fallows, degrading soils and allowing invasion. *C. odorata* used to stabilise fallow systems, rice yields.	*Chromolaena odorata*, Lao PDR, Roder 1995a, 1995b
	**Resilience decreased**	Unstable system moves closer to boundaries of a regime shift.	Pesticides used to control golden apple snail cause collateral damage and harm native predators, possibly increasing invasion.	Golden apple snail, Ecuador, Horgan et al. 2014
**Regime shift** – Instability increases until system moves to an alternative stable state, with different controlling variables.	**Regime shift**	Major, abrupt changes in the structure and function of a system that moves the system to an alternate state, affecting the ecosystem services provided.	Climate change and other pressures lead to large-scale land abandonment allowing further invasion; increased costs and reduced output leaves farming in deficit, outmigration and abandonment lead to further invasion.	Middle mountains, Nepal, various invasives, Pandey 2017
	**Transformation**	Creation of a fundamentally new system (with a change in state variables) when the existing system is untenable.	Conversion of *Imperata* invaded grassland to cacao agroforestry systems.	*Imperata cylindrica*, Cameroon, Jagoret et al. 2012
